# Inhibitors Targeting SARS‐CoV‐2 Papain‐Like Protease: Screening, Design, Synthesis, and Biological Evaluation

**DOI:** 10.1002/cmdc.202600007

**Published:** 2026-05-08

**Authors:** Elena‐Oriana Iuga, Nilu Gone, Mariana Ortiz de Godoy, Gabriel Correa Verissimo, Giovanna de Jesus Agostinetto, Liam Urich, Nadine Krüger, Thales Kronenberger, Rafael Victorio Carvalho Guido, Anthony J. O’Donoghue, Stefan A. Laufer, Thanigaimalai Pillaiyar

**Affiliations:** ^1^ Institute of Pharmacy Pharmaceutical/Medicinal Chemistry Eberhard Karls University Tübingen Germany; ^2^ Tübingen Center for Academic Drug Discovery (TüCAD_2_) Eberhard Karls University Tübingen Germany; ^3^ São Carlos Institute of Physics University of São Paulo (USP) São Carlos Brazil; ^4^ Faculdade de Farmácia Universidade Federal de Minas Gerais (UFMG) Belo Horizonte Brazil; ^5^ Faculdade de Farmácia Universidade Federal do Rio Grande do Sul Porto Alegre Brazil; ^6^ Skaggs School of Pharmacy and Pharmaceutical Sciences University of California San Diego La Jolla California USA; ^7^ Platform Infection Models German Primate Center Leibniz Institute for Primate Research Göttingen Göttingen Germany; ^8^ School of Pharmacy Faculty of Health Sciences University of Eastern Finland Kuopio Finland; ^9^ German Center for Infection Research (DZIF), partner‐site Tübingen Institute of Medical Microbiology and Hygiene Interfaculty Institute of Microbiology and Infection Medicine (IMIT) University of Tübingen Tübingen Germany; ^10^ Cluster of Excellence “Image Guided and Functionally Instructed Tumor Therapies” (iFIT) Eberhard Karls University of Tuebingen Tuebingen Germany; ^11^ National Center for Tumor diseases South-West (NCT-SW), a partnership between DKFZ University and University Hospital Tübingen, University and University Hospital Ulm and Bosch Health Campus Stuttgart Germany

**Keywords:** antiviral, COVID‐19, non‐covalent inhibitors, papain‐like protease, SARS‐CoV‐2

## Abstract

Despite the availability of vaccines and antiviral drugs, SARS‐CoV‐2 still presents a global health threat, emphasizing the need for new therapeutic targets beyond the main protease and RNA‐dependent RNA polymerase. The papain‐like protease (PL^pro^) is a highly conserved and essential viral enzyme that drives polyprotein processing and suppresses host innate immune responses through its deubiquitinating and deISGylating activities. This study employed two approaches to identify new PL^pro^ inhibitors: (i) the design and synthesis of a range of non‐covalent PL^pro^ inhibitors based on a lead compound, GRL0617. (ii) Screening the in‐house library for new covalent PL^pro^ inhibitors. Biochemical assays demonstrated effective inhibition of PL^pro^ activity, particularly with the non‐covalent inhibitor **2t** (**TPG‐2t**, IC_50_ = 0.634 µM) and with potential covalent inhibitors such as **13e** (IC_50_ = 22.0 µM) and **13f** (**TPG‐13f**, IC_50_ = 5.0 µM). **2t** exhibited antiviral activity (EC_50_ = 2.89 µM) without causing cellular toxicity. Molecular modeling suggests that **2t** can establish stable binding interactions within the PL^pro^ active site. Therefore, inhibitor **2t** could be a promising candidate for future antiviral development.

## Graphical abstract

In our study, we obtained both non‐covalent and covalent PL^pro^ inhibitors. Additionally, the stable protein‐ligand interactions of the active compound were examined using advanced computational docking and molecular dynamics simulations. The non‐covalent inhibitor **2t** showed antiviral activity with an EC_50_ of 2.89 µM, indicating its potential as a promising lead for further structure–activity relationship studies optimization.

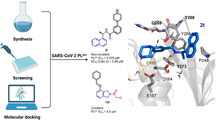

## 1 Introduction

Severe acute respiratory syndrome coronavirus‐2 (SARS‐CoV‐2), the causative agent of the COVID‐19 pandemic, has infected more than 775 million people, leading to 7 million deaths [1]. Despite the availability of vaccines and some approved antiviral drugs [2, 3, 4, 5], emerging variants and declining immunity after prior infections and vaccinations mean that SARS‐CoV‐2 continues to circulate globally, much like influenza and other milder coronaviruses. Furthermore, it still poses substantial risks of causing severe, potentially deadly illnesses and post‐acute sequelae of COVID‐19 (PASC), commonly called long COVID [6]. This significant health burden remains poorly understood and largely unaddressed. It is estimated that over 77 million people have experienced ongoing or new symptoms after the initial SARS‐CoV‐2 infection [7]. Currently, several antiviral drugs that primarily target the RNA‐dependent polymerase (RdRp) or the main protease (M^pro^) of SARS‐CoV‐2 have received clinical approval [8]. Remdesivir is administered intravenously [9], while molnupiravir [10] is an orally available RdRp inhibitor for COVID‐19 infection used to treat mild‐to‐moderate cases. However, their effectiveness was limited [5]. Nirmatrelvir was approved by the FDA in late 2021 and has since been widely used [11]. Nirmatrelvir targets the main protease (M^pro^), an essential enzyme that cleaves viral polypeptides into functional non‐structural proteins. This drug was commercialized under the brand name Paxlovid as a co‐formulation with ritonavir, which acts as a CYP450 inhibitor, thereby boosting its half‐life [12]. Since Paxlovid inhibits CYP3A4, it cannot be safely used by all patients because of potential adverse drug interactions. Recently, several other M^pro^ inhibitors have been reported [13, 14] but none have been approved by the FDA. Furthermore, SARS‐CoV‐2 continues to acquire mutations that enhance viral infectivity and aid immune escape, leading to the emergence of multiple circulating variants [15]. Several of these mutations, especially those that provide resistance to neutralizing antibodies, seem to be under strong positive selection. Recent reports of evolving viral variants and drug resistance highlight the urgent need for alternative therapeutic strategies [16, 17].

SARS‐CoV‐2, along with SARS‐CoV‐1 and MERS‐CoV, belongs to the beta‐coronavirus genus and possesses large positive‐sense RNA genomes of approximately 30 kb that encode numerous structural, accessory, and non‐structural proteins. The first two‐thirds of the viral genome contain the open reading frame (ORF1ab), which encodes the polyproteins pp1a and pp1ab [8, 18]. These are later cleaved by the papain‐like protease (PL^pro^) and M^pro^ into essential non‐structural proteins required for viral replication. PL^pro^ releases several non‐structural proteins (nsp1−3) by cleaving the polyproteins at defined LXGG motifs [19], which are indispensable for SARS‐CoV‐2 replication. Located within residues 746–1060 of nsp3 in SARS‐CoV‐2, the protease comprises 315 amino acids and is highly conserved across coronaviruses [20].

**FIGURE 1:**
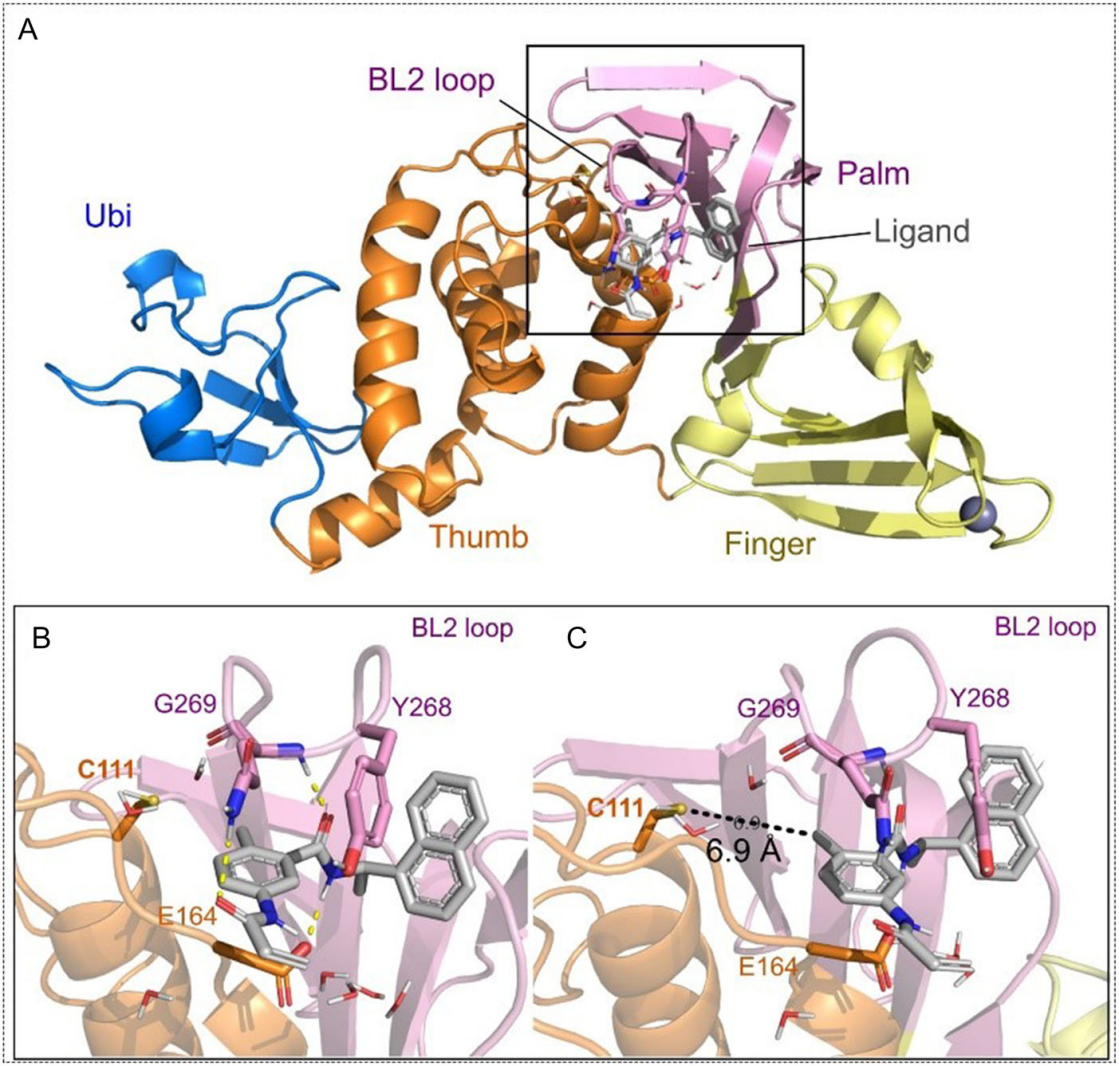
(A) Overview of the SARS‐CoV‐2 PL^pro^ structure bound to GRL0617 (PDB ID 7JIR7JIR) [21]. Highlights of the active site depicting the (B) non‐covalent binding mode of GRL0617 covered by the BL2 loop and (C) the distance between the closest ligand atom to the catalytic cysteine of PL^pro^.

The structure of PL^pro^ comprises three main features: (i) thumb, (ii) fingers, and (iii) palm subdomains, shared with other ubiquitin‐specific proteases, as well as an *N*‐terminal ubiquitin‐like domain that aids in substrate recognition (Figure 1A). The active site, situated at the junction between the thumb and palm subdomains, includes a catalytic triad of Cys111, His273, and Asp287 (Figure 1B,C). The catalytic Cys111 is situated at the bottom of *α*‐helix 4, approximately 3.7 Å away from the catalytic histidine, which is located next to the flexible BL2 loop. This precise spatial arrangement enables the enzyme's proteolytic activity and supports its role in substrate processing [22]. Beyond its proteolytic role, PL^pro^ also counteracts host innate immunity by removing ubiquitin and ISG15 modifications from host proteins that are central to antiviral defense [23, 24]. By removing these post‐translational modifiers, PL^pro^ disrupts ubiquitin‐ and ISG15‐dependent signaling pathways, many of which are required for robust interferon production and downstream immune activation, contributing significantly to virus pathogenicity [23]. This dual function of polyprotein processing, immune modulation, and evasion makes PL^pro^ an attractive antiviral drug target.

**FIGURE 2:**
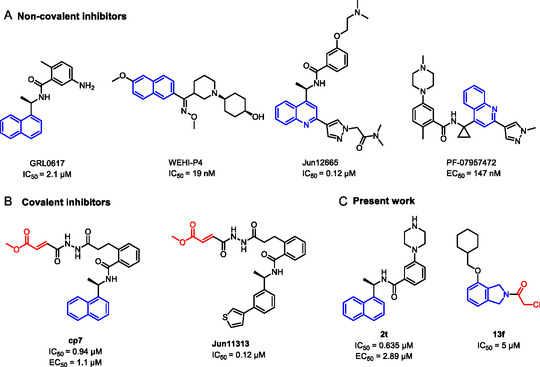
Chemical structure and activity of selected reported PL^pro^ inhibitors. (a) Non‐covalent inhibitors, (b) covalent inhibitors, and (c) inhibitors developed in the present study. * Bicyclic structures are highlighted in blue, and warhead reactive groups are highlighted in red.

Building on progress in targeting SARS‐CoV PL^pro^, many inhibitors for SARS‐CoV‐2 PL^pro^ have been identified (see Figure 2 for representative examples), mainly with a non‐covalent binding mode inspired by the well‐established tool compound GRL0617 [25, 26, 27, 28], along with some covalent inhibitors [29, 30, 31]. GRL0617 was initially designed for SARS‐CoV PL^pro,^ which demonstrated a moderate potency against SARS‐CoV‐1 PL^pro^ (IC_50_ = 15 μM) [32] and SARS‐CoV‐2 PL^pro^ (IC_50_ = 2.1 μM) [33]. Recently, PL^pro^ inhibitors have demonstrated effectiveness in SARS‐CoV‐2 animal models [29, 34], and one GRL‐like compound, known as HL‐21, has progressed to clinical trials [35, 36].

In the present study, we obtained a library of non‐covalent inhibitors based on the lead compound GRL0617 and a library of covalent PL^pro^ inhibitors through screening of our in‐house covalent library. Furthermore, the stable protein‐ligand interactions of the active compound were analyzed through advanced computational docking and molecular dynamics simulations. The non‐covalent inhibitor **2t** demonstrated antiviral activity with an EC_50_ of 2.89 µM and an IC_50_ of 0.634 µM against PL^pro^, highlighting its potential as a promising lead for further structure–activity relationship optimization.

## 2 Results and Discussion

This study aims to identify both non‐covalent and covalent inhibitors of SARS‐CoV‐2 PL^pro^. We employed two strategies: (i) designing and synthesizing different non‐covalent PL^pro^ inhibitors inspired by the lead compound GRL0617 (Section 2.1), and (ii) screening our internal library to discover new covalent PL^pro^ inhibitors (Section 2.5).

### 2.1 Design of New Compounds

**FIGURE 3:**
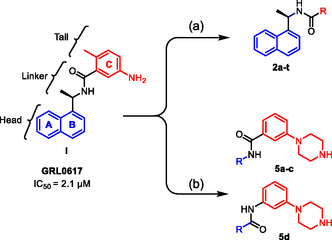
Design of new derivatives and analogs based on GRL0617. (a) tail region replacement; (b) linker and head region replacements. See Schemes 1 and 2, and Table 1 for the structures.

**SCHEME 1:**
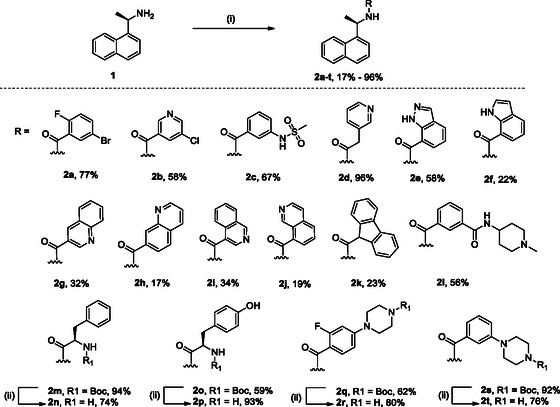
Synthesis of naphthalen‐1‐yl‐ethanamine derivatives **2a‐t**. Reagents and conditions: (i) R‐COOH, HATU, DIPEA, DMF (dry), rt, overnight; (ii) 4 M HCl in dioxane, 0 °C, 2–4h.

**SCHEME 2:**
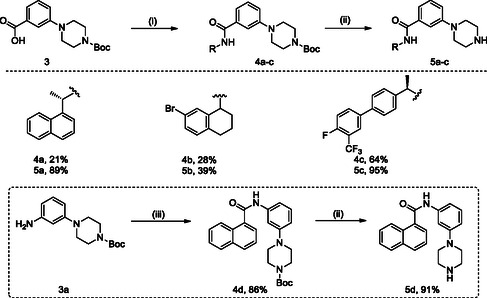
Synthesis of naphthalen‐1‐yl derivatives **5a‐d**. Reagents and conditions: (i) R‐NH_2,_ HATU, DIPEA, DMF (dry), rt, overnight; (ii) 4 M HCl in dioxane, 0 °C, 2–4h; (iii) 1‐naphthoic acid, HATU, DIPEA, DMF (dry), rt, overnight.

GRL0617 represents a valuable tool compound for designing new inhibitors. However, it did not advance to clinical trials due to its low binding affinity. To improve its effectiveness, we conducted an extensive SAR study on GRL0617 as follows (Figure 3): (a) the first one involved replacing the tail region (phenyl ring) with different bicyclic or tricyclic rings to enhance occupancy at the active site (see, for example, **2a**‐**t**, Scheme 1). (b) Next, we investigated the head region (the naphthalene ring) and a linker bearing various substituents (see, for example, **5a**‐**d**, Scheme 2).

### 2.2 Chemistry

The synthesis of all final compounds **2a‐t** and **5a‐d** was accomplished following two synthetic routes (Schemes 1 and 2). In Scheme 1, naphthalen‐1‐yl‐ethanamine was coupled with various substituted aromatic carboxylic acids using HATU in the presence of DIPEA in DMF, affording compounds **2a‐m**, **2o**, **2p**, **2q**, and **2s** in good yields. Subsequent Boc‐deprotection of compounds **2m**, **2o**, **2q**, and **2s** with 4 M HCl in dioxane provided the corresponding free amines **2n**, **2p**, **2r**, and **2t** (Scheme 1, vide infra).

Scheme 2 describes the HATU‐mediated coupling of 3‐(4‐(*tert*‐butoxycarbonyl)piperazin‐1‐yl)benzoic acid (**3**) with several commercially available bicyclic amines to furnish intermediates **4a‐c** in good yields, followed by Boc deprotection to afford target compounds **5a‐c**. Compound **5d** was synthesized by coupling 1‐naphthoic acid with *tert*‐butyl‐4‐(3‐aminophenyl)piperazine‐1‐carboxylate (**3a**), followed by Boc‐group deprotection to yield the final product **5d** (Scheme 2, vide infra).

### 2.3 Pharmacological Evaluation

Following a previously described procedure, SARS‐CoV‐2 PL^pro^ inhibitory activity assays were conducted using fluorogenic substrate Z‐Arg‐Leu‐Arg‐Gly‐Gly‐AMC [37]. The compounds were screened at concentrations of 10 μM (Table 1).

**TABLE 1:** Structures and potency of compounds 2a‐t and **5a‐d** against SARS‐CoV‐2 PL^pro^ activity.

		SARS‐CoV‐2 PL^pro^	
Compound	Structure	% of inhibition at 10 µM	IC_50_, µM
**GRL0617**	see for structure in Figure 2	**57.2**	**0.543**
**2a**	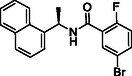	6.9	n.d.
**2b**	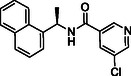	1.4	n.d.
**2c**	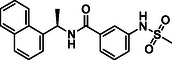	6.4	n.d.
**2d**	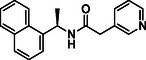	4.3	n.d.
**2e**	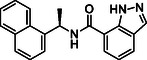	17.2	n.d.
**2f**	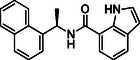	18.2	n.d.
**2g**	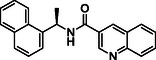	5.8	n.d.
**2hr**	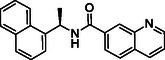	14.8	n.d.
**2i**	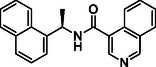	28.9	n.d.
**2j**	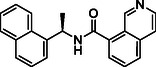	19.2	n.d.
**2k**	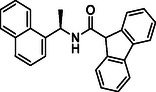	7.3	n.d.
**2l**	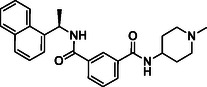	> 30	n.d.
**2m**	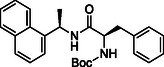	5.1	n.d.
**2n**	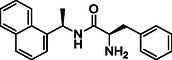	9.3	n.d.
**2o**	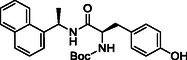	10.9	n.d.
**2p**	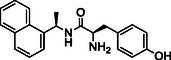	16.1	n.d.
**2q**	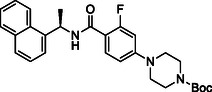	4.5	n.d.
**2r**	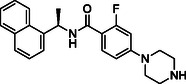	15.1	n.d.
**2s**	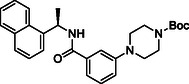	6.5	n.d.
**2t** **(TPG‐2t)**	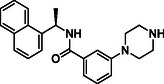	**55.4**	**0.634**
**5a**	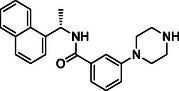	0.0	n.d.
**5b**	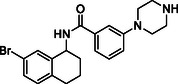	0.0	n.d.
**5c**	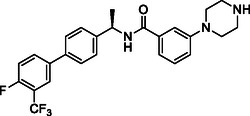	29.6	n.d.
**5d** ^a^	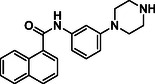	3.5	n.d.

a All assays were performed twice, each in triplicate wells (*n* = 6). n.d. corresponds to not determined.

#### 2.3.1 Structure–Activity Relationship (SAR)

**FIGURE 4:**
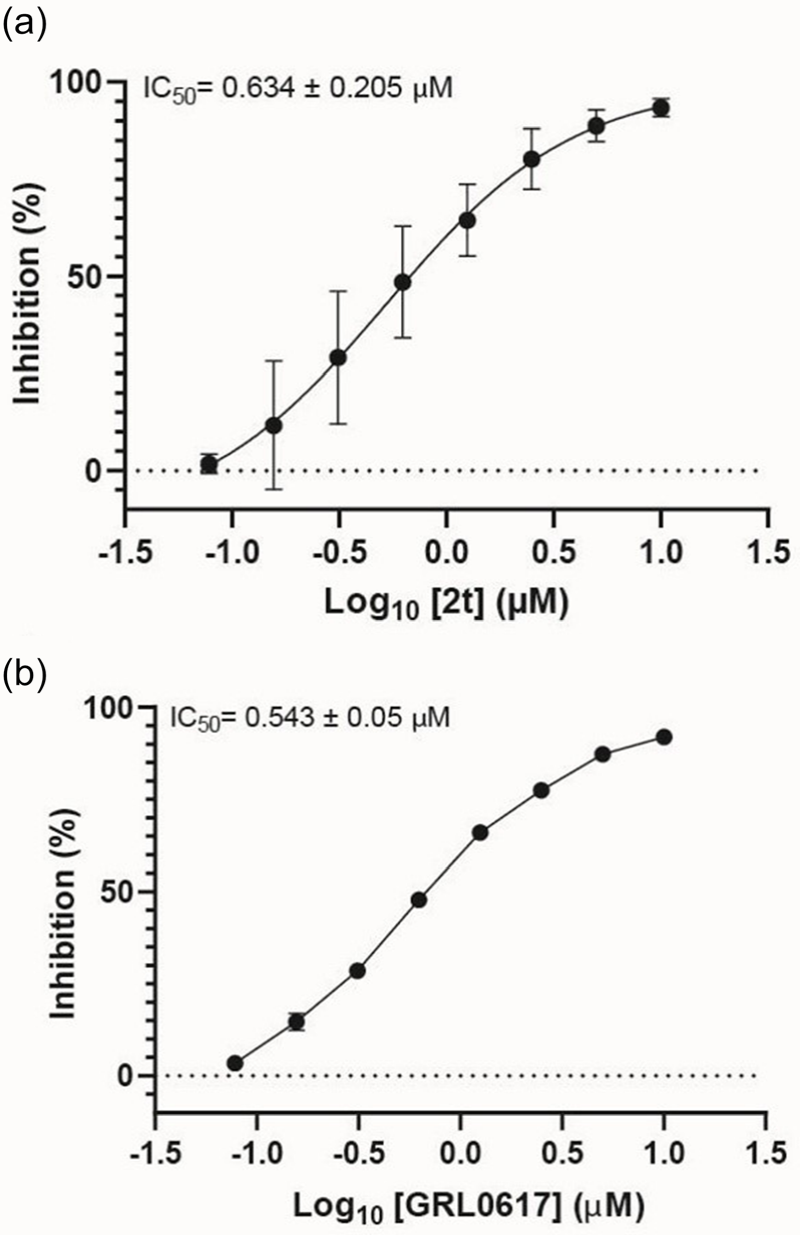
Concentration‐dependent inhibition of (a) SARS‐CoV‐2 PL^pro^ by **2t** compared with (b) GRL0617.

In the present study, extensive structural modifications were carried out to improve the activity of the lead molecule GRL0617 (Table 1). We initially focused on modifying the tail of the lead molecule by replacing aniline with various residues, including 5‐bromo‐2‐fluorobenzene (**2a**), 5‐chloropyridine (**2b**), *N*‐phenylmethanesulfonamide (**2c**), and 3‐methylpyridine (**2d**). However, all changes led to a complete abolition of the PL^pro^ inhibitory activity. Next, we introduced bicyclic, such as 1H‐indazole (**2e**), 1H‐indole (**2f**), quinoline (**2g**, **2h**), isoquinoline (**2i**, **2j**), and tricyclic, such as 9H‐fluorene (**2k**). These derivatives exhibited only weak to moderate PL^pro^ inhibition at 10 μM, ranging from 5.8% to 28.9%. Notably, compounds with an isoquinoline connecting carbonyl group at either the 4‐ or 8‐ position demonstrated slightly better inhibition than other substitutes. However, the inhibition was not adequate to calculate IC_50_ values. Introducing amino acids such as phenylalanine (**2m**, **2n**) and tyrosine (**2o**, **2p**) also did not improve PL^pro^ inhibition. We next introduced 2‐fluorophenyl‐4‐piperazine, which showed a 15.1% inhibition at 10 μM. Surprisingly, when we moved piperazine from position 4–3 on the phenyl ring, the compound **2t** inhibited PL^pro^ activity by 55.4% at 10 µM. Protecting the NH in piperazine with the Boc group, as seen in **2q** and **2s**, resulted in the loss of PL^pro^ inhibitory activity. These findings reveal that having piperazine at the 3‐position, with a free NH on the phenyl ring, is crucial for activity. A concentration–response curve was then generated for the most potent compound (**2t** or **TPG‐2t**), and an IC_50_ value of 0.634 µM was calculated. In parallel, the IC_50_ of the parent compound GRL017 was determined to be 0.543 µM (Figure 4).

In the subsequent approach, we targeted the linker and head region of the lead molecule. To assess the stereocenter's significance, the *R*‐methyl group in **2t** was replaced with an *S*‐methyl group (**5a**), thereby completely abolishing activity. This likely occurs because the *S*‐isomer interferes with the molecule's binding orientation at the PL^pro^ active site. Furthermore, replacing naphthalene with 4‐fluoro‐3‐(trifluoromethyl)−1,1^′^‐biphenyl (**5c**) or removing the linker, as in **5b** and **5d**, also led to the loss of activity.

#### 2.3.2 Cytotoxicity and Antiviral Activity of 2t Against SARS‐CoV‐2

**FIGURE 5:**
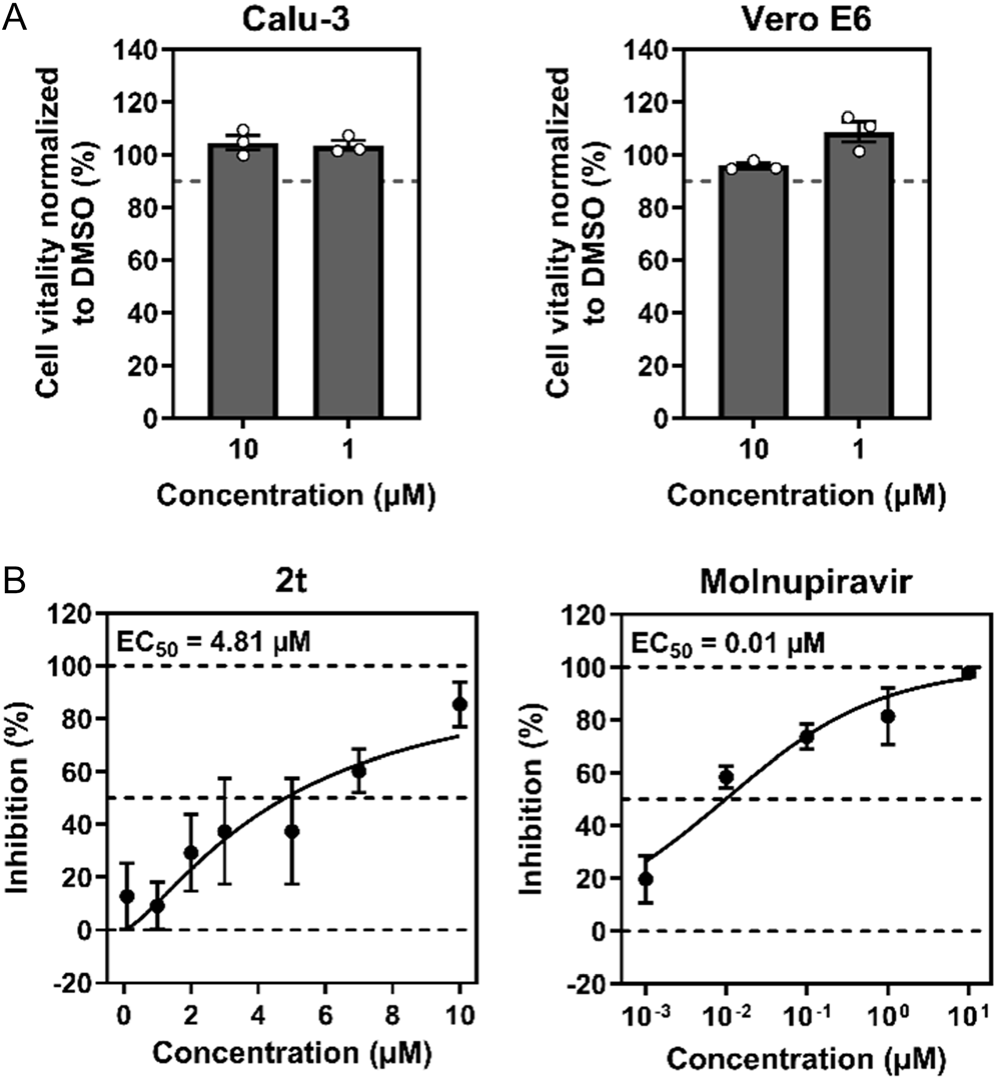
(A) Cell vitality of Calu‐3 and Vero E6 cells incubated with 10 and 1 µM of inhibitor **2t**. Cell vitality was normalized to luminescence values obtained from cells incubated with DMSO (set to 100%). The graphs shown represent the mean ± SEM of three biological replicates, each performed as triplicates. Circles indicate the means of each independent experiment. (B) Calu‐3 cells were incubated with serial dilutions of inhibitor **2t** or molnupiravir, followed by infection with SARS‐CoV‐2. Viral titers were determined by titration on Vero E6 cells and normalized to those of DMSO‐treated cells (set at 0% inhibition). The graphs shown mean ± SEM of three biological replicates.

The antiviral activity of a highly potent PL^pro^ inhibitor, **2t**, against SARS‐CoV‐2 was evaluated, but first, the cytotoxicity of the test compound was assessed (see Figure 5). This step is important because a decrease in cell virion production caused by the toxicity of the test compounds can be mistaken for antiviral activity. Therefore, the compound's impact on the viability of Calu‐3 and Vero E6 cells was measured by comparing ATP levels in inhibitor‐treated cells to those in DMSO‐treated controls (solvent controls). As shown in Figure 5A, the inhibitor did not reduce cell viability at either 1 or 10 µM, indicating that the observed decrease in viral titers is not due to nonspecific cytotoxicity. Next, antiviral activity against SARS‐CoV‐2, Pango lineage B.1.513, was determined. The inhibitor **2t** exhibited antiviral activity with an EC_50_ of 4.81 μM.

### 2.4 Computational Studies

#### 2.4.1 Molecular Interaction of 2t With Papain‐Like Protease

**FIGURE 6:**
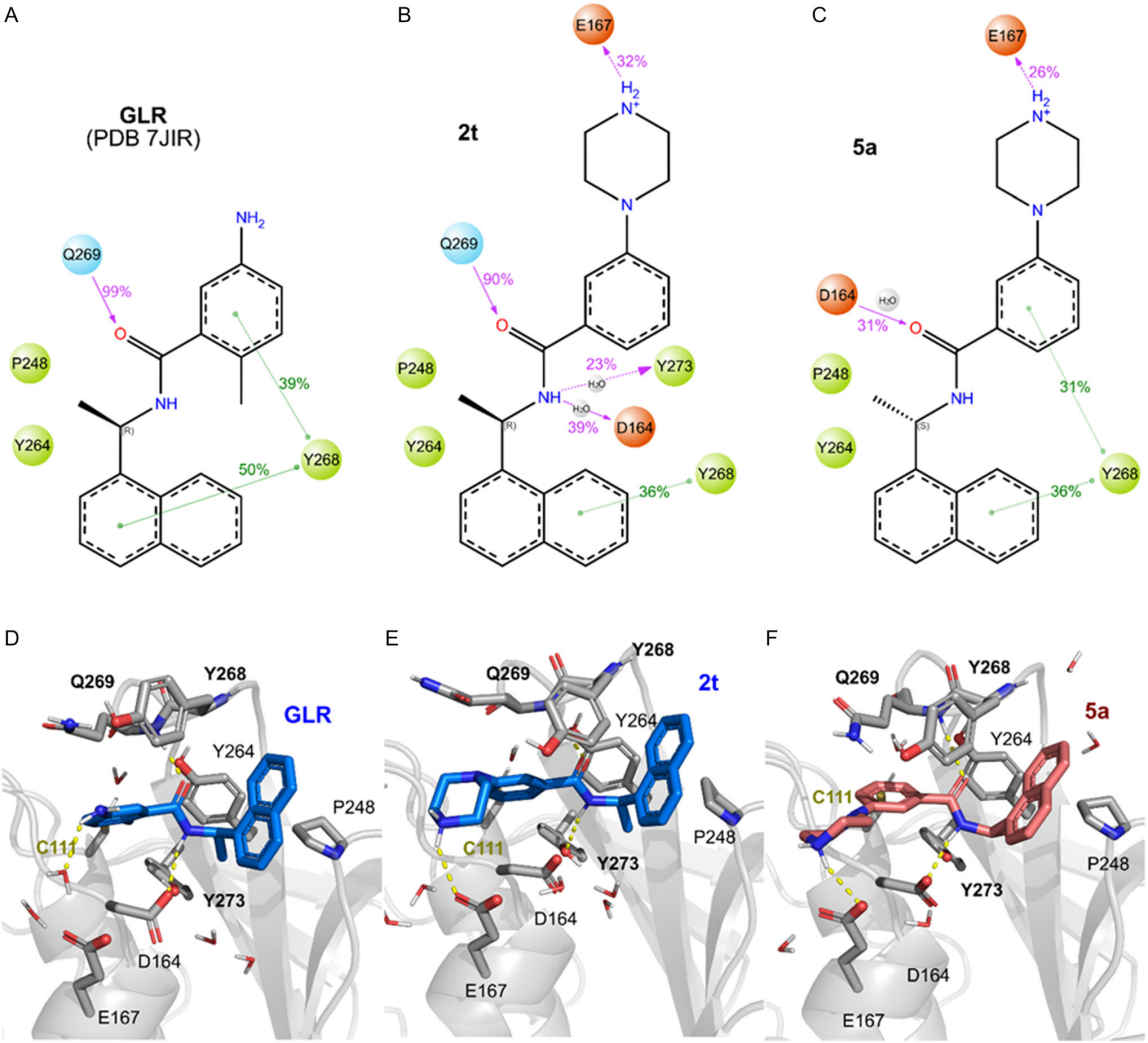
(A–C) Protein–ligand interaction frequencies plotted over the chemical structure. Representative snapshot of the simulations of PL^pro^ with the (D) co‐crystallized ligand GLR0617, (E) active inhibitor **2t**, and (F) inactive isomer **5a**. Conserved motifs’ residues are depicted in bold.

We identified possible binding modes for ligand **2t** and its inactive *R*‐isomer **5a** through a combination of docking and short MD simulations (5 x 200 ns) and then compared these with simulations of the original co‐crystallized ligand GRL0617 (PDB 7JIR) [21]. We calculated the protein–ligand interaction frequency and predicted binding energy from those trajectories (Figure 6A–C). The most common conformations retrieved from the simulations by clustering are similar to the GRL0617 binding mode (Figure 6D–E). Their amide linker interacting with the backbone atoms of Gln269 and the loop covering the activation loop retained a closed conformation, favoring π‐mediated interactions between naphthalene and Tyr268. Our compound **2t**, which has a piperazine ring instead of the amino group found in GRL0617, engages the solvent‐exposed Glu167 via H‐bonds. Since simulations indicate no major differences in predicted binding energy between active and inactive isomers, we focused on analyzing their interaction frequencies. In Figure 6F, the inactive isomer **5a** loses the key H–bond interaction to Gln269.

### 2.5 Screening of the Covalent Library

**FIGURE 7:**
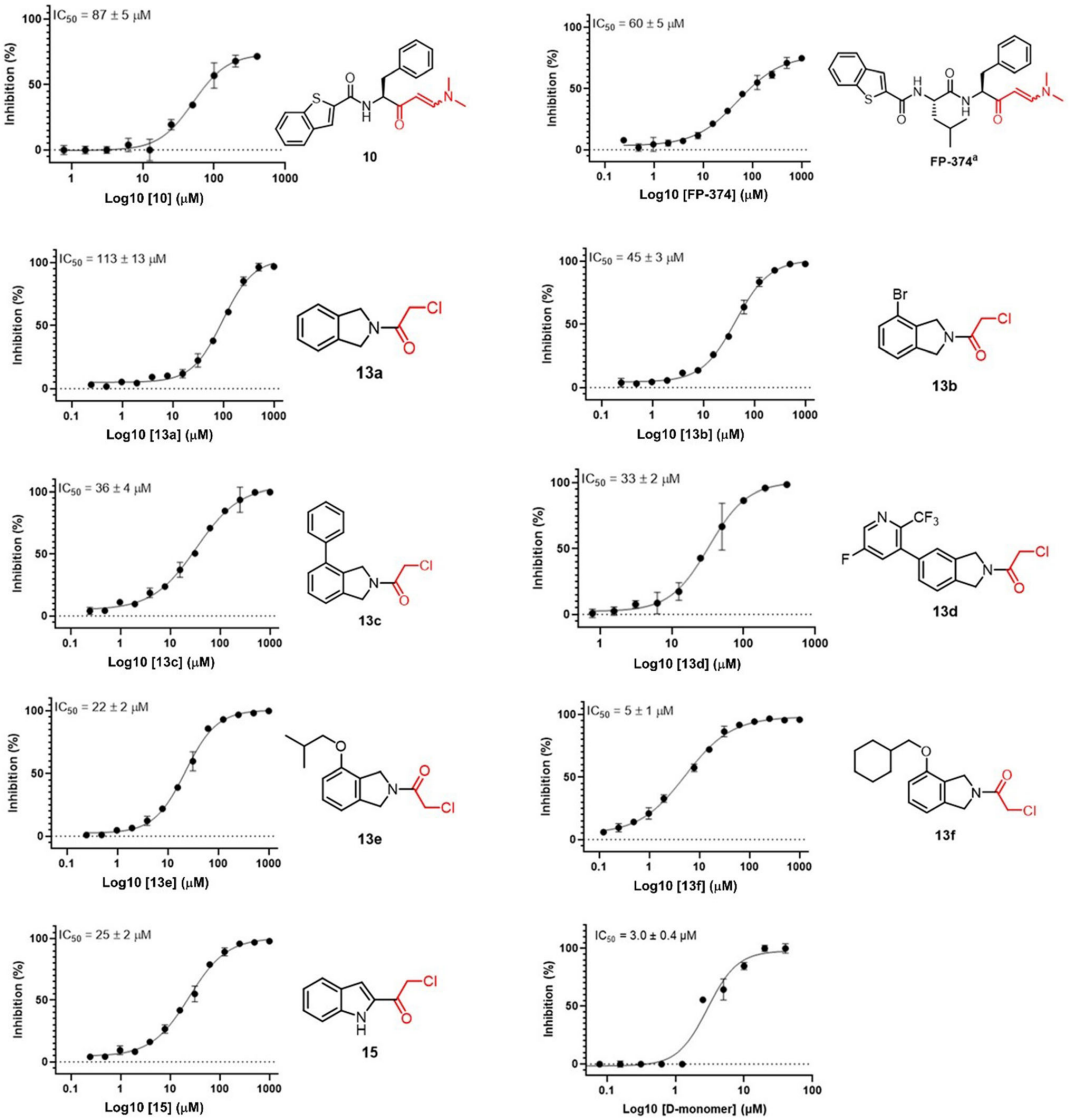
Chemical structures of compounds identified from the in‐house covalent library and their concentration‐dependent inhibition of SARS‐CoV‐2 PL^pro^, compared with *D*‐monomer used as the positive control. * Warhead reactive groups are highlighted in red.

In this study, we evaluated our proprietary covalent library, containing 38 molecules, against PL^pro^. All compounds were initially tested at 100 µM using a FRET‐based enzyme assay with the fluorogenic substrate Abz‐TLKGG APIKEDDPS‐EDDnp [30, 38]. For compounds that achieved at least 50% inhibition of PL^pro^ at 100 μM, we generated concentration‐response curves (IC_50_ values) using *D*‐monomer as a positive control (IC_50_ = 3 μM). Of the 38 molecules tested, nine compounds inhibited PL^pro^ in the micromolar range, from 5 to 113 µM (Figure 7).

These molecules are isoindoline and indoline derivatives bearing a chloromethyl ketone, which would serve as a covalent warhead that reacts with the active Cys111 of PL^pro^. **10** and **FP‐374** are low‐molecular‐weight peptidomimetics containing (*E*)‐3‐(dimethylamino)acrylketone, which would undergo Michael addition with the catalytic Cys111. It was interesting to observe the clear SAR trend in the isoindoline derivatives. The unsubstituted derivative (**13a**, IC_50_ = 113 µM) showed weak PL^pro^ inhibitory activity, while substitutions like 4‐Br (**13b**, IC_50_ = 45 µM), 4‐phenyl (**13c**, IC_50_ = 36 µM), 4‐cyclohexylmethoxy (**13f** or **TPG‐13f**, IC_50_ = 5.0 µM), and 4‐isobutoxy (**13e**, IC_50_ = 22 µM) enhance the inhibitory activity, with **13f** being the most potent PL^pro^ inhibitor among them, indicating that larger hydrophobic substituents are important for inhibitory activity improvement. A substituent at position 5, as in **13d** (IC_50_ = 33 µM), also exhibits similar inhibitory activity levels (compare **13c** vs. **13d**). Interestingly, the indole derivative **15** (IC_50_ = 25 µM) showed moderate activity but was more than four times as potent as the corresponding isoindoline **13a** (IC_50_ = 113 µM), suggesting that the indoline scaffold (as in **15**) could be a promising starting point for an extensive SAR study to improve potency against PL^pro^.

The Michael acceptors **10** and **FP‐374** exhibited moderate PL^pro^ inhibition with IC_50_ values of 87 and 60 µM, respectively. It is of note that **FP‐374** and **10** did not achieve complete inhibition of protein activity, even at the maximum tested concentration (400 µM). Additionally, the shape of their concentration–response curves indicates that the maximum inhibition both compounds can achieve under these assay conditions is around 70%–75% (see Figure 6). These results suggest they might bind to an allosteric binding site [39], where the binding of the first molecule may hinder the second from binding to the other site, possibly through an allosteric communication that prevents the second site from functioning or induces conformational changes that lock the enzyme. However, this will be confirmed in future studies. Except for **FP‐374**, all the active compounds are newly discovered. **FP‐374** (**12b** in the publication) [40] was previously identified as a SARS‐CoV‐2 M^pro^ inhibitor with an IC_50_ value of 18.9 µM. Thus, **FP‐374** is a dual M^pro^ and PL^pro^ inhibitor of SARS‐CoV‐2. The concentration–response curves for all active compounds are depicted in Figure 7.

## 3 Conclusions

PL^pro^ is an important target for developing antivirals because it modulates viral replication and host's innate immune responses through its deubiquitinating and deISGylating activities [23]. In this study, we employed two approaches to identify both non‐covalent and covalent inhibitors of PL^pro^. The first approach involved the design and synthesis of a range of non‐covalent PL^pro^ inhibitors based on a lead compound, GRL0617. This led to the identification of a non‐covalent inhibitor (*R*)‐*N*‐(1‐(naphthalen‐1‐yl)ethyl)‐3‐(piperazin‐1‐yl)benzamide (**2t**, IC_50_ = 0.634 µM), which also exhibited antiviral activity (EC_50_ = 2.89 µM) without causing cellular toxicity. However, its *S*‐isomer **5a** was inactive, suggesting that stereochemistry plays a key role in enzyme inhibition. Docking and molecular dynamics simulations of **2t** revealed stable, favorable interactions within the PL^pro^ active site. Consequently, inhibitor **2t** may serve as a promising candidate for future antiviral development.

In the second approach, we screened our in‐house covalent library to identify covalent inhibitors of PL^pro^. This led to the identification of several inhibitors in the micromolar range, from 5 to 113 µM. In particular, 4‐(cyclohexylmethoxy)isoindoline (**13f**) was found to be the most potent, with an IC_50_ of 5.0 µM. SAR analysis indicated that larger hydrophobic substituents at positions 4 or 5 on the isoindoline moiety were important for improving activity. Additionally, the indoline scaffold was identified as a potential starting point for an extensive SAR study aimed at improving potency against PL^pro^. Docking studies of **10** and **13d** revealed a covalent bond interaction with the Cys111 pf PL^pro^ active site. Covalent inhibition, an effective approach for targeting cysteine proteases, offers advantages over noncovalent methods, including higher target affinity, the need for lower concentrations due to longer residence time on the target [41, 42], reduced sensitivity to pharmacokinetic factors, and decreased vulnerability to drug resistance [41, 43, 44].

## 4 Experimental Section

### 4.1 Chemistry

All commercially available starting materials, reagents, and (anhydrous) solvents were used without further purification. Reaction controls were performed by thin‐layer chromatography (TLC) on Macherey–Nagel‐precoated 60 F254 silica plates. Spots were visualized either by ultraviolet (UV) light (254 nm) or staining solutions. Flash column chromatography was carried out using Grace Davison Davisil LC60A (20–45 μm) or Merck Geduran Si60 (mesh 63–200 μm) with a LaFlash automated flash chromatography system. NMR spectra were recorded on a Bruker Avance 400 MHz spectrometer at ambient temperature. Chemical shifts (δ) are reported in parts per million (ppm) relative to the internal control tetramethylsilane (TMS), and the spectra were calibrated against the residual solvent peak of the used deuterated solvent. Coupling constants (*J*) are expressed in Hz. Purities of final compounds were determined by RP‐HPLC using an Agilent 1100 Series LC with a Phenomenex Luna C8 analytical column (150 × 4.6 mm, 5 μm) and detected by a UV‐DAD detector at 254 and 230 nm wavelengths. The eluting was carried out with the following gradient: (*A* = 0.01 M KH_2_PO_4_, pH 2.30, *B* = MeOH) 40% *B* to 85% *B* in 8 min, 85% *B* for 5 min, 85% to 40% *B* in 1 min, 40% *B* for 2 min, stop time 16 min, flow 1.5 mL/min. Standard mass spectra were obtained from an Advion Expression Compact mass spectrometer (electron spray ionization, ESI) with a TLC plate reader system (using the following settings: ESI voltage 3.50 kV, capillary voltage 187 V, source voltage 44 V, capillary temperature 250°C, desolvation gas temperature 250°C, gas flow 5 L/min). All final compounds are ≥95% pure by HPLC.

### 4.2 General Procedure I for the HATU‐Mediated Amide Coupling

The corresponding carboxylic acid (1 eq) and the amine (3 eq) were dissolved in anhydrous DMF (5 mL). Sequentially, HATU (1.2 eq) and diisopropylethylamine (DIPEA) (3 eq) were added to the solution. The resulting mixture was stirred overnight at room temperature. The reaction was quenched by adding water and extracted with EtOAc (3 × 15 mL). The combined organic phases were washed with sat. aq. HCl 2 M solution (1 × 25 mL), sat. aq. NaHCO_3_ solution (1 × 25 mL) and brine (1 × 15 mL). The white solid compound was purified by column chromatography.

### 4.3 General Procedure II for the Boc Deprotection

The Boc‐protected compounds **2m**, **2o**, **2q**, and **2s** (see Scheme 1) and **4a‐d** (see Scheme 2) were subjected to deprotection using a 4 M HCl in dioxane for 3h at 0°C to room temperature. After completion of the reaction, the mixture was concentrated under reduced pressure in order to obtain the HCl salt as a white solid. No further purification by column chromatography was needed.

### 4.4 (*R*)‐5‐Bromo‐2‐fluoro‐*N*‐(1‐(naphthalen‐1‐yl)ethyl)benzamide (2a)

This product was obtained following the general procedure **I** from the reaction of (*R*)‐1‐(naphthalen‐1‐yl)ethanamine (450 mg, 2.63 mmol) with 5‐bromo‐2‐fluorobenzoic acid (576 mg, 2.63 mmol). Yield: 755 mg (77.2%) of **2a** as a white solid. ^1^H NMR (400 MHz, DMSO‐*d*_6_) δ ^1^H NMR (400 MHz, DMSO) δ 9.13 (d, *J* = 7.6 Hz, 1H), 8.19 (d, *J* = 8.4 Hz, 1H), 7.96 (d, *J* = 7.9 Hz, 1H), 7.85 (d, *J* = 8.1 Hz, 1H), 7.73–7.66 (m, 2H), 7.61 (t, *J* = 6.1 Hz, 1H), 7.57 (d, *J* = 6.9 Hz, 1H), 7.54–7.47 (m, 2H), 7.29 (t, *J* = 9.1 Hz, 1H), 2.54 (s, 1H), 1.57 (d, *J* = 6.9 Hz, 3H). ^13^C NMR (101 MHz, DMSO‐*d*_6_) δ 161.69, 159.52, 157.03, 139.86, 134.70, 133.41, 132.02, 130.29, 128.73, 127.39, 126.80, 126.27, 125.62, 123.07, 122.47, 118.63, 115.99, 45.04, 21.56. ESI‐MS [M+H]^+^ = 373.1. HPLC t_*R*_ = 8.431 min.

### 4.5 (*R*)‐5‐Chloro‐*N*‐(1‐(naphthalen‐1‐yl)ethyl)nicotinamide (2b)

This product was obtained following the general procedure **I** from the reaction of (*R*)‐1‐(naphthalen‐1‐yl)ethanamine (500 mg, 2.92 mmol) and 5‐chloronicotinic acid (460 mg, 2.92 mmol). Yield: 526 mg (58%) of **2b** as a white solid. ^1^H NMR (400 MHz, DMSO‐*d*_6_) δ 9.27 (d, *J* = 7.6 Hz, 1H), 8.99 (d, *J* = 1.8 Hz, 1H), 8.78 (d, *J* = 2.3 Hz, 1H), 8.38 (t, *J* = 2.1 Hz, 1H), 8.19 (d, *J* = 8.4 Hz, 1H), 7.95 (m, 1H), 7.84 (d, *J* = 8.1 Hz, 1H), 7.65 (d, *J* = 7.0 Hz, 1H), 7.58 (m, 1H), 7.53 (s, 2H), 5.96 (p, *J* = 7.0 Hz, 1H), 1.63 (d, *J* = 6.9 Hz, 3H).^13^C NMR (101 MHz, DMSO‐*d*_6_) δ 162.59, 150.40, 147.01, 139.80, 134.65, 133.38, 131.06, 130.99, 130.33, 128.70, 127.40, 126.28, 125.63, 125.53, 123.00, 122.62, 45.08, 39.52, 21.40. ESI‐MS [M + Na]^+^ = 333. HPLC t_*R*_ = 7.76 min.

### 4.6 (*R*)‐3‐(Methylsulfonamido)‐*N*‐(1‐(naphthalen‐1‐yl)ethyl)benzamide (2c)

This product was obtained following the general procedure **I** from the reaction of (*R*)‐1‐(naphthalen‐1‐yl)ethanamine (500 mg, 2.92 mmol) with 3‐(methylsulfonamido)benzoic acid (628.45 mg 2.92 mmol). Yield: 718.7 mg (66.79%) of **2c** as a light‐yellow solid. ^1^H NMR (400 MHz, DMSO‐*d*_6_) δ 1H NMR (400 MHz, DMSO‐*d*_6_) δ 9.69 (s, 1H), 8.99 (d, *J* = 7.8 Hz, 1H), 8.20 (d, *J* = 8.3 Hz, 1H), 7.95 (d, *J* = 7.3 Hz, 1H), 7.84 (d, *J* = 8.1 Hz, 1H), 7.65 (m, 3H), 7.55 (m, 3H), 7.39 (m, 2H), 5.95 (p, *J* = 7.0 Hz, 1H), 3.00 (s, 3H), 1.61 (d, *J* = 6.9 Hz, 3H). ^13^C NMR (101 MHz, DMSO‐*d*_6_) δ 165.18, 140.29, 138.52, 135.83, 133.38, 130.44, 129.15, 128.68, 127.27, 126.20, 125.59, 125.48, 123.12, 122.60, 122.53, 122.31, 119.21, 44.78, 39.38, 21.42. ESI‐MS [M + Na]^+^ = 391.2. HPLC t_*R*_ = 6.817 min.

### 4.7 (*R*)‐*N*‐(1‐(Naphthalen‐1‐yl)ethyl)‐2‐(pyridin‐3‐yl)acetamide (2d)

This product was obtained following the general procedure **I** from the reaction of (*R*)‐1‐(naphthalen‐1‐yl)ethanamine (565 mg, 3.3 mmol) with 2‐(pyridin‐3‐yl)acetic acid (452.5 mg, 3.3 mmol). Yield: 917 mg (95.7%) of **2d** as a white solid. ^1^H NMR (500 MHz, DMSO‐*d*_6_) δ 8.76 (d, *J* = 7.8 Hz, 1H), 8.43 (m, 2H), 8.05 (m, 1H), 7.93 (m, 1H), 7.82 (d, *J* = 8.0 Hz, 1H), 7.65 (d, *J* = 7.8 Hz, 1H), 7.51 (m, 4H), 7.31 (m, 1H), 5.68 (p, *J* = 7.0 Hz, 1H), 1.51 (d, *J* = 6.9 Hz, 3H), 1.24 (m, 2H). ^13^C NMR (101 MHz, DMSO‐*d*_6_)) δ 168.46, 149.89, 147.55, 139.90, 136.43, 133.35, 132.07, 130.34, 128.61, 127.32, 126.12, 125.60, 125.39, 123.27, 123.05, 122.31, 44.14, 39.20, 21.45. ESI‐MS [M + Na]^+^ = 313.1. HPLC t_*R*_ = 4.396 min.

### 4.8 (*S*)‐*N*‐(Naphthalen‐1‐yl)ethyl)‐1‐H‐indazole‐7‐carboxamide (2e)

This product was obtained following the general procedure **I** from the reaction of (*R*)‐1‐(naphthalen‐1‐yl)ethanamine (450 mg, 2.63 mmol) with 1H‐7‐indazole‐carboxylic acid (426.11 mg, 2.63 mmol). Yield: 496 mg (57.8%) of **2e** as an off‐white colored solid. ^1^H NMR (400 MHz, DMSO‐*d*_6_) δ 13.06 (s, 1H), 9.19 (d, *J* = 7.6 Hz, 1H), 8.28 (d, *J* = 8.4 Hz, 1H), 8.13 (d, *J* = 1.3 Hz, 1H), 8.09 (d, *J* = 7.2 Hz, 1H), 7.96 (m, 2H), 7.83 (d, *J* = 8.1 Hz, 1H), 7.68 (d, *J* = 7.1 Hz, 1H), 7.55 (m, 3H), 7.21 (t, *J* = 7.6 Hz, 1H), 6.07 (d, *J* = 6.9 Hz, 1H), 1.67 (d, *J* = 6.9 Hz, 3H). ^13^C NMR (101 MHz, DMSO‐*d*_6_) δ 165.04, 140.63, 137.84, 133.50, 133.38, 130.41, 128.68, 127.20, 126.19, 125.57, 125.55, 124.93, 124.39, 124.33, 123.08, 122.53, 119.50, 116.92, 44.73, 21.59. ESI‐MS [M + Na]^+^ = 338.3. HPLC t_*R*_ = 7.719 min.

### 4.9 (*R*)‐*N*‐(1‐(Naphthalen‐1‐yl)ethyl)‐1H‐indole‐7‐carboxamide (2f)

This product was obtained using the general procedure **I** from the reaction of (*R*)‐1‐(naphthalen‐1‐yl)ethanamine (171.24 mg, 1.64 mmol) with indole‐7‐carboxylic acid (263.34 mg, 1.64 mmol). Yield: 115 mg (22.4%) of **2f** as a dark beige solid. ^1^H NMR (400 MHz, DMSO‐*d*_6_) δ 11.13 (s, 1H), 9.05 (d, *J* = 7.7 Hz, 1H), 8.29 (d, *J* = 8.4 Hz, 1H), 7.94 (m, 1H), 7.83 (t, *J* = 7.4 Hz, 2H), 7.74 (d, *J* = 7.8 Hz, 1H), 7.68 (d, *J* = 7.0 Hz, 1H), 7.54 (m, 3H), 7.29 (m, 1H), 7.08 (t, *J* = 7.6 Hz, 1H), 6.47 (m, 1H), 6.06 (p, *J* = 7.0 Hz, 1H), 1.66 (d, *J* = 7.0 Hz, 3H). ^13^C NMR (101 MHz, DMSO‐*d*_6_) δ 166.20, 140.83, 134.25, 133.37, 130.44, 129.10, 128.66, 127.12, 126.52, 126.13, 125.53, 123.80, 123.11, 122.57, 120.15, 117.96, 116.76, 100.93, 54.91, 21.57. ESI‐MS [M + Na]^+^ = 337.2. HPLC t_*R*_ = 8.039 min.

### 4.10 (*R*)‐*N*‐(1‐(Naphthalen‐1‐yl)ethyl)quinoline‐3‐carboxamide (2g)

This product was obtained using the general procedure **I** from the reaction of (*R*)‐1‐(naphthalen‐1‐yl)ethanamine (504 mg, 2.94 mmol) and quinoline‐3‐carboxylic acid (509.68 mg, 2.94 mmol). Yield: 308.3 mg (32%) of **2g** as a white solid. ^1^H NMR (400 MHz, DMSO‐*d*_6_) δ 9.35 (d, *J* = 7.7 Hz, 1H), 9.32 (d, *J* = 2.2 Hz, 1H), 8.90 (d, *J* = 1.9 Hz, 1H), 8.25 (d, *J* = 8.4 Hz, 1H), 8.10 (dd, *J* = 7.6, 5.7 Hz, 2H), 7.96 (d, *J* = 7.8 Hz, 1H), 7.87 (dd, *J* = 11.8, 4.8 Hz, 2H), 7.69 (dd, *J* = 12.3, 4.0 Hz, 2H), 7.56 (m, 3H), 6.04 (p, *J* = 6.9 Hz, 1H), 1.67 (d, *J* = 6.9 Hz, 3H).^13^C NMR (101 MHz, DMSO‐*d*_6_) δ 164.59, 149.58, 148.95, 140.56, 136.04, 133.88, 131.62, 130.90, 129.57, 129.24, 129.18, 127.87, 127.84, 127.50, 126.97, 126.75, 126.11, 126.02, 123.58, 123.11, 45.40, 21.98. ESI‐MS [M + Na]^+^ = 349.2. HPLC t_*R*_ = 7.706 min

### 4.11 (*R*)‐*N*‐(Naphthalen‐1‐yl)ethyl)quinoline‐7‐carboxamide (2h)

This product was obtained following the general procedure **I** from the reaction of (*R*)‐1‐(naphthalen‐1‐yl)ethanamine (280 mg, 1.64 mmol) with 7‐quinoline carboxylic acid (283.16 mg, 1.64 mmol). Yield: 90.8 mg (17%) of **2h** as a brown solid. ^1^H NMR (400 MHz, DMSO‐*d*_6_) δ 11.13 (s, 1H), 9.05 (d, *J* = 7.7 Hz, 1H), 8.29 (d, *J* = 8.4 Hz, 1H), 7.95 (d, *J* = 7.4 Hz, 1H), 7.83 (t, *J* = 7.4 Hz, 2H), 7.73 (d, *J* = 7.8 Hz, 1H), 7.68 (d, *J* = 7.1 Hz, 1H), 7.54 (m, 3H), 7.29 (s, 2H), 7.08 (t, *J* = 7.6 Hz, 1H), 6.47 (dd, *J* = 3.0, 2.1 Hz, 1H), 6.04 (m, 1H), 1.66 (d, *J* = 7.0 Hz, 3H). ^13^C NMR (101 MHz, DMSO‐*d*_6_) δ 166.20, 140.84, 134.26, 133.37, 130.44, 129.10, 128.66, 127.12, 126.52, 126.13, 125.53, 125.53, 125.53, 123.80, 123.11, 122.58, 120.15, 117.97, 116.77, 100.93, 44.52, 21.57. ESI‐MS MS [M + H]^+^ = 327.2. HPLC t_*R*_ = 8.067 min.

### 4.12 (*R*)‐*N*‐(Naphthalen‐1‐yl)ethyl)isoquinoline‐4‐carboxamide (2i)

This product was obtained following the general procedure **I** from the reaction of (*R*)*‐*1‐(naphthalen‐1‐yl)ethanamine (350 mg, 2.04 mmol) with isoquinoline‐4‐carboxylic acid (353.94 mg, 2.04 mmol). Yield: 225.1 mg (33.75%) of **2i** as a white solid. ^1^H NMR (400 MHz, DMSO‐*d*_6_) δ 9.40 (m, 2H), 8.66 (s, 1H), 8.31 (d, *J* = 7.9 Hz, 1H), 8.20 (d, *J* = 7.2 Hz, 2H), 7.98 (d, *J* = 7.4 Hz, 1H), 7.84 (dd, *J* = 15.5, 7.6 Hz, 2H), 7.69 (m, 3H), 7.56 (m, 2H), 6.06 (s, 1H), 1.67 (d, *J* = 5.5 Hz, 3H). ^13^C NMR (101 MHz, DMSO‐*d*_6_) δ 165.61, 154.02, 141.15, 140.08, 133.48, 132.47, 131.60, 130.44, 128.78, 128.19, 127.91, 127.60, 127.42, 126.30, 125.71, 125.71, 125.61, 124.30, 123.17, 122.58, 44.88, 21.54. ESI‐MS [M + Na]^+^ = 349.4. HPLC t_*R*_ = 7.132 min.

### 4.13 (*R*)‐*N*‐(1‐(Naphthalen‐1‐yl)ethyl)isoquinoline‐8‐carboxamide (2j)

This product was obtained following the general procedure **I** from the reaction of (*R*)‐1‐(naphthalen‐1‐yl)ethanamine (450 mg, 2.63 mmol) and isoquinoline‐8‐carboxylic acid (454.63 mg, 2.63 mmol). Yield: 159 mg (18.55%) of **2j** as a white solid. ^1^H NMR (400 MHz, DMSO‐*d*_6_) δ 9.47 (s, 1H), 9.36 (d, *J* = 7.7 Hz, 1H), 8.54 (d, *J* = 5.6 Hz, 1H), 8.33 (d, *J* = 8.4 Hz, 1H), 8.06 (d, *J* = 7.9 Hz, 1H), 7.99 (d, *J* = 8.0 Hz, 1H), 7.89 (d, *J* = 4.7 Hz, 2H), 7.80 (m, 2H), 7.66 (m, 2H), 7.55 (dt, *J* = 12.2, 7.7 Hz, 2H), 6.08 (p, *J* = 6.7 Hz, 1H), 1.67 (d, *J* = 6.8 Hz, 3H). ^13^C NMR (101 MHz, DMSO‐*d*_6_) δ 166.36, 149.95, 142.94, 140.03, 135.32, 134.85, 133.43, 130.44, 129.65, 128.73, 128.44, 127.38, 126.66, 126.26, 125.67, 125.51, 124.95, 123.12, 122.51, 120.58, 44.77, 21.42.ESI‐MS [M + Na]^+^ = 349. HPLC t_*R*_ = 6.11 min.

### 4.14 (*R*)‐*N*‐(1‐(Naphthalen‐1‐yl)ethyl)‐9H‐fluorene‐9 carboxamide (2k)

This product was obtained following the general procedure **I** from the reaction of (*R*)‐1‐(naphthalen‐1‐yl)ethanamine (500 mg, 2.92 mmol) and 9H‐Fluorene‐9‐carboxylic acid (613.85 mg, 2.92 mmol). Yield: 245 mg (23%) of **2k** as a white solid. ^1^H NMR (400 MHz, DMSO‐*d*_6_) δ 9.15 (d, *J* = 7.7 Hz, 1H), 8.09 (d, *J* = 5.3 Hz, 1H), 7.97 (m, 1H), 7.87 (t, *J* = 6.6 Hz, 3H), 7.73 (d, *J* = 7.0 Hz, 1H), 7.57 (m, 4H), 7.39 (m, 4H), 7.25 (t, *J* = 7.4 Hz, 1H), 5.75 (m, 1H), 4.88 (s, 1H), 1.62 (s, 3H). ^13^C NMR (101 MHz, DMSO‐*d*_6_) δ 168.57, 143.12, 143.02, 141.38, 141.38, 139.60, 133.41, 130.50, 128.65, 128.65, 127.61, 127.56, 127.49, 127.19, 127.05, 126.16, 125.68, 125.46, 124.66, 124.57, 123.22, 122.63, 120.08, 54.42, 44.39, 21.30. ESI‐MS [M + Na]^+^ = 386.2; [M + H + Na]^+^ = 387.1. HPLC t_*R*_ = 9.106 min.

### 4.15 (*R*)‐*N* ^1^‐(1‐Methylpiperidin‐4‐yl)‐*N* ^3^‐(1‐(naphthalen‐1‐yl)ethyl)isophthalamide (2l)

This product was obtained following the general procedure **I** from the reaction of (*R*)‐3‐((1‐(naphthalen‐1‐yl)ethyl)carbamoyl)benzoic acid (260 mg, 0.81 mmol) with (*R*)‐*N* ^1^‐(1‐methylpiperidin‐4‐yl)‐*N* ^3^‐(1‐(naphthalen‐1‐yl)ethyl)isophthalamide (93 mg, 0.81 mmol). Yield: 190 mg (56%) of **2l** as a yellow solid. ^1^H NMR (400 MHz, DMSO‐*d*_6_) δ 9.12 (d, *J* = 7.8 Hz, 1H), 8.39 (d, *J* = 7.8 Hz, 1H), 8.31 (t, *J* = 1.8 Hz, 1H), 8.22 (d, *J* = 8.4 Hz, 1H), 8.01 (dt, *J* = 7.8, 1.5 Hz, 1H), 7.96 (dd, *J* = 7.7, 1.7 Hz, 2H), 7.85 (d, *J* = 8.1 Hz, 1H), 7.65 (d, *J* = 7.1 Hz, 1H), 7.62–7.47 (m, 4H), 5.98 (p, *J* = 7.0 Hz, 1H), 3.80–3.68 (m, 1H), 2.77 (d, *J* = 11.5 Hz, 2H), 2.17 (s, 3H), 1.95 (t, *J* = 11.5 Hz, 2H), 1.80–1.72 (m, 2H), 1.64 (d, *J* = 6.9 Hz, 3H), 1.62–1.51 (m, 2H). ^13^C NMR (101 MHz, DMSO‐*d*_6_) δ 165.85, 165.66, 140.74, 135.47, 135.12, 133.87, 130.96, 130.32, 129.16, 128.59, 127.76, 127.06, 126.68, 125.97, 123.63, 123.16, 54.93, 47.07, 46.36, 45.29, 31.82, 21.89. ESI‐MS [M + H]^+^ = 416.3. HPLC t_*R*_ = 3.750 min.

### 4.16 *Tert*‐butyl ((*R*)‐1‐(((*R*)‐1‐(Naphthalen‐1‐yl)ethyl)amino)‐1‐oxo‐3‐phenylpropan‐2‐Yl)carbamate (2m)

This product was obtained following the general procedure **I** from the reaction of (*R*)‐1‐(naphthalen‐1‐yl)ethanamine (570 mg, 3.33 mmol) with (*R*)‐2‐((*tert*‐butoxycarbonyl)amino)‐3‐phenylpropanoic acid (883.1 mg, 3.33 mmol). Yield: 1312 mg (94.18%) of **2m** as a white solid. ^1^H NMR (400 MHz, DMSO‐*d*_6_) δ 8.42 (d, *J* = 7.7 Hz, 1H), 8.10 (t, *J* = 13.9 Hz, 1H), 7.93 (m, 1H), 7.83 (d, *J* = 8.0 Hz, 1H), 7.51 (m, 4H), 7.27 (d, *J* = 4.3 Hz, 4H), 7.21 (m, 1H), 6.90 (d, *J* = 8.6 Hz, 1H), 5.64 (m, 1H), 4.23 (dd, *J* = 14.1, 8.9 Hz, 1H), 2.93 (dd, *J* = 13.5, 5.2 Hz, 1H), 2.79 (dd, *J* = 13.4, 9.6 Hz, 1H), 1.41 (d, *J* = 6.8 Hz, 3H), 1.30 (s, 9H). ^13^C NMR (101 MHz, DMSO‐*d*_6_) δ 170.65, 155.16, 139.71, 138.05, 133.35, 130.41, 129.27, 129.27, 128.57, 128.00, 128.00, 127.27, 126.19, 126.10, 125.55, 125.34, 123.28, 122.44, 77.95, 55.78, 44.18, 37.55, 28.10, 28.10, 28.10, 21.37. ESI‐MS [M + Na]^+^ = 441.2. HPLC t_*R*_ = 9.176 min.

### 4.17 (*R*)‐2‐Amino‐*N*‐((R)‐naphthalen‐1‐yl)ethyl)‐3‐phenylpropanamide (2n)

This product was obtained following the general procedure **II** from the reaction of *tert*‐butyl ((*R*)‐1‐(((*R*)‐1‐(naphthalen‐1‐yl)ethyl)amino)‐1‐oxo‐3‐phenylpropan‐2‐yl)carbamate (508 mg, 1.21 mmol) with 4N HCl (3.10 ml, 12.14 mmol). Yield 320.1 mg (74.27%) of **2n** as a light beige solid. ^1^H NMR (400 MHz, DMSO‐*d*_6_) δ 9.08 (d, *J* = 7.8 Hz, 1H), 8.40 (s, 2H), 8.09 (d, *J* = 8.1 Hz, 1H), 7.94 (d, *J* = 7.4 Hz, 1H), 7.84 (d, *J* = 8.1 Hz, 1H), 7.51 (m, 4H), 7.34 (m, 5H), 5.62 (p, *J* = 6.7 Hz, 1H), 4.10 (t, *J* = 7.0 Hz, 1H), 3.13 (m, 2H), 1.28 (t, *J* = 15.1 Hz, 3H). ^13^C NMR (101 MHz, DMSO‐*d*_6_)) δ 166.73, 139.19, 135.07, 133.30, 130.12, 129.66, 128.65, 128.65, 128.45, 127.45, 127.10, 126.32, 125.62, 125.45, 123.15, 123.00, 66.35, 53.38, 44.60, 37.19, 21.46. ESI‐MS [M + Na]^+^ = 341.3. HPLC t_*R*_ = 5.908 min.

### 4.18 *Tert*‐butyl ((*R*)‐3‐(4‐Hydroxyphenyl)‐1‐(((*R*)‐1‐(naphthalen‐1‐yl)ethyl)amino)‐1‐oxopropan‐2‐yl)carbamate (2o)

This product was obtained following the general procedure **I** from the reaction of (*R*)‐1‐(naphthalen‐1‐yl)ethanamine (350 mg, 2.04 mmol) with (*R*)‐2‐((*tert*‐butoxycarbonyl)amino)‐3‐(4‐hydroxyphenyl)propanoic acid (574.95 mg, 2.04 mmol). Yield: 528 mg (59.5%) of **2o** as a white solid. ^1^H NMR (400 MHz, DMSO‐*d*_6_) δ 9.16 (s, 1H), 8.36 (d, *J* = 7.7 Hz, 1H), 8.08 (d, *J* = 8.9 Hz, 1H), 7.93 (m, 1H), 7.82 (d, *J* = 8.1 Hz, 1H), 7.51 (m, 4H), 7.04 (d, *J* = 8.3 Hz, 2H), 6.80 (d, *J* = 8.5 Hz, 1H), 6.65 (d, *J* = 8.3 Hz, 2H), 5.63 (p, *J* = 6.6 Hz, 1H), 4.13 (dd, *J* = 14.2, 8.8 Hz, 1H), 2.81 (m, 1H), 2.67 (dd, *J* = 13.5, 9.4 Hz, 1H), 1.41 (d, *J* = 6.8 Hz, 3H), 1.31 (s, 9H). ^13^C NMR (101 MHz, DMSO‐*d*_6_) δ 170.80, 155.74, 155.15, 139.77, 133.34, 130.41, 130.16, 130.16, 128.57, 128.04, 127.25, 126.09, 125.54, 125.34, 123.29, 122.45, 114.80, 114.80, 77.92, 59.75, 56.11, 44.17, 39.52, 28.13, 28.13, 28.13, 21.42. ESI‐MS [M + Na]^+^ = 457.2. HPLC t_*R*_ = 7.909 min.

### 4.19 (*R*)‐2‐Amino‐3‐(4‐hydroxyphenyl)‐*N*‐((*R*)‐1‐(naphthalen‐1‐yl)ethyl)propanamide (2p)

This product was obtained following the general procedure **II** from the reaction of *tert*‐butyl ((*R*)‐3‐(4‐hydroxyphenyl)‐1‐(((*R*)‐1‐(naphthalen‐1‐yl)ethyl)amino)‐1‐oxopropan‐2‐yl)carbamate (210 mg, 0.48 mmol) with 4N HCl (0.12 mL, 0.48 mmol). Yield: 151 mg (93.2%) of **2p** as a white solid. ^1^H NMR (400 MHz, DMSO‐*d*_6_) δ 9.46 (s, 1H), 9.06 (d, *J* = 7.8 Hz, 1H), 8.30 (s, 2H), 8.10 (d, *J* = 8.2 Hz, 1H), 7.94 (m, 1H), 7.83 (d, *J* = 8.1 Hz, 1H), 7.54 (m, 4H), 7.10 (d, *J* = 8.4 Hz, 2H), 6.76 (d, *J* = 8.4 Hz, 2H), 5.63 (p, *J* = 6.7 Hz, 1H), 4.00 (d, *J* = 4.5 Hz, 1H), 3.00 (m, 2H), 1.35 (d, *J* = 6.8 Hz, 3H). ^13^C NMR (101 MHz, DMSO‐*d*_6_) δ 166.91, 156.60, 139.28, 133.30, 130.58, 130.13, 130.13, 128.65, 127.44, 126.31, 125.62, 125.46, 124.91, 123.18, 123.01, 115.26, 66.35, 53.67, 44.65, 36.45, 21.54. ESI‐MS [M + Na]^+^ = 357.4. HPLC t_*R*_ = 3.789 min.

### 4.20 (*R*)‐Tert‐butyl 4‐(3‐Fluoro‐4‐((1‐(naphthalen‐1‐yl)ethyl)carbamoyl)phenyl) piperazine‐1‐carboxylate (2q)

This product was obtained following the general procedure **I** from the reaction of (*R*)‐1‐(naphthalen‐1‐yl)ethanamine (67 mg, 0.39 mmol) with (*R*)‐*tert*‐butyl 4‐(3‐fluoro‐4‐((1‐(naphthalen‐1yl)ethyl)carbamoyl)phenyl)piperazine‐1‐carboxylate (126.91 mg 0.39 mmol). Yield 115.7 mg (61.87%) of **2q** as a white solid. ^1^H NMR (400 MHz, DMSO‐*d*_6_) δ 8.42 (d, *J* = 4.3 Hz, 1H), 8.20 (d, *J* = 8.0 Hz, 1H), 7.95 (d, *J* = 7.6 Hz, 1H), 7.83 (d, *J* = 7.9 Hz, 1H), 7.63 (d, *J* = 6.8 Hz, 1H), 7.52 (m, 3H), 6.77 (d, *J* = 12.9 Hz, 2H), 5.91 (m, 1H), 5.76 (s, 1H), 3.44 (s, 4H), 3.25 (s, 4H), 1.58 (d, *J* = 6.4 Hz, 3H), 1.42 (s, 9H). ^13^C NMR (101 MHz, DMSO‐*d*_6_) δ 162.69, 162.15, 159.70, 153.80, 153.75, 153.64, 140.43, 133.37, 131.14, 130.33, 128.65, 127.18, 126.12, 125.55, 125.49, 123.12, 122.48, 112.64, 112.50, 110.02, 101.39, 101.12, 79.07, 54.90, 46.85, 44.70, 28.03, 21.63. ESI‐MS [M + Na]^+^ = 500.5. HPLC t_*R*_ = 9.584 min.

### 4.21 (*R*)‐2‐Fluoro‐*N*‐(1‐(naphthalen‐1‐yl)ethyl)‐4‐(piperazin‐1‐yl)benzamide (2r)

This product was obtained following the general procedure **II** from the reaction of (*R*)‐*tert*‐butyl 4‐(3‐fluoro‐4‐((1‐(naphthalen‐1‐yl)ethyl)carbamoyl)phenyl) piperazine‐1‐carboxylate (65 mg, 0.14 mmol) with 4N HCl (0.35 mL, 1.4 mmol). Yield 41 mg (80.39%) of **2r** as a white solid. ^1^H NMR (400 MHz, DMSO‐*d*_6_) δ 9.45 (s, 2H), 8.51 (d, *J* = 5.3 Hz, 1H), 8.19 (d, *J* = 8.3 Hz, 1H), 7.95 (d, *J* = 7.7 Hz, 1H), 7.83 (d, *J* = 8.1 Hz, 1H), 7.61 (t, *J* = 8.4 Hz, 1H), 7.52 (m, 3H), 6.85 (m, 2H), 5.89 (dd, *J* = 13.9, 6.8 Hz, 1H), 3.52 (s, 5H), 3.17 (s, 4H), 1.58 (d, *J* = 6.8 Hz, 3H). ^13^C NMR (101 MHz, DMSO‐*d*_6_) δ 162.63, 162.04, 159.59, 152.94, 152.83, 140.38, 133.36, 131.20, 130.31, 128.66, 127.20, 126.13, 125.56, 125.49, 123.11, 122.49, 113.56, 110.32, 101.86, 44.73, 44.20, 42.05, 21.61. ESI‐MS [M + Na]^+^ = 400.2. HPLC t_*R*_ = 5.457 min.

### 4.22 (*R*)‐Tert‐butyl 4‐(3‐((1‐(Naphtalen‐1‐yl)ethyl)carbamoyl)phenyl)piperazine‐1‐carboxylate (2s)

This product was obtained following the general procedure **I** from the reaction of (*R*)‐1‐(naphthalen‐1‐yl)ethanamine (80 mg, 0.47 mmol) and 3‐(4‐(tert‐Butoxycarbonyl)piperazin‐1‐yl)benzoic acid (143.13 mg, 0.47 mmol). Yield 199 mg (92.5%) of **2s** as a white solid. ^1^H NMR (400 MHz, DMSO‐*d*_6_) δ 8.88 (d, *J* = 7.8 Hz, 1H), 8.20 (d, *J* = 8.3 Hz, 1H), 7.95 (m, 1H), 7.84 (d, *J* = 8.1 Hz, 1H), 7.64 (d, *J* = 7.0 Hz, 1H), 7.55 (m, 3H), 7.44 (s, 1H), 7.36 (d, *J* = 7.7 Hz, 1H), 7.30 (t, *J* = 7.9 Hz, 1H), 7.10 (dd, *J* = 8.1, 1.6 Hz, 1H), 5.96 (p, *J* = 6.9 Hz, 1H), 3.47 (m, 4H), 3.14 (m, 4H), 1.62 (d, *J* = 6.9 Hz, 3H), 1.42 (s, 9H). ^13^C NMR (101 MHz, DMSO‐*d*_6_) δ 166.69, 154.80, 138.27, 135.68, 134.12, 134.12, 131.38, 129.41, 128.93, 128.66, 126.81, 126.05, 125.33, 123.60, 122.85, 119.55, 117.90, 115.73, 80.16, 49.34, 49.34, 45.35, 45.35, 43.41, 28.55, 28.55, 28.55, 20.77. ESI‐MS [M + Na]^+^ = 482.4. HPLC t_*R*_ = 9.017 min.

### 4.23 (*R*)‐*N*‐(1‐(Naphthalen‐1‐yl)ethyl)‐3‐(piperazin‐1‐yl)benzamide (2t)

This product was obtained following the general procedure **II** from the reaction of (*R*)‐*tert*‐butyl 4‐(3‐((1‐(naphthalen‐1‐yl)ethyl)carbamoyl)phenyl)piperazine‐1‐carboxylate (75 mg, 0.16 mmol) with 4N HCl in dioxane (0.4 mL, 1.63 mmol). Yield: 45 mg (76.27%) of **2t** as a white solid. ^1^H NMR (400 MHz, DMSO‐*d*_6_) δ 9.34 (s, 2H), 8.94 (d, *J* = 7.8 Hz, 1H), 8.20 (d, *J* = 8.3 Hz, 1H), 7.94 (d, *J* = 7.7 Hz, 1H), 7.83 (d, *J* = 8.1 Hz, 1H), 7.65 (d, *J* = 7.1 Hz, 1H), 7.56 (m, 1H), 7.49 (dd, *J* = 13.0, 4.9 Hz, 2H), 7.42 (d, *J* = 7.7 Hz, 1H), 7.33 (t, *J* = 7.9 Hz, 1H), 7.14 (dd, *J* = 8.1, 1.8 Hz, 1H), 5.96 (p, *J* = 6.9 Hz, 1H), 3.42 (m, 4H), 3.21 (s, 4H), 1.62 (d, *J* = 6.9 Hz, 3H). ^13^C NMR (101 MHz, DMSO‐*d*_6_) δ 165.49, 149.94, 140.34, 135.31, 133.36, 130.46, 128.97, 128.63, 127.22, 126.13, 125.54, 125.45, 123.14, 122.64, 119.13, 118.76, 114.74, 45.41, 45.41, 44.73, 44.73, 42.50, 21.40. ESI‐MS [M + H]^+^ = 360.1. HPLC t_*R*_ = 4.417 min.

### 4.24 (*S*)‐*N*‐(1‐Naphthalen‐1‐yl)ethyl)‐3‐(piperazin‐1‐yl)benzamide (5a)

This product was obtained following the general procedure **II** from the reaction of (*S*)‐*tert*‐butyl 4‐(3‐((1‐(naphthalen‐1‐yl)ethyl)carbamoyl)phenyl)piperazine‐1‐carboxylate (36 mg, 0.08 mmol) with 4N HCl (0.2 mL, 0.8 mmol). Yield: 25 mg (89%) of **5a** as a yellow solid. ^1^H NMR (400 MHz, MeOH‐*d*_4_) δ 8.07 (d, *J* = 8.0 Hz, 1H), 7.77 (d, *J* = 7.7 Hz, 1H), 7.68 (d, *J* = 7.7 Hz, 1H), 7.53 (d, *J* = 6.1 Hz, 1H), 7.41 (m, 5H), 7.29 (s, 1H), 7.18 (s, 1H), 5.94 (d, *J* = 5.9 Hz, 1H), 3.42 (s, 4H), 3.29 (s, 4H), 1.61 (d, *J* = 6.2 Hz, 3H). ^13^C NMR (101 MHz, MeOH‐*d*_4_) δ 169.08, 150.44, 140.42, 136.78, 135.33, 132.22, 130.72, 129.85, 128.85, 127.20, 126.62, 126.46, 124.08, 123.72, 122.09, 121.67, 117.38, 48.22, 46.87, 46.87, 44.57, 44.57, 21.41. ESI‐MS [M + Na]^+^ = 382.1. HPLC t_*R*_ = 5.099 min.

### 4.25 *Tert*‐butyl 4‐(3‐((7‐Bromo‐1,2,3,4‐tetrahydronaphthalen‐1‐yl)carbamoyl)phenyl)piperazine‐1‐carboxylate (5b)

This product was obtained following the general procedure **II** from the reaction of 7‐Bromo‐1,2,3,4‐tetrahydro‐1‐naphthalenamine (100 mg, 0.44 mmol) with 3‐(4‐(*tert*‐butoxycarbonyl)piperazin‐1‐yl)benzoic acid (135.5 mg, 0.44 mmol). The product was purified by column chromatography and deprotected during the process. Yield: 71.8 mg (39%) of **5b** as a white solid. ^1^H NMR (400 MHz, MeOH‐*d*_4_) δ 7.54 (d, *J* = 1.8 Hz, 1H), 7.42 (ddd, *J* = 15.5, 6.9, 1.5 Hz, 3H), 7.31 (dd, *J* = 8.2, 1.9 Hz, 1H), 7.25 (m, 1H), 7.07 (d, *J* = 8.2 Hz, 1H), 5.32 (t, *J* = 6.7 Hz, 1H), 3.52 (dd, *J* = 6.5, 3.9 Hz, 4H), 3.41 (dd, *J* = 6.4, 3.8 Hz, 4H), 3.33 (dd, *J* = 3.3, 1.6 Hz, 2H), 2.78 (m, 2H), 2.07 (m, 2H). ^13^C NMR (101 MHz, MeOH‐*d*_4_) δ 169.91, 151.72, 140.71, 138.11, 136.65, 132.09, 131.70, 131.08, 130.61, 121.29, 120.95, 120.40, 116.87, 49.34, 47.65, 47.65, 44.72, 44.72, 30.89, 29.81, 21.79. ESI‐MS [M + Na]^+^ = 436. HPLC t_*R*_ = 5.853 min.

### 4.26 (*R*)‐*N*‐(1‐(4^′^‐Fluoro‐3^′^‐(trifluoromethyl)‐[1,1^′^‐biphenyl]‐4‐yl)ethyl)‐3‐(piperazin‐1‐yl)benzamide (5c)

This product was obtained following the General Procedure **II** from the reaction of (*R*)‐*tert*‐butyl 4‐(3‐((1‐(4^′^‐fluoro‐3^′^‐(trifluoromethyl)‐[1,1′‐biphenyl]‐4‐yl)ethyl)carbamoyl)phenyl)piperazine‐1 carboxylate (45 mg, 0.08 mmol) and 4N HCl (0.2 mL, 0.8 mmol). Yield: 35 mg (95%) of **5c** as a brown oil. ^1^H NMR (400 MHz, MeOH‐*d*_4_) δ 7.82 (m, 2H), 7.56 (d, *J* = 7.0 Hz, 2H), 7.48 (d, *J* = 4.9 Hz, 3H), 7.37 (m, 3H), 7.22 (s, 1H), 5.24 (d, *J* = 6.1 Hz, 1H), 3.48 (s, 4H), 3.36 (s, 4H), 1.57 (d, *J* = 6.3 Hz, 3H). ^13^C NMR (101 MHz, MeOH‐*d*_4_) δ 169.55, 161.57, 159.05, 151.38, 145.48, 138.98, 138.54, 136.74, 133.99, 130.62, 128.14, 128.07, 128.07, 126.21, 126.21, 121.34, 121.18, 118.62, 118.41, 117.02, 50.51, 47.85, 47.85, 44.87, 44.87, 22.16. ESI‐MS [M + Na]^+^ = 494.1. HPLC t_*R*_ = 7.289 min.

### 4.27 *N*‐(3‐(Piperazin‐1‐yl)phenyl)‐1‐naphthamide (5d)

This product was obtained following the procedure **II** from the reaction of *tert*‐butyl 4‐(3‐(1‐naphthamido)phenyl)piperazine‐1‐carboxylate (160 mg, 0.37 mmol) with 4N HCl (0.925 mL, 3.7 mmol). Yield: 112 mg (91%) of **5d** as a white solid. ^1^H NMR (400 MHz, MeOH‐*d*_4_) δ 8.16 (m, 1H), 7.96 (d, *J* = 8.2 Hz, 1H), 7.89 (m, 1H), 7.67 (d, *J* = 6.8 Hz, 1H), 7.61 (s, 1H), 7.50 (m, 3H), 7.25 (m, 2H), 6.88 (d, *J* = 7.6 Hz, 1H), 3.46 (d, *J* = 4.9 Hz, 4H), 3.37 (d, *J* = 4.8 Hz, 4H). ^13^C NMR (101 MHz, MeOH‐*d*_4_) δ 170.81, 151.37, 141.10, 135.81, 135.19, 131.78, 131.40, 130.87, 130.77, 129.54, 128.15, 127.53, 126.49, 126.06, 126.00, 115.33, 114.69, 110.70, 48.32, 48.32, 44.59. ESI‐MS [M + Na]^+^ = 354. HPLC t_*R*_ = 4.043 min.

### 4.28 *Tert*‐butyl‐(*S*)‐(2‐(Methoxy(methyl)amino)‐2‐Oxo‐1‐phenylethyl)carbamate (6)

A mixed suspension of (*S*)‐2‐((tert‐butoxycarbonyl)amino)‐2‐phenylacetic acid (251 mg, 1.0 mmol), *N*, *O‐*dimethylhydroxylamine hydrochloride (146 mg, 1.5 mmol) and DIPEA (0.57 mL, 3.5 eq) in CH_2_Cl_2_ (7 mL) were cooled to 0°C, followed by dropwise addition of T_3_P (451 mg, 1.5 eq). The resulting mixture was stirred at room temperature for 2h. The reaction was concentrated under reduced pressure, diluted with ethyl acetate (EtOAc, 20 mL), and washed successively with saturated aqueous sodium bicarbonate (NaHCO_3_, 2 × 15 mL) and brine (15 mL). The organic layer was dried over anhydrous sodium sulfate (Na_2_SO_4_), filtered, concentrated under reduced pressure and purification using Flash chromatography with PE/ EtOAc (0%–40% EtOAc). Yield: 202 mg (97%) of **6** as a colorless solid. ^1^H NMR (400 MHz, CDCl_3_) δ 7.33–7.27 (m, 3H), 7.25–7.17 (m, 2H), 5.75–5.67 (m, 1H), 5.67–5.59 (m, 1H), 3.39 (s, 3H), 3.10 (s, 3H), 1.34 (s, 9H). ESI‐MS [M + Na]^+^ = 317.4, HPLC t_*R*_ = 7.55 min.

### 4.29 (*S*)‐2‐Amino‐*N*‐methoxy‐*N* *‐*methyl‐2‐phenylacetamide (7)

The *N*‐Boc protected amine (250 mg, 1 equiv.) was dissolved in CH_2_Cl_2_ (3 mL), and 4 N HCl in dioxane (10 eq) was added at 0°C. The mixture was stirred for 3h at 0°C. The reaction was concentrated under reduced pressure to yield the HCl salt, which was then dissolved in aqueous 0.05 M NaOH and extracted with EtOAc (3 × 15 mL). The combined organic layers were dried over anhydrous Na_2_SO_4_, filtered, and concentrated under reduced pressure. The product was used without further purification for the next step. Yield: 165 mg (100%) of **7** as a white solid. ^1^H NMR (400 MHz, DMSO‐*d*_6_) δ 8.87 (s, 3H), 7.51–7.39 (m, 5H), 5.32 (s, 1H), 3.47 (s, 3H), 3.13 ppm (s, 3H). ESI‐MS [M + Na]^+^ = 217.3, HPLC t_*R*_ = 1.48 min.

### 4.30 (*S*)‐*N*‐Methoxy‐*N*‐methyl‐3‐phenyl‐2‐(2‐phenylacetamido)propanamide (8)

HATU (261 mg, 1.2 equiv.) was added sequentially at 0°C to a solution of 2‐phenylacetic acid (102 mg, 1 eq) in DMF (5 mL). The solution was kept at 0°C for 30 min. After 30 min, DIPEA (0.3 mL, 3 equiv.) and the amine (184 mg, 1 eq) were added slowly. The mixture was stirred at 0°C for 1 h and at 25°C for 12h. The reaction was quenched by adding water and extracted with EtOAc (3 × 15 mL). The combined organic phases were washed with sat. NH_4_Cl solution (2 × 50 mL), sat. NaHCO_3_ solution (2 × 50 mL) and brine (2 × 50 mL). The organic layer was dried over anhydrous Na_2_SO_4_ and concentrated in vacuo. Purification using flash chromatography with DCM/MeOH (0–1.5% MeOH). Yield: 284 mg (73%) of **8** as a colorless oil. ^1^H NMR (400 MHz, CDCl_3_) δ 7.47–7.27 (m, 7H), 7.25–7.16 (m, 3H), 7.09–6.98 (m, 2H), 6.24–6.10 (m, 1H), 5.32–5.21 (m, 1H), 3.76 (s, 3H), 3.22 (s, 3H), 3.14–3.04 (m, 1H), 2.96–2.83 ppm (m, 1H). ESI‐MS [M + Na]^+^ = 349.3, HPLC t_*R*_ = 7.00 min.

### 4.31 ((*S*)‐*N*‐(3‐Oxo‐1‐phenylbutan‐2‐yl)benzo[b]thiophene‐2‐carboxamide (9)

The Weinreb amide **8** (184 mg, 1 eq.) was dissolved in Et_2_O (dry). The reaction mixture was cooled to −10°C in a salt‐ice bath, and 3 M methylmagnesium bromide in Et_2_O (3.2 eq) was added dropwise. After 5–6 h of stirring at 0°C, the reaction mixture was quenched with 1 N HCl and EtOAc (3 × 20 mL). The combined organic layers were dried over anhydrous Na_2_SO_4_, filtered, and concentrated under reduced pressure. Flash purification with PE/EtOAc (0–70% EtOAc). Yield: 99 mg (61%) of **9** as a white solid. ^1^H NMR (400 MHz, CDCl_3_) δ 7.87–7.81 (m, 2H), 7.73 (s, 1H), 7.46–7.37 (m, 2H), 7.33–7.30 (m, 1H), 7.29–7.27 (m, 1H), 7.26–7.23 (m, 1H), 7.20–7.16 (m, 2H), 6.84 (d, *J* = 6.8 Hz, 1H), 5.10–5.03 (m, 1H), 3.33–3.19 (m, 2H), 2.23 (s, 3H). ESI‐MS [M + Na]^+^ = 346.1, HPLC t_*R*_ = 8.13 min.

### 4.32 (*S*,*E*)‐*N*‐(5‐(Dimethylamino)‐3‐oxo‐1‐phenylpent‐4‐en‐2‐yl)benzo[b]thiophene‐2‐carboxamide (10)

A mixture of the ketone **9** (100 mg, 1 eq.) and *L*‐proline (4 mg, 0.1 eq) was dissolved in DMF (1 mL). *N*,*N*‐dimethylformamidedimethyl acetal (80 µL, 0.62 mmol) (2 eq) was added to the mixture and the reaction was stirred for 5h at 80°C. The mixture was cooled to room temperature after the reaction, and the solvent was removed to obtain the crude product. Flash purification with DCM/MeOH (0–1.5% MeOH). Yield: 103 mg (88%) of **10** as a white solid. ^1^H NMR (400 MHz, CDCl_3_) δ 7.76 (dd, *J* = 11.1, 7.7 Hz, 2H), 7.67 (s, 1H), 7.32 (m, 3H), 7.16 (m, 6H), 4.92 (s, 1H), 4.81 (d, *J* = 12.5 Hz, 1H), 3.14 (m, 2H), 2.99 (s, 3H), 2.66 (s, 3H). ^13^C NMR (101 MHz, CDCl_3_) δ 192.65, 161.60, 153.66, 141.12, 139.32, 139.13, 137.35, 129.93, 129.93, 128.30, 128.30, 126.70, 126.24, 125.15, 125.03, 124.87, 122.80, 45.16, 45.16, 39.57, 37.18. ESI‐MS [M + Na]^+^ = 401.1, HPLC t_*R*_ = 8.23 min.

### 4.33 General Procedure III for the synthesis of 1‐chloroacetyl iso‐indoline derivatives (13a‐f)

The respective amine (1.0 eq) was dissolved in dichloromethane (DCM) under an ice bath (0°C). Triethylamine (1.01 eq) was added to the solution, and the reaction mixture was stirred for 5 min at 0°C. A suspension of 2‐chloroacetyl chloride in DCM was then added dropwise over 10 min while maintaining the temperature at 0°C. The reaction mixture was stirred at 0–5°C for 30 min, then at room temperature for 24 h. After completion, the reaction mixture was quenched with water and extracted with DCM. The combined organic layers were dried over anhydrous Na_2_SO_4_, filtered, concentrated under reduced pressure, and purified by flash column chromatography.

### 4.34 2‐Chloro‐1‐(isoindolin‐2‐yl)ethan‐1‐one (13a)

According to general procedure III, *N*‐acylated isoindoline **13a** was synthesized reacting isoindoline (0.4 g, 3.36 mmol, 1 eq.), triethylamine (0.47 mL, 3.39 mmol, 1.01 eq.) and 2‐chloroacetyl chloride (0.27 mL, 3.36 mmol, 1 eq.) in DCM (5 mL) for 24 h at 0°C‐rt. Flash purification with petroleum ether/EtOAc (0–30% EtOAc). Yield: 0.4 g (67%) of **13a** as a yellow solid. ^1^H NMR (400 MHz, DMSO‐*d_6_*) δ 7.34 (m, 2H), 4.88 (s, 2H), 4.66 (d, *J* = 14.9 Hz, 2H), 4.46 (s, 4H); ^13^C NMR (101 MHz, DMSO‐*d6*) δ 164.54, 136.45, 135.69, 127.48, 127.48, 123.01, 122.76, 52.22, 51.41, 42.62. ESI‐MS [M + Na]^+^ = 218.8. HPLC t_*R*_ = 4.508 min.

### 4.35 1‐(4‐Bromoisoindolin‐2‐yl)‐2‐chloroethan‐1‐one (13b)

According to general procedure III, 4‐bromo isoindoline (0.25 g, 1.07 mmol, 1 eq), triethylamine (0.30 mL, 2.15 mmol, 2.02 eq), and 2‐chloroacetyl chloride (0.08 mL, 1.07 mmol, 1 eq) in DCM (5 mL) for 24 h at 0 ^o^C‐rt. Flash purification with petroleum ether/EtOAc (0–25% EtOAc). Yield: 0.16 g (53%) of **13b** as a beige solid. ^1^H NMR (400 MHz, DMSO‐*d*_6_) δ 7.52 (d, *J* = 7.9 Hz, 1H), 7.38 (t, *J* = 6.6 Hz, 1H), 7.28 (t, *J* = 7.8 Hz, 1H), 4.92 (d, *J* = 54.0 Hz, 2H), 4.70 (d, *J* = 68.2 Hz, 2H), 4.48 (d, *J* = 23.5 Hz, 2H). ^13^C NMR (101 MHz, DMSO‐*d*_6_) δ 164.71, 138.81, 138.18, 137.08, 136.30, 130.41, 130.37, 130.08, 130.04, 122.53, 122.31, 116.91, 116.52, 53.67, 53.43, 52.71, 52.47, 42.68, 42.54. ESI‐MS [M + Na]^+^ = 297.9. HPLC t_*R*_ = 6.272 min.

### 4.36 2‐Chloro‐1‐(4‐phenylisoindolin‐2‐yl)ethan‐1‐one (13c)

According to general procedure III, *N*‐acylated isoindoline **6c** was synthesized reacting 4‐phenyl isoindoline (0.165 g, 0.85 mmol, 1 eq), triethylamine (0.29 mL, 2.12 mmol, 2.50 eq), and 2‐chloroacetyl chloride (0.08 mL, 1.20 mmol, 1.02 eq) in DCM (5 mL) for 24h at 0 ^o^C‐rt. Flash purification with petroleum ether/EtOAc (0–25% EtOAc). Yield: 0.06 g (55%) of **13c** as a white solid. ^1^H NMR (400 MHz, DMSO‐*d*_6_) δ 7.48 (m, 4H), 7.39 (m, 4H), 4.97 (d, *J* = 9.3 Hz, 2H), 4.75 (d, *J* = 1.9 Hz, 2H), 4.46 (d, *J* = 1.8 Hz, 2H). ^13^C NMR (101 MHz, DMSO‐*d*_6_) δ 164.49, 139.36, 137.42, 136.85, 136.63, 134.11, 133.37, 128.79, 128.41, 127.92, 127.76, 127.65, 122.02, 52.27, 51.37, 42.61. ESI‐MS [M + Na]^+^ = 294. HPLC t_*R*_ = 7.545 min.

### 4.37 2‐Chloro‐1‐(5‐(5‐fluoro‐2‐(trifluoromethyl)pyridin‐3‐yl)isoindolin‐2‐yl)ethan‐1‐one (13d)

According to general procedure III, *N*‐acylated isoindoline **13d** was synthesized reacting 5‐(5‐fluoro‐2‐(trifluoromethyl)pyridin‐3‐yl)isoindoline (0.4 g, 3.36 mmol, 1 eq.), triethylamine (0.47 mL, 3.39 mmol, 1.01 eq), and 2‐chloroacetyl chloride (0.27 mL, 3.36 mmol, 1 eq) in DCM (5 mL) for 24 h at 0 ^o^C‐rt. Flash column chromatography with DCM/MeOH (0–80% ethyl acetate in petroleum ether). Yield: 109 mg (65%) of **13d** as a white solid. ^1^H NMR (400 MHz, DMSO‐*d*_6_) δ 8.83 (d, *J* = 2.7 Hz, 1H), 7.97 (dt, *J* = 9.0, 2.3 Hz, 1H), 7.50 (t, *J* = 7.3 Hz, 1H), 7.43 (d, *J* = 8.7 Hz, 1H), 7.35 (dd, *J* = 7.8, 1.7 Hz, 1H), 4.96 (d, *J* = 8.6 Hz, 2H), 4.76 (d, *J* = 10.8 Hz, 2H), 4.48 (s, 2H). ^13^C NMR (101 MHz, DMSO‐*d*_6_) δ 165.09, 161.51, 158.91, 137.80, 137.20, 136.47, 135.32, 128.58, 128.04, 127.83, 123.83, 123.44, 123.17, 52.60, 51.81, 43.10. ESI‐MS [M + Na]^+^ = 381.2, HPLC t_*R*_ = 6.5 min.

### 4.38 2‐Chloro‐1‐(4‐isobutoxyisoindolin‐2‐yl)ethan‐1‐one (13e)

According to general procedure III, *N*‐acylated isoindoline **13e** was synthesized by reacting 4‐isobutoxyisoindoline (0.13 g, 0.67 mmol, 1 eq.), triethylamine (0.189 mL, 1.36 mmol, 2 eq) and 2‐chloroacetyl chloride (0.06 mL, 0.815 mmol, 1.2 eq) in DCM (5 mL) for 24 h at 0°C‐rt. Flash purification with petroleum ether/EtOAc (0%–25% EtOAc). Yield: 0.11 g (67%) of **13e** as a white solid. ^1^H NMR (400 MHz, CDCl_3_) δ 7.24–7.12 (m, 1H), 6.81–6.71 (m, 1H), 6.68 (d, *J* = 8.2 Hz, 1H), 4.79 (t, *J* = 24.5 Hz, 4H), 4.08 (d, *J* = 16.9 Hz, 2H), 3.77–3.59 (m, 2H), 2.01 (m, *J* = 26.5, 13.3, 6.7 Hz, 1H), 0.95 (t, *J* = 6.9 Hz, 6H). ^13^C NMR (101 MHz, CDCl_3_) δ 165.31 (d, *J* = 6.7 Hz), 154.63 (d, *J* = 41.7 Hz), 137.51 (d, *J* = 23.9 Hz), 129.71 (d, *J* = 25.3 Hz), 124.25 (d, *J* = 35.8 Hz), 114.56 (d, *J* = 47.8 Hz), 109.87 (d, *J* = 34.1 Hz), 74.45 (s), 53.23 (d, *J* = 42.4 Hz), 50.89 (d, *J* = 60.8 Hz), 41.86 (s), 28.33 (d, *J* = 6.8 Hz), 19.33 (d, *J* = 7.8 Hz). ESI‐MS [M + H]^+^ = 268.3. HPLC t_*R*_ = 8.751 min.

### 4.39 2‐Chloro‐1‐(4‐(cyclohexylmethoxy)isoindolin‐2‐yl)ethan‐1‐one (13f)

According to general procedure III, *N*‐acylated isoindoline **13f** was synthesized reacting 4‐(cyclohexylmethoxy)isoindoline (0.215 g, 0.622 mmol, 1 eq), triethylamine (0.26 mL, 1.87 mmol, 3 eq), and 2‐chloroacetyl chloride (0.07 mL, 0.934 mmol, 1.5 eq) in DCM (5 mL) for 24 h at 0°C‐rt. Flash purification with petroleum ether/EtOAc (0–25% EtOAc). Yield: 0.13 g (67%) of **13f** as a white solid. ^1^H NMR (400 MHz, CDCl_3_) δ 7.19 (td, *J* = 7.6, 4.2 Hz, 1H), 6.77 (dd, *J* = 15.4, 7.6 Hz, 1H), 6.68 (d, *J* = 8.2 Hz, 1H), 4.91–4.66 (m, 4H), 4.07 (d, *J* = 19.2 Hz, 2H), 3.72 (dd, *J* = 7.5, 6.4 Hz, 2H), 1.84–1.51 (m, 6H), 1.31–1.06 (m, 3H), 0.97 (qd, *J* = 12.2, 3.0 Hz, 2H). ^13^C NMR (101 MHz, CDCl_3_) δ 165.29 (d, *J* = 5.8 Hz), 154.67 (d, *J* = 41.5 Hz), 137.47 (d, *J* = 24.1 Hz), 129.68 (d, *J* = 24.7 Hz), 124.24 (d, *J* = 37.0 Hz), 114.47 (d, *J* = 47.0 Hz), 109.85 (d, *J* = 34.5 Hz), 73.54 (s), 53.21 (d, *J* = 40.8 Hz), 50.90 (d, *J* = 59.1 Hz), 41.85 (d, *J* = 2.2 Hz), 37.71 (d, *J* = 15.9 Hz), 29.97 (s), 29.90 (s), 26.57 (d, *J* = 1.9 Hz), 25.90 (s), 25.86 (s). ESI‐MS [M + H]^+^ = 308.4 HPLC t_*R*_ = 9.497 min.

### 4.40 2‐Chloro‐1‐(1H‐indol‐2‐yl)ethan‐1‐one (15)

1‐(1H‐Indol‐2‐yl)ethan‐1‐one was suspended in dry THF, and benzyltrimethylammonium dichloroiodate, dissolved in THF (25 mL), was added dropwise under nitrogen atmosphere. The reaction mixture was stirred at room temperature for 72h, during which the color gradually changed from yellow to dark brown. After completion, the solvent was removed under reduced pressure. The residue was partitioned between diethyl ether (25 mL) and 5% aqueous Na_2_SO_4_ (40 mL) and stirred for 5 min. The organic layer was separated, dried over anhydrous Na_2_SO_4_, filtered, and concentrated under reduced pressure to afford the crude product. Flash purification with petroleum ether/EtOAc (0%–40% EtOAc). Yield: 0.56 g (92%) of **15** as a light brown solid. ^1^H NMR (400 MHz, DMSO) δ 11.94 (s, 1H), 7.72 (m, 1H), 7.47 (m, 2H), 7.31 (m, 1H), 7.10 (m, 1H), 5.06 (s, 2H); ^13^C NMR (101 MHz, DMSO) δ 184.21, 138.14, 132.73, 126.74, 126.03, 122.83, 120.56, 112.80, 110.40, 45.91; ESI‐MS [M + Na]^+^ = 217.8, HPLC t_*R*_ = 5.911 min.

### 4.41 PL^pro^ Inhibitory Assay

#### 4.41.1 Compound Testing Procedure A

Recombinant SARS‐CoV‐2 PL^pro^ was purchased from Acro Biosystems (PAE‐C5184) and diluted to 200 nM in assay buffer consisting of 50 mM HEPES pH 6.5, 150 mM NaCl, 0.01% Tween 20, and 0.1 mM dithiothreitol. Compounds for screening were diluted to 40 μM in the same assay buffer and incubated with an equal volume of PL^pro^ for 15 min at room temperature. In wells on a 384‐well plate, the enzyme/compound mixture was incubated with 100 μM of Z‐Arg‐Leu‐Arg‐Gly‐Gly‐AMC (Bachem, I1690) diluted in assay buffer for 1h at 37°C in a Biotek Synergy HTX plate reader, and readings were recorded at an excitation wavelength of 360 nm and an emission wavelength of 460 nm. The highest velocity of each reaction was monitored over 15 sequential readings and recorded as relative fluorescent units per sec. The velocities in each well were normalized to those in the DMSO controls. 10 μM of GRL0617 (MedChem Express) was used as a positive control. All assays were performed twice each in triplicate wells (*n* = 6), and the final concentrations of enzyme and substrate in each well were 50 nM and 50 μM, respectively.

*Dose response assay* **.** Dose‐response assays were performed with the most potent compound. Compounds stored as 10 mM stocks in DMSO were twofold serially diluted in DMSO 8 times, yielding 78.13 μM. From here, compounds were further diluted 1 in 250 in assay buffer and then incubated with an equal volume of 20 nM of SARS‐CoV‐2 PL^pro^ for 30 min at room temperature. The reaction was started by the addition of an equal volume of 50 μM of Z‐Arg‐Leu‐Arg‐Gly‐Gly‐AMC. All assays were performed twice, each in triplicate wells (*n* = 6), and the final concentrations of enzyme and substrate in each well were 20 nM and 50 μM, respectively. The final inhibitor's concentration ranged from 10 μM to 0.078 nM. The velocity of each reaction was normalized to the DMSO control. Dose‐response curves were generated using GraphPad Prism 8.2.1.

#### 4.41.2 Compound Testing Procedure B

PL^pro^ from SARS‐CoV‐2 was produced and purified as previously described [30, 38]. The inhibition assay used the FRET‐peptide substrate Abz‐TLKGG↓APIKEDDPS‐EDDnp at a final concentration of 20 μM. For each test, 70 nM of PL^pro^ enzyme was standardized, and potential inhibitory compounds were initially evaluated at 100 μM. Reactions were carried out in a buffer containing 50 mM HEPES (pH 7.5), 5 mM DTT, and 0.01% Triton, and each inhibitor candidate was incubated at 37°C for 30 min. Following incubation, the substrate was introduced, and enzyme activity was measured by monitoring fluorescence (excitation/emission: 320/420 nm) every 30 s for 30 min using a SpectraMax Gemini EM microplate reader. Compounds yielding over 50% inhibition relative to native enzyme controls were selected for further IC_50_ analysis. For these assays, samples were pre‐incubated with serially diluted inhibitors (ranging from 1000 to 0.24 μM in twofold increments) at 37°C for 30 min. Inhibitors were diluted in DMSO, with controls containing equivalent DMSO volumes without inhibitors, and both were performed with and without protein. Fluorescence readings were again taken every 30 sec for 30 min at 37°C, using the same detection settings. All experiments were run in triplicate, and the initial rates were determined from the linear segment of each reaction curve. Data, including inhibition curves and IC_50_ values, were analyzed and presented with GraphPad Prism 8.4.3.

### 4.42 Cytotoxicity and Antiviral Assay

#### 4.42.1 Cells and Viruses

Calu‐3 (human lung, ATCC Cat# HTB‐55) and Vero E6 (African green monkey kidney, ATCC Cat# CRL‐1586) cells were maintained in DMEM supplemented with 10% fetal bovine serum (FBS). SARS‐CoV‐2 isolate NK, Pango lineage B.1.513 was propagated in Calu‐3 cell and virus titers of stocks were determined by titration on Vero E6 cells.

#### 4.42.2 Cell Vitality Assay

Cell vitality was determined using the CellTiter‐Glo kit according to the manufacturer´s protocol. Briefly, Calu‐3 and Vero E6 cells were grown in 96‐well plates until approximately 80% confluence, and then incubated with DMSO (solvent control), 10, or 1 µM of the PL^pro^ inhibitor **2t**. After 24h, cell culture supernatants were removed, and 50 µl of CellTiter‐Glo substrate was added to each well and incubated for 30 min. Cell lysates were transferred into white 96‐well plates, and luminescence was measured using a Hidex Sense plate luminometer (Hidex).

#### 4.42.3 Antiviral Activity Against SARS‐CoV‐2

All work with infectious SARS‐CoV‐2 was conducted under BSL‐3 conditions at the German Primate Centre, Göttingen/Germany. Calu‐3 cells grown in 48‐well plates were incubated with DMSO (solvent control) or 10, 7, 5, 3, and 2 µM of inhibitor 2t for 1 h at 37°C prior to infection. The inhibitor‐containing cell culture medium was removed, and cells were inoculated with the SARS‐CoV‐2 isolate NK (Pango lineage B.1.513) at an MOI of 0.01 for 1 h at 37°C. Next, the virus inoculum was removed, the cells were washed with PBS three times, and the cells were further incubated in DMEM supplemented with 10% FBS containing the respective inhibitor. At 24 h post‐infection (p.i.), supernatants were harvested and viral titers were determined as plaque‐forming units/mL (PFU/mL) by titration on Vero E6 cells.

### 4.43 Computational Studies

#### 4.43.1 Protein Structure Preparation and Molecular Docking

System preparation and docking calculations were performed using the Schrödinger Drug Discovery suite for molecular modeling (version 2024.3). The SARS‐CoV‐2 PL^pro^ structure interacting with GRL0617 (PDB: 7JIR, 2.09 Å) [21] was selected based on the structure's quality and existing ligand. Mutations were changed back to the wild‐type sequence. The protein–ligand complex was prepared with the Protein Preparation Wizard to fix protonation states of amino acids, add hydrogens, and fix missing side‐chain atoms, selecting the most likely ionization state as proposed by the software. Finally, each structure was globally minimized using the steepest descent method (cut‐off: 0.5 Å for all atoms). All ligands for docking were drawn in Maestro and prepared in LigPrep [45] to generate 3D conformations, adjust the protonation state to physiological pH (7.4), and calculate partial atomic charges using the OPLS4 force field [46]. Docking studies with the prepared ligands were performed using Glide (Glide V7.7) [47, 48]. With the flexible modality of induced‐fit docking with standard precision, followed by a side‐chain minimization step using Prime. Ligands were docked within a grid around 12 Å from the centroid of the co‐crystallized ligand's binding site.

#### 4.43.2 Molecular Dynamics Simulations

The simulation protocol was inspired by Ferreira et al. [19]. MD simulations were carried out using Desmond with the OPLS4 force‐field [46]. The simulated system encompassed the protein‐ligand complexes, a predefined water model (TIP3P [49]) as a solvent, and counterions. The system was treated in a cubic box with periodic boundary conditions, with box dimensions specified by a 10 Å distance from the box edges to any protein atom. In all simulations, we used a time step of 1 fs; short‐range Coulombic interactions were treated with a cutoff of 9.0 Å using the short‐range method, while the Smooth Particle Mesh Ewald (PME) method handled long‐range Coulombic interactions [50]. Initially, the system's relaxation was performed using Steepest Descent and the limited‐memory Broyden‐Fletcher‐Goldfarb‐Shanno algorithms in a hybrid manner, according to the established protocol available in the Desmond standard settings. During the equilibration step, the simulation was performed under the NPT ensemble for 5 ns, implementing the Berendsen thermostat and barostat methods [51]. A constant temperature of 310 K was maintained throughout the simulation using the Nose‐Hoover thermostat algorithm 9 and the Martyna‐Tobias‐Klein barostat10 to maintain 1 atm of pressure. After minimization and relaxation of the system, we continued with the production step for at least 200 ns, recording frames every 1000 ps. Five independent replicas were produced for each compound, resulting in a total of ~1,000 ns simulation/ligand. Trajectories and interaction data are available on the Zenodo repository [52] (access numbers 10.5281/zenodo.17880846). The representative structures were selected by inspecting changes in the Root‐mean‐square deviation (RMSD; see Supporting Information of Figure S63). For the figures, a representative frame was chosen at random from points along the trajectory where the RMSD was not fluctuating, after equilibration. Those simulations are long enough to evaluate protein‐ligand binding, and five replicas are indeed sufficient by the ACS guidelines for MD simulation reports (which recommend at least 3 independent replicas) [53]. Even though some of the ligand's RMSD values do change between replicas, overall, the ligand's RMSD remains relatively stable (<3 Å for most replicas).

#### 4.43.3 Protein‐Ligand Interactions and Protein/Ligand Properties

The Maestro simulation interaction analysis tool (Schrödinger, LLC) was used to analyze RMSD, RMSF, and interactions. We used the default interaction values for H‐bonds: a cut‐off of 2.5 Å between donor and acceptor atoms, a donor angle of 120°, and an acceptor angle of 90°. Hydrophobic interactions: a cut‐off of 3.6 Å between ligand's aromatic or aliphatic carbons and a hydrophobic side chain; π–π interactions were defined as two aromatic groups stacked face‐to‐face or face‐to‐edge. Water bridge interactions: default cut‐off of 2.8 Å for donor and acceptor atoms, donor angle of 110°, and acceptor angle of 90°. For angle and distance calculations, the Maestro event analysis tool (Schrödinger, LLC) was used.

#### 4.43.4 MM‐GBSA Binding Energy Calculations

Molecular mechanics with generalized Born and surface area (MM‐GBSA) predicts the binding free energy of protein‐ligand complexes, and the ranking of ligands based on the free energy could be correlated to the experimental binding affinities, especially in a congeneric series. Every 10th frame of the simulations was considered for the calculations. These were used as input files for the MM‐GBSA calculations with the thermal_mmgbsa.py script from the Schrödinger package, using Prime [54]. Calculated free‐binding energies are represented by the MM/GBSA and normalized by the number of heavy atoms (HAC), according to the following formula: ligand efficiency (or LN) = (Binding energy) / (1 + ln(HAC)) and is expressed in kcal/mol.HAC, where HAC is the Heavy Atom Count.

#### 4.43.5 Visualization and Plotting

Structural data visualization was conducted with PyMOL v.3.1 (Schrödinger LLC, New York, NY, USA). Data visualization was also completed using GraphPad Prism (v.10.1 for Windows, GraphPad Software, San Diego, CA, USA).

## Author Contributions

**Elena‐Oriana Iuga**, **Nilu Gone**: design of the study, data curation, formal analysis, investigation, methodology (organic synthesis, LCMS, HPLC, and NMR experiments), writing – original draft. **Giovanna de Jesus Agostinetto, Liam Urich, Anthony J. O’Donoghue**: data curation, Biochemical assays. **Mariana Ortiz de Godoy**: Biochemical assays. **Nadine Krüger**: Cytotoxicity and antiviral studies. **Gabriel Correa Verissimo** and **Thales Kronenberger** performed docking and molecular stimulation studies. **Rafael Victorio Carvalho Guido**, data curation, and supervision. **Stefan A. Laufer** and **Thanigaimalai Pillaiyar**: Conceptualization, data curation, formal analysis, acquisition, project administration, resources, supervision, validation, writing – contribution to original draft and review and editing. All authors edited or approved the submitted manuscript.

## Supporting Information

Additional supporting information can be found online in the Supporting Information section.

## Conflicts of Interest

The authors declare no conflicts of interest.

## Supporting information

Supplementary Material

## Data Availability

The data that support the findings of this study are available from the corresponding author upon reasonable request.
