# Supplementary material for: Inhibitors Targeting SARS‐CoV‐2 Papain‐Like Protease: Screening, Design, Synthesis, and Biological Evaluation
Source: ChemMedChem. 2026 May 8;21(9):e202600007. doi: 10.1002/cmdc.202600007 (PMC13156432; doi:10.1002/cmdc.202600007)
Supplement: Supplementary file 1 — Supplementary Material [file CMDC-21-e202600007-s001.pdf]

SUPPORTING INFORMATION

Inhibitors Targeting SARS-CoV-2 Papain-Like Protease: Screening, Design, Synthesis, and Biological Evaluation

Elena-Oriana Iuga[a,b], Nilu Gone[a,b], Mariana Ortiz de Godoy[c], Gabriel Correa Verissimo[d], Giovanna de Jesus Agostinetto[e,f], Liam Urich[f], Nadine Krüger[g], Thales Kronenberger[h,i], Rafael Victorio Carvalho Guido[c], Anthony O'Donoghue[f], Stefan A. Laufer[a.b], and Thanigaimalai Pillaiyar[a,b*]

[a] Institute of Pharmacy, Pharmaceutical/Medicinal Chemistry, Eberhard Karls University, 72076 Tübingen, Germany

[b] Tübingen Center for Academic Drug Discovery (TüCAD2), Eberhard Karls University, 72076 Tübingen, Germany

[c] São Carlos Institute of Physics, University of São Paulo (USP), 13566-590 São Carlos, SP, Brazil

[d] Faculdade de Farmácia, Universidade Federal de Minas Gerais (UFMG), Belo Horizonte, MG, 31270-901, Brazil

[e] Faculdade de Farmácia, Universidade Federal do Rio Grande do Sul, Porto Alegre, Brazil[

[f] Skaggs School of Pharmacy and Pharmaceutical Sciences, University of California San Diego, La Jolla, USA

[g] Platform Infection Models, German Primate Center, Leibniz Institute for Primate Research Göttingen, Kellnerweg 4, 37077 Göttingen, Germany

[h] School of Pharmacy, Faculty of Health Sciences, University of Eastern Finland, Kuopio 70211, Finland.

[i] German Center for Infection Research (DZIF), partner-site Tübingen. Institute of Medical Microbiology and Hygiene, Interfaculty Institute of Microbiology and Infection Medicine (IMIT), University of Tübingen, Elfriede-Aulhorn-Str. 6, 72076 Tübingen, Germany.

[i] Cluster of Excellence "Image Guided and Functionally Instructed Tumor Therapies" (iFIT), Eberhard Karls University of Tuebingen, Tuebingen 72076, Germany.

*E-Mail: thanigaimalai.pillaiyar@uni-tuebingen.de

Content

1.	Figure S1: Molecular dynamics simulation of a potent inhibitor with papain-like protease	S3
2	Figure S2-S65; NMR spectra	S4-35
3.	HPLC data of targeted prepared compounds	S36-51
4.	Mass spectra of targeted prepared compounds	S52-56

1. Molecular Dynamics Simulation of a Potent Inhibitor with Papin-like Protease

The representative structures were selected by inspecting changes in the Root-mean-square deviation (RMSD; Figure S63). For the figures, a representative frame was selected at random from points along the trajectory where the RMSD was not fluctuating, after equilibration. Those simulations are long enough to evaluate protein-ligand binding, and 5 replicas are indeed sufficient by the ACS guidelines for MD simulation reports (which recommend at least 3 independent replicas 12). Even though some of the ligand's RMSD values do change between replicas, overall, the ligand's RMSD remains relatively stable (<3Å for most replicas).

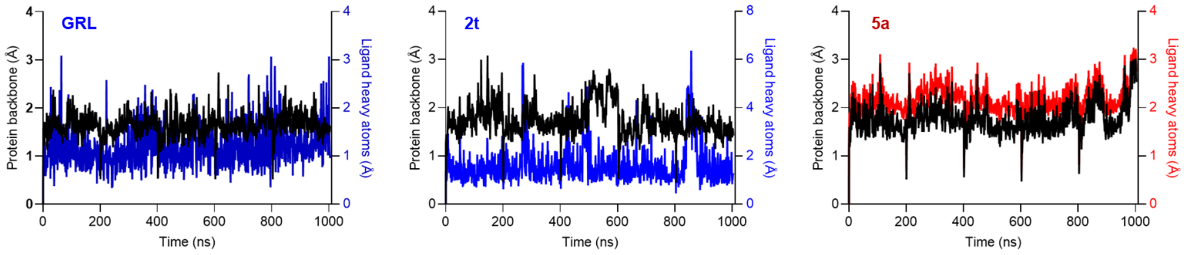

Figure S1. Root mean square deviation (RMSD) for protein backbone (black lines) and ligand's heavy atoms (colored lines) determined along the five simulation replicas.

2. NMR spectra

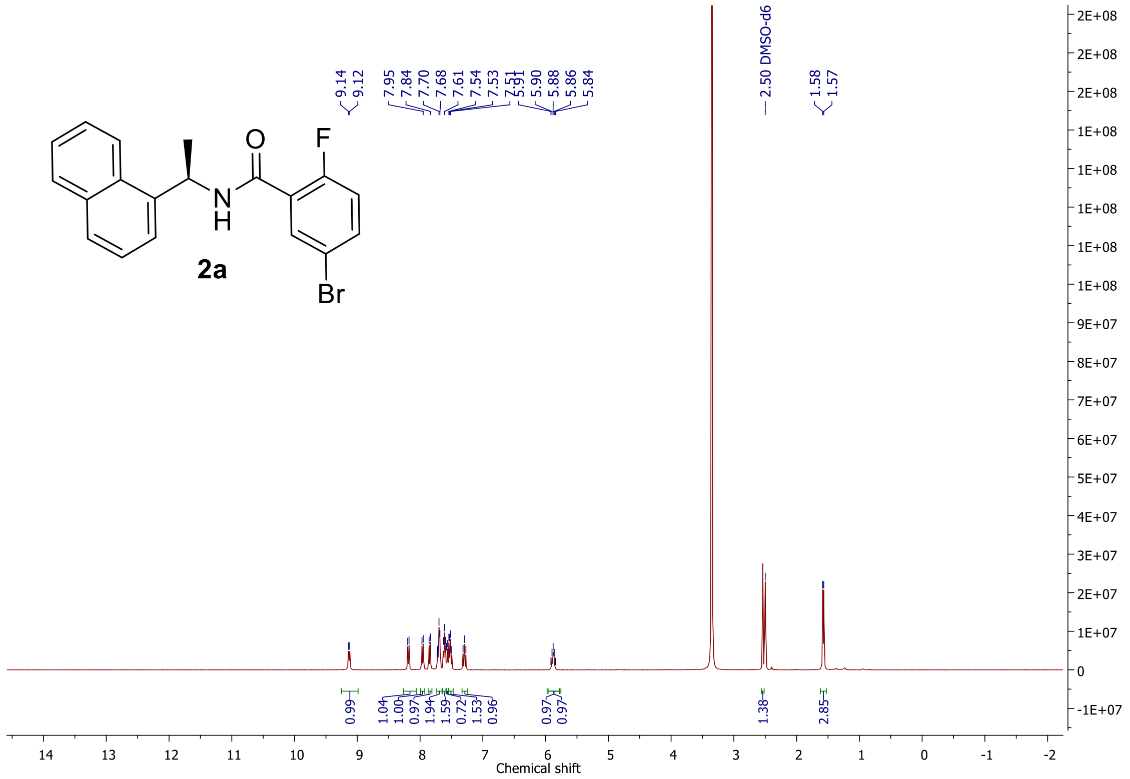

Figure S2. ^{1}H NMR spectrum (DMSO-*d6*, 400 MHz) of (R)-5-bromo-2-fluoro-N-(1-(naphthalen-1-yl)ethyl)benzamide **2a**

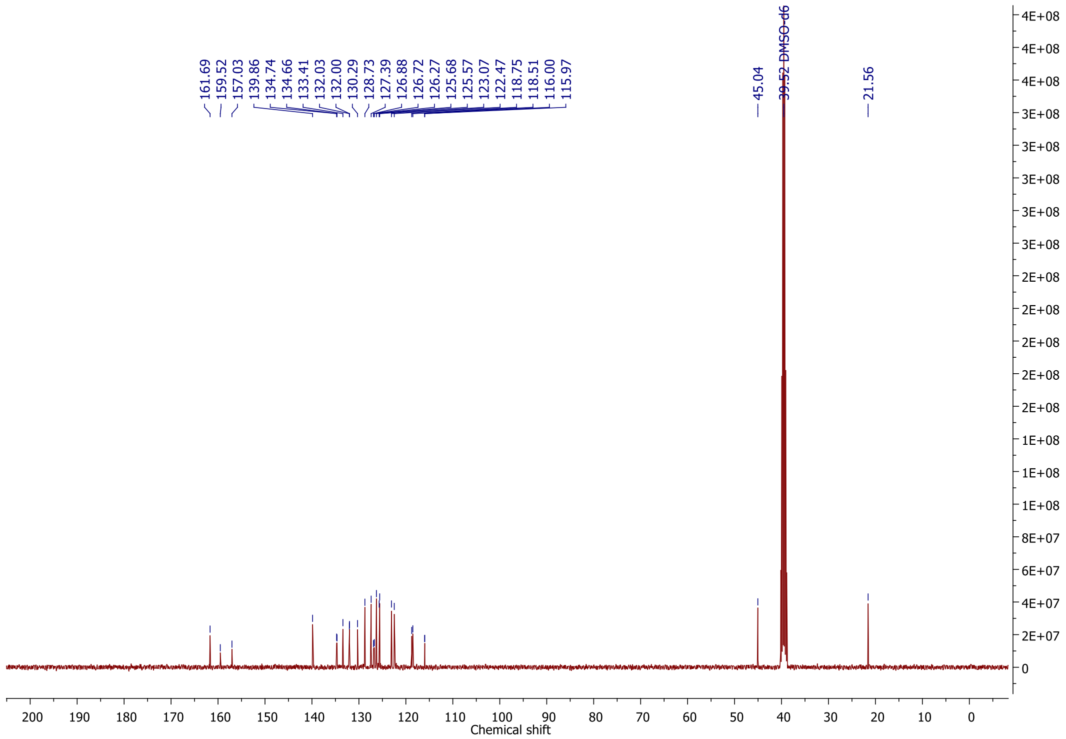

Figure S3. ^{13}C NMR spectrum (DMSO-*d6*, 101 MHz) of (R)-5-bromo-2-fluoro-N-(1-(naphthalen-1-yl)ethyl)benzamide **2a**

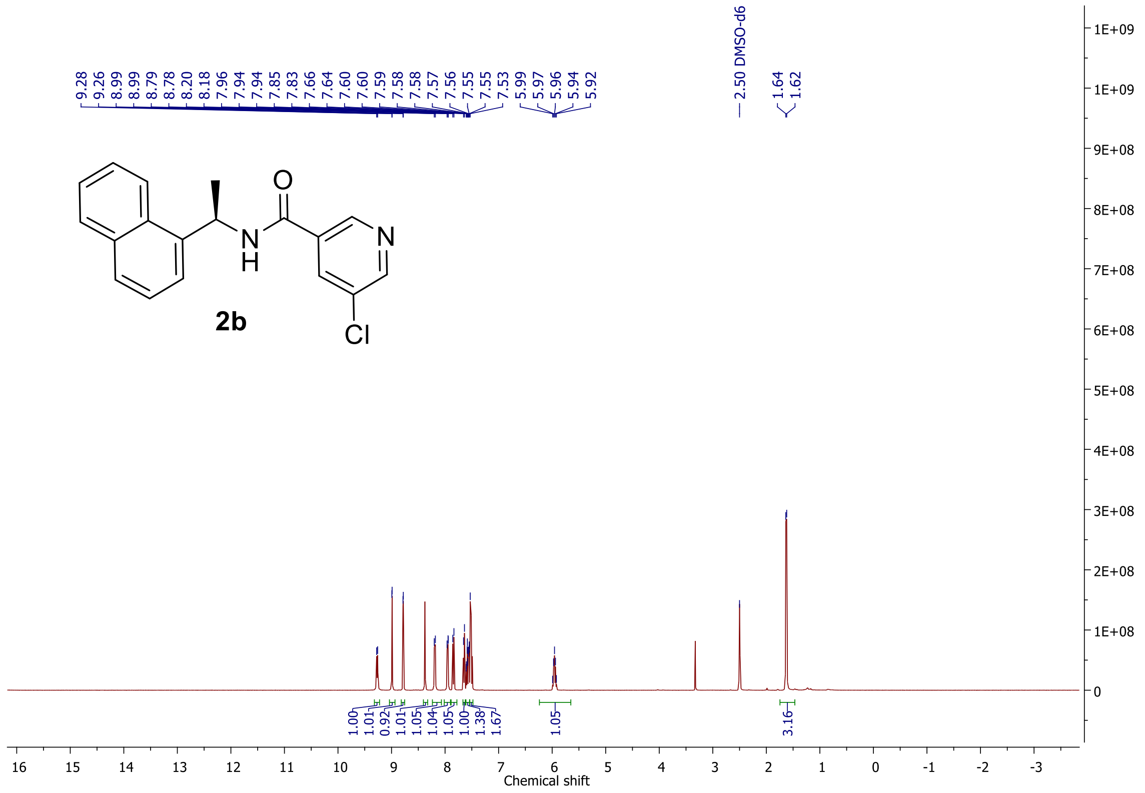

Figure S4. ^{1}H NMR spectrum (DMSO-*d6*, 400 MHz) of (R)-5-chloro-N-(1-(naphthalen-1-yl)ethyl)nicotinamide **2b**

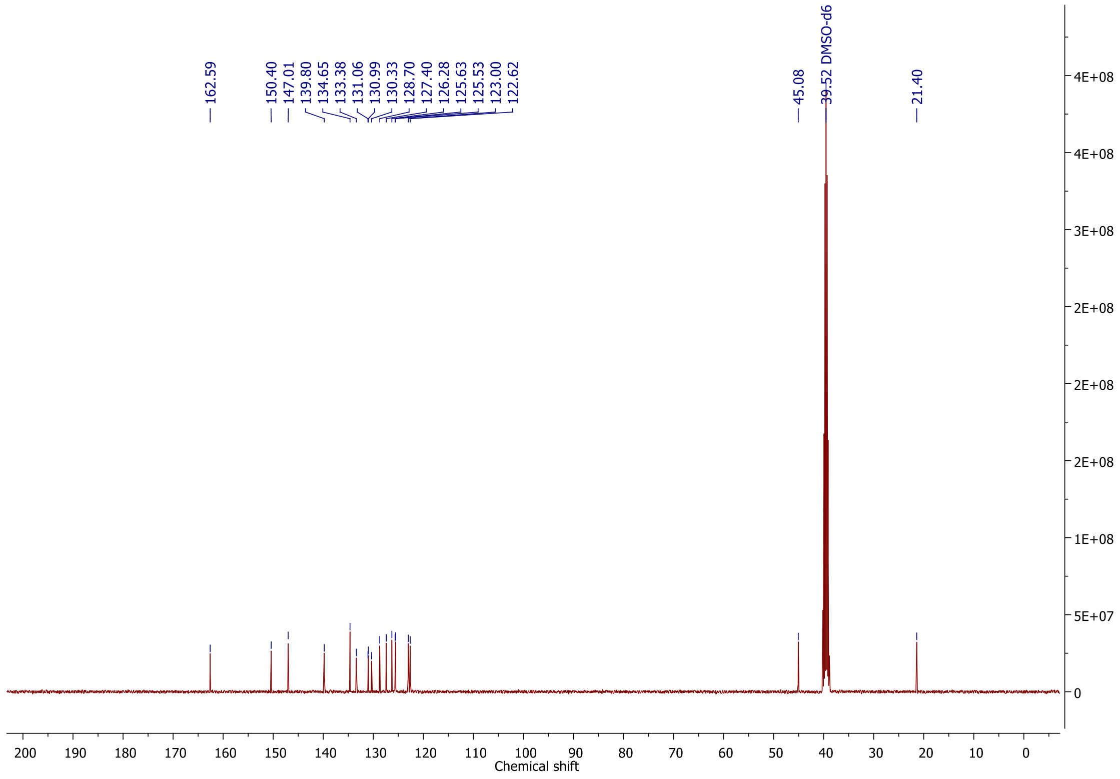

Figure S5. ^{13}C NMR spectrum (DMSO-*d6*, 101 MHz) of (R)-5-chloro-N-(1-(naphthalen-1-yl)ethyl)nicotinamide **2b**

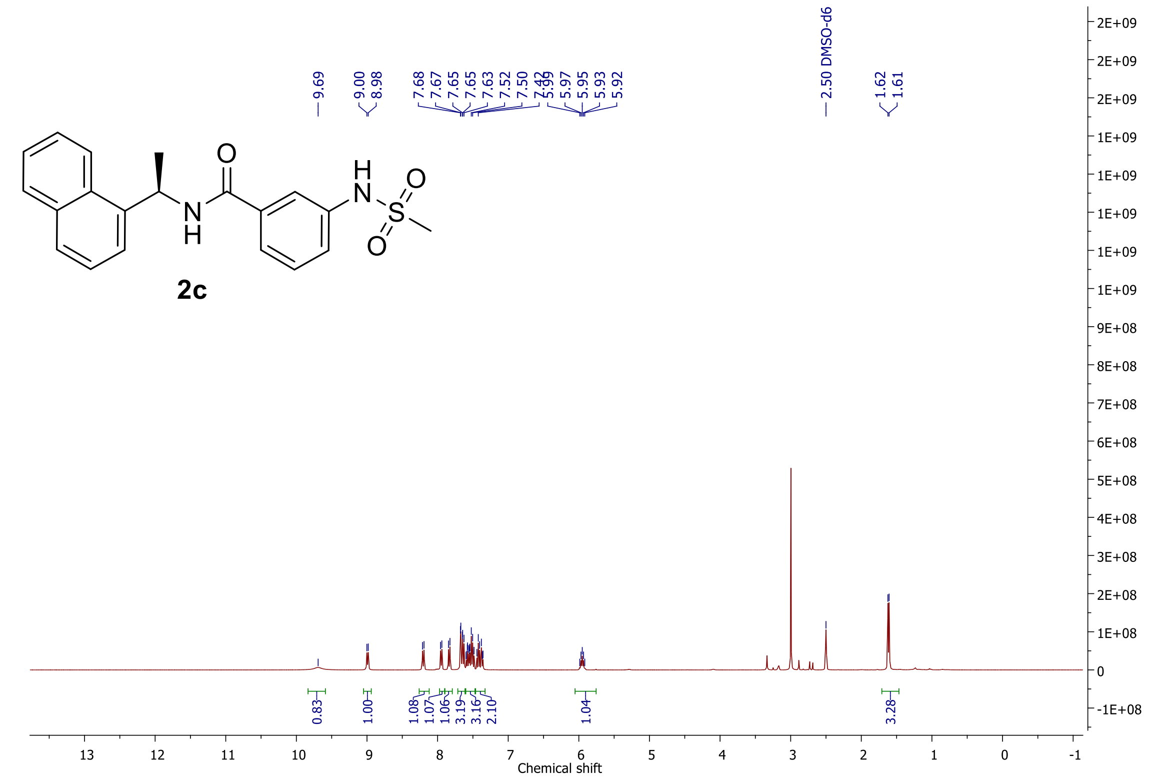

Figure S6. ^{1}H NMR spectrum (DMSO-*d6*, 400 MHz) of (R)-3-(methylsulfonamido)-N-(1-(naphthalen-1-yl)ethyl)benzamide **2c**

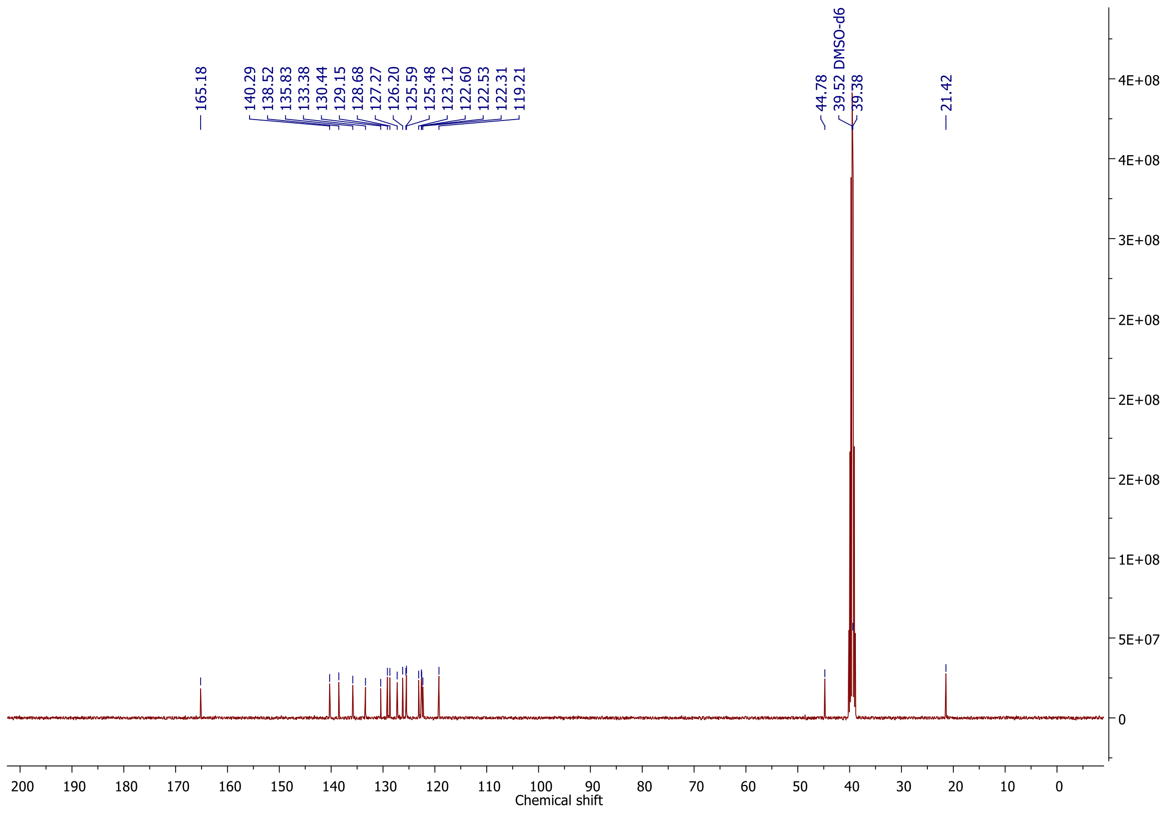

Figure S7. ^{13}C NMR spectrum (DMSO-*d6*, 101 MHz) of **(**R)-3-(methylsulfonamido)-N-(1-(naphthalen-1-yl)ethyl)benzamide **2c**

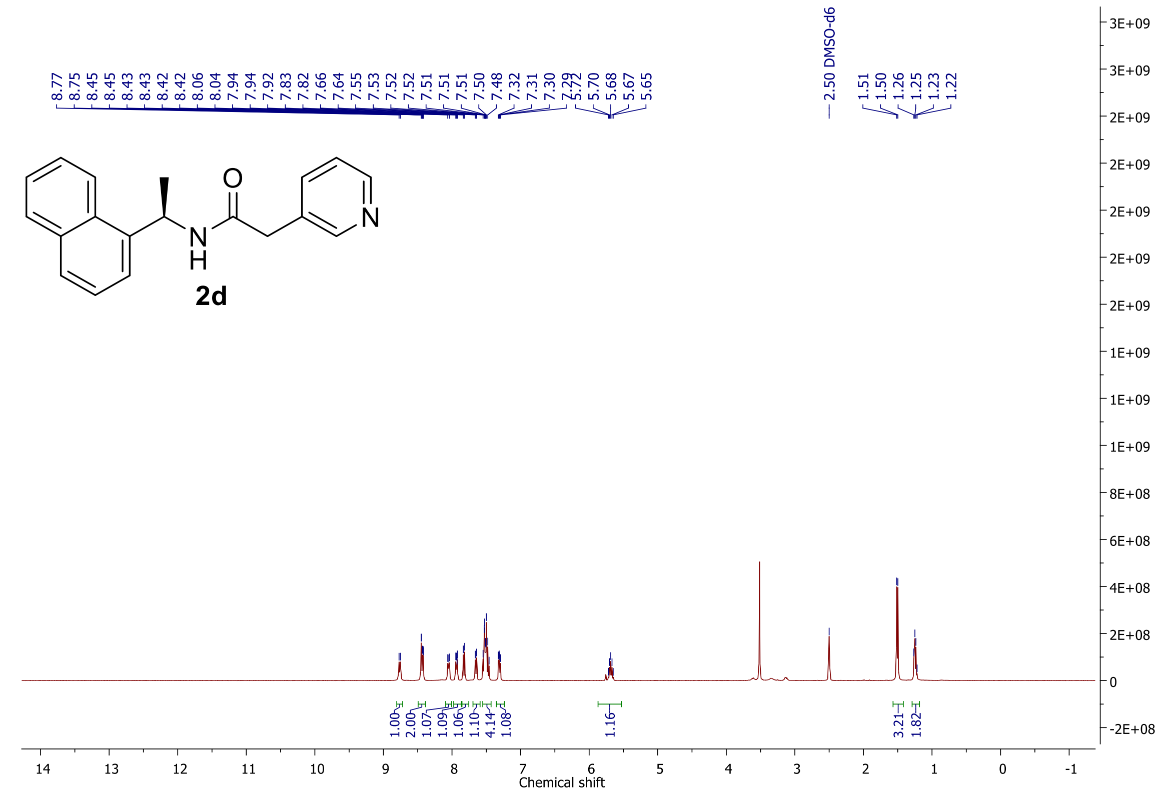

Figure S8. ^{1}H NMR spectrum (DMSO-*d6*, 400 MHz) of (R)-N-(1-(naphthalen-1-yl)ethyl)-2-(pyridin-3-yl)acetamide **2d**

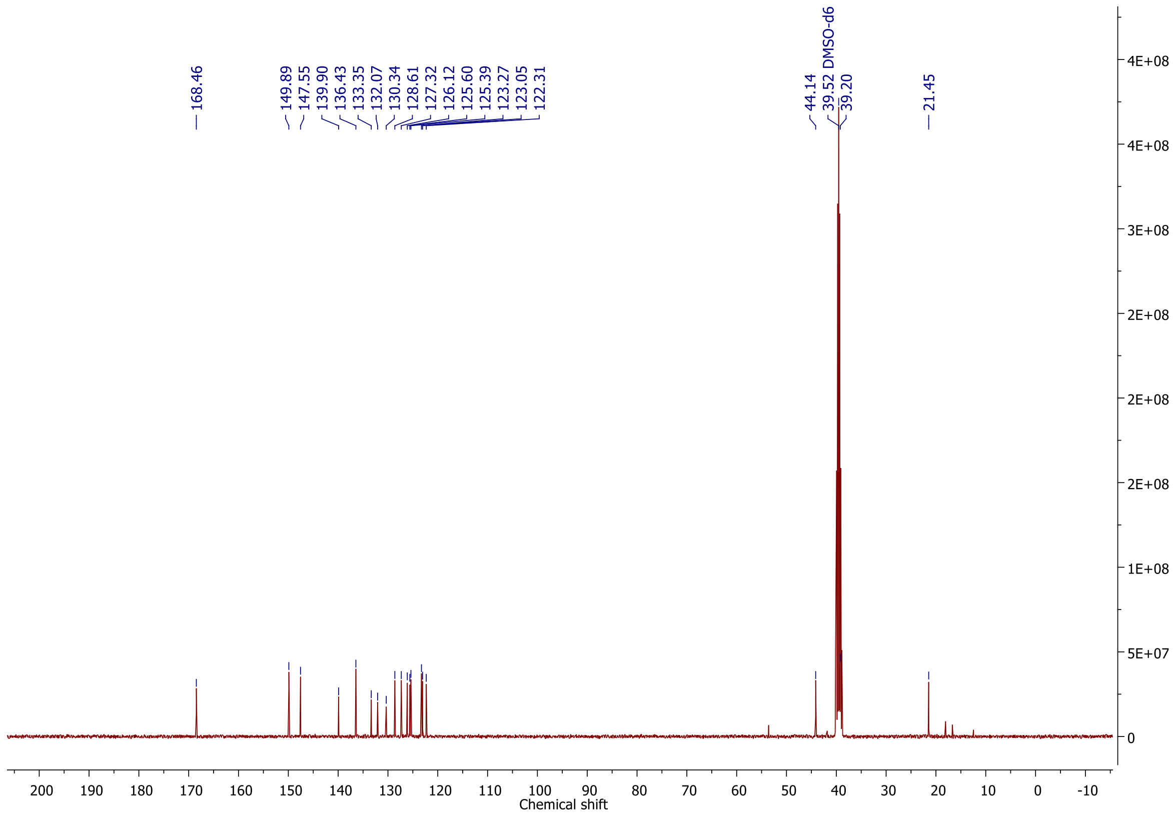

Figure S9. ^{13}C NMR spectrum (DMSO-*d6*, 101 MHz) of (R)-N-(1-(naphthalen-1-yl)ethyl)-2-(pyridin-3-yl)acetamide **2d**

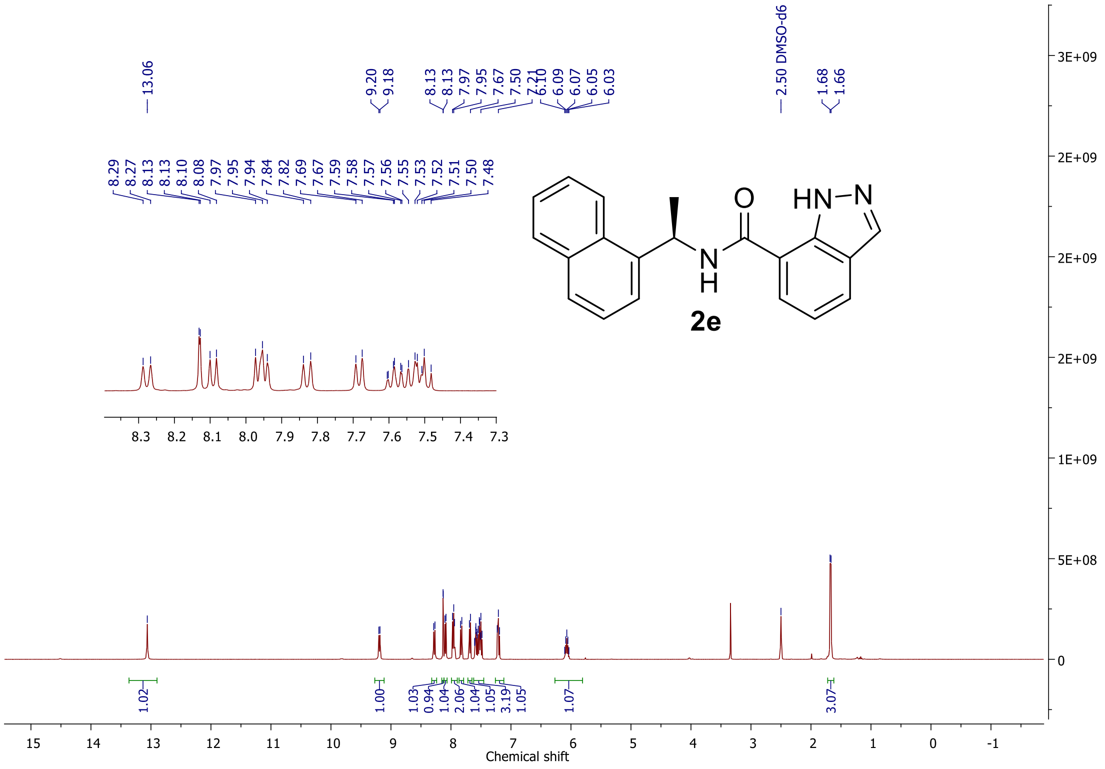

Figure S10. ^{1}H NMR spectrum (DMSO-*d6*, 400 MHz) of (S)-n-(naphthalen-1-yl)ethyl)-1-H-indazole-7-carboxamide **2e**

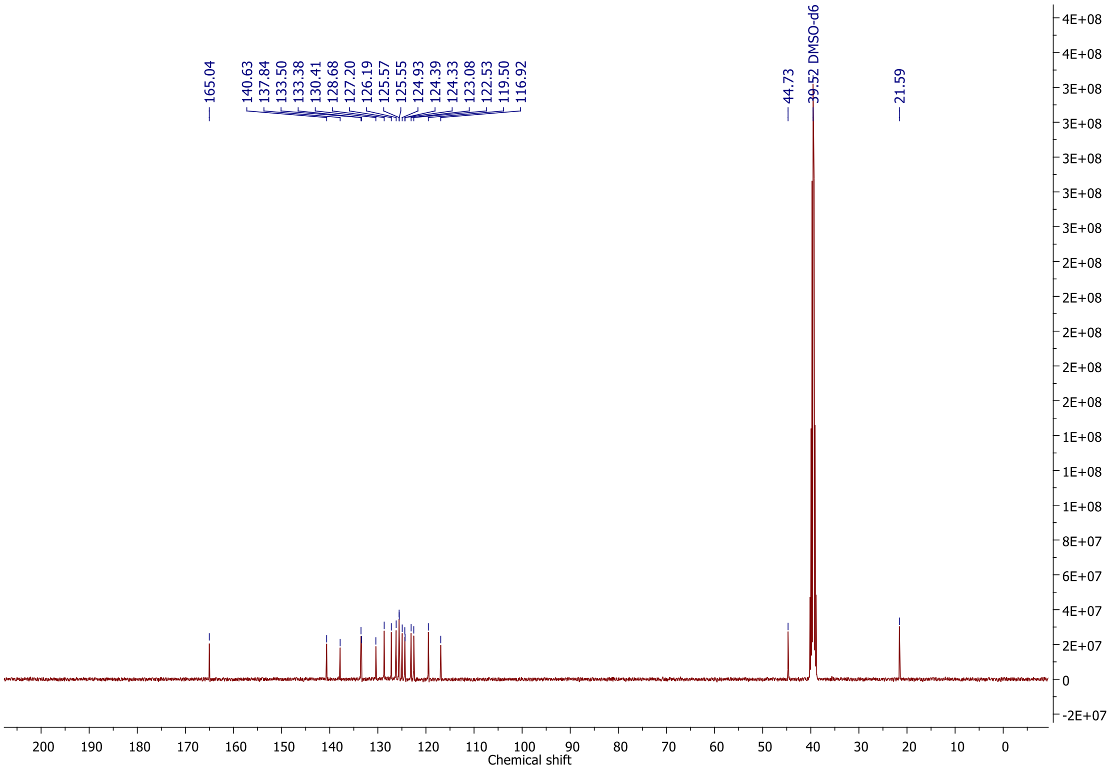

Figure S11. ^{13}C NMR spectrum (DMSO-*d6*, 101 MHz) of (S)-n-(naphthalen-1-yl)ethyl)-1-H-indazole-7-carboxamide **2e**

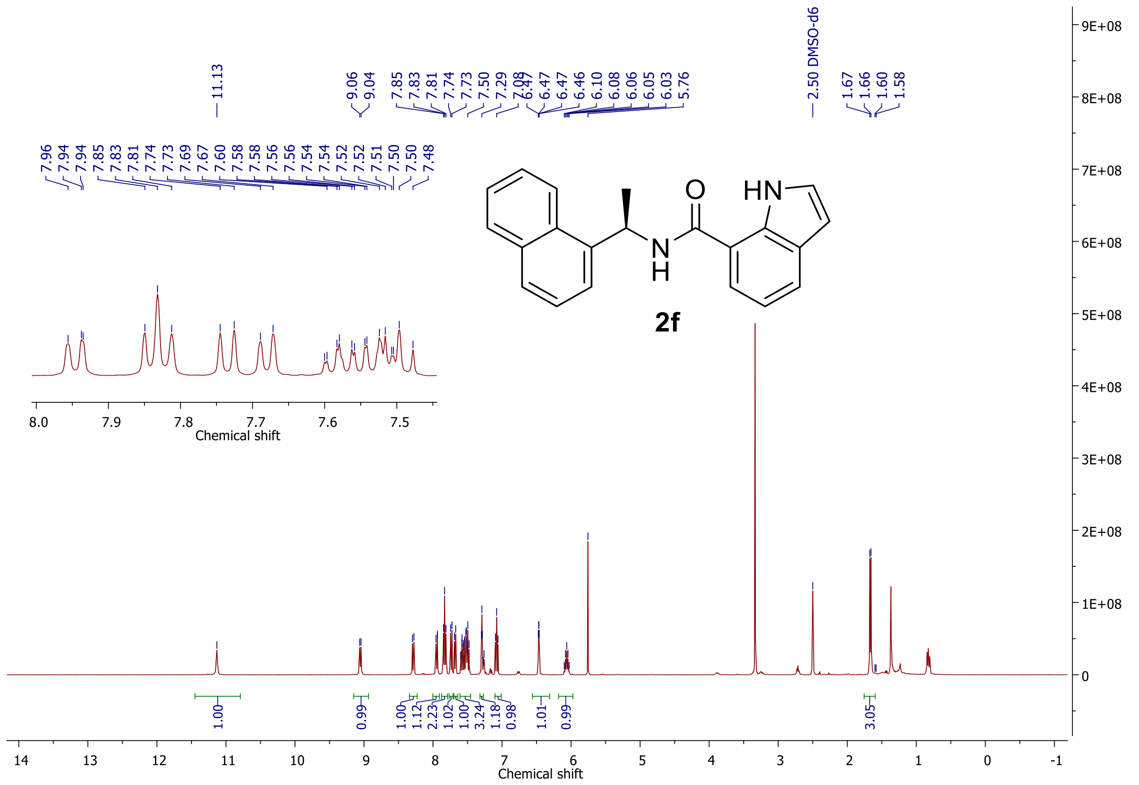

Figure S12. ^{1}H NMR spectrum (DMSO-*d6*, 400 MHz) of (R)-N-(1-(naphthalen-1-yl)ethyl)-1H-indole-7-carboxamide **2f**

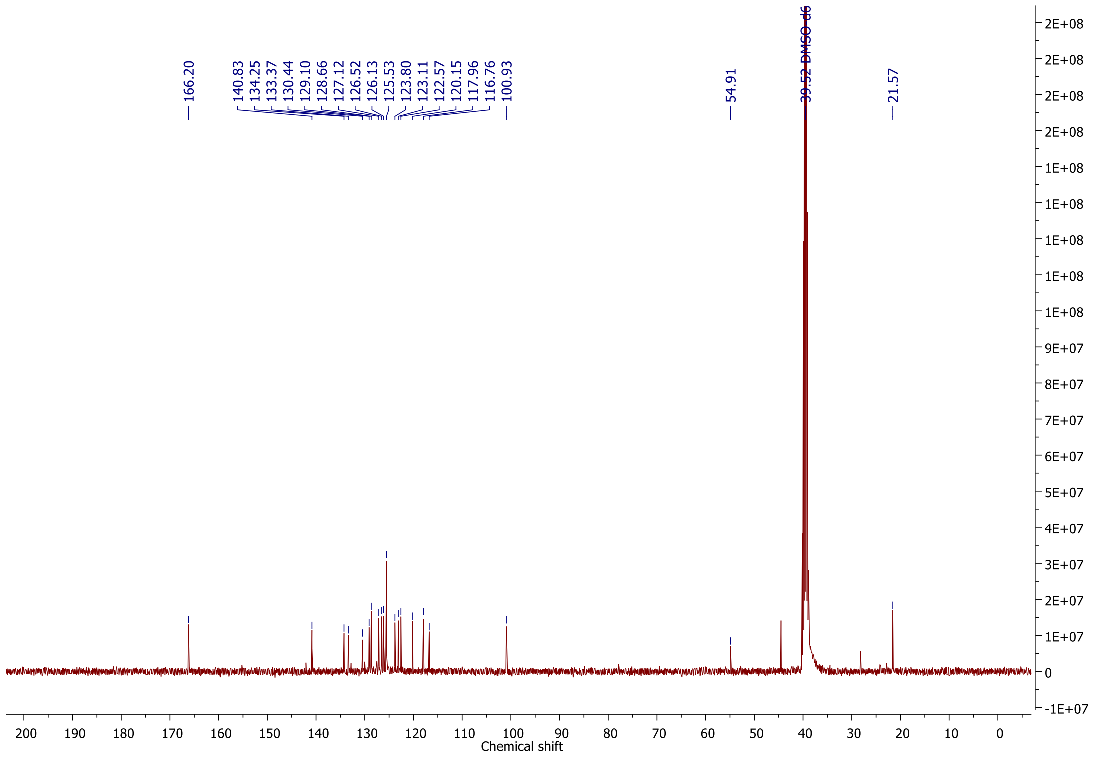

Figure S13. ^{13}C NMR spectrum (DMSO-*d6*, 101 MHz) of (R)-N-(1-(naphthalen-1-yl)ethyl)-1H-indole-7-carboxamide **2f**

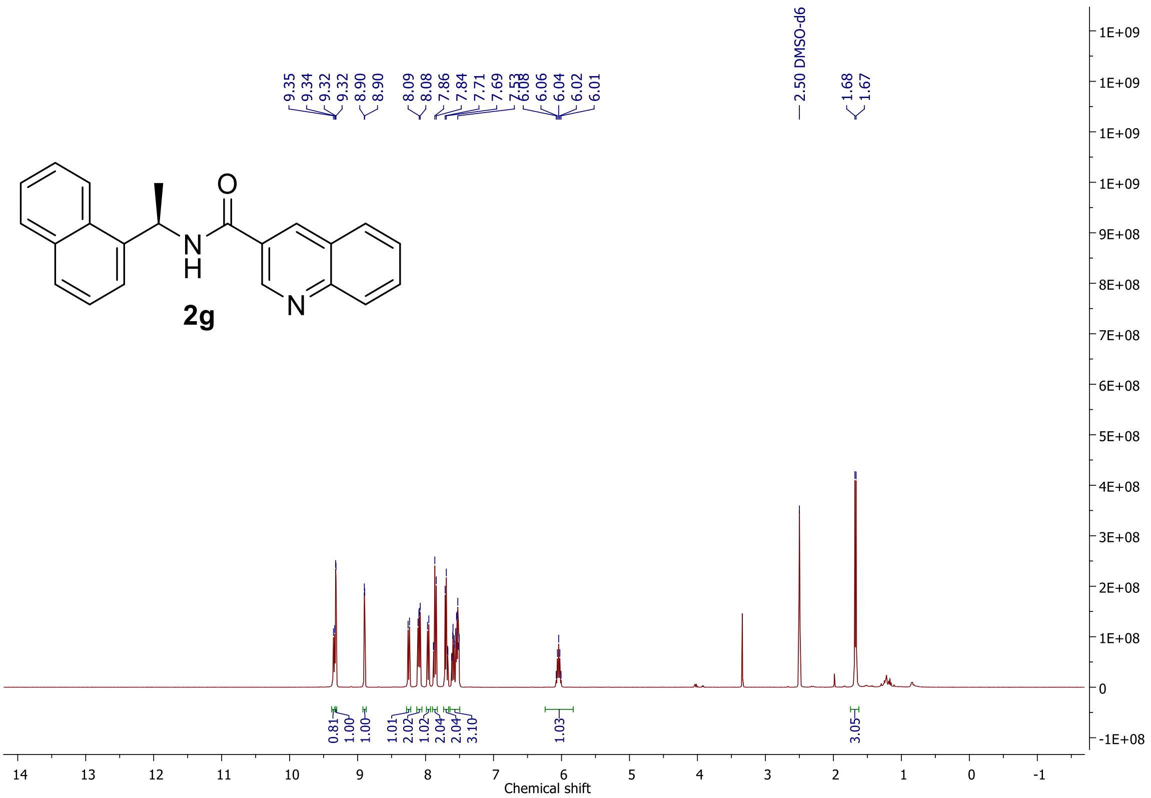

Figure S14. ^{1}H NMR spectrum (DMSO-*d6*, 400 MHz) of (R)-N-(1-(naphthalen-1-yl)ethyl)quinoline-3-carboxamide **2g**

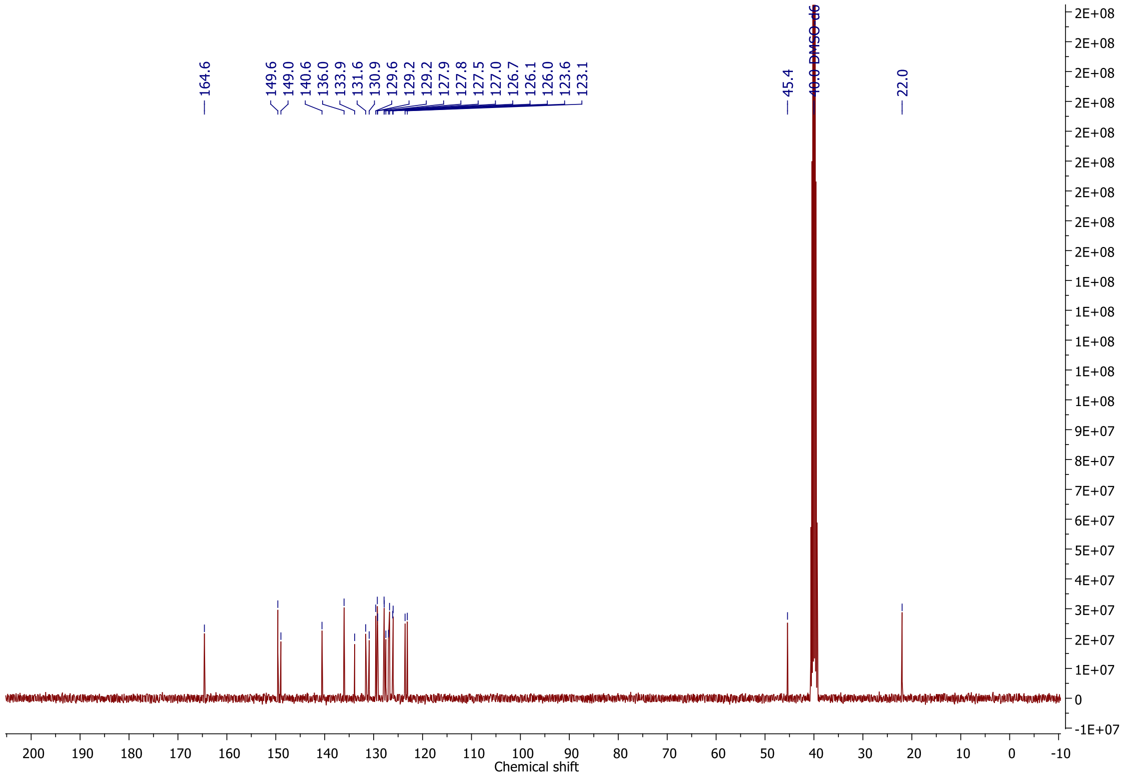

Figure S15. ^{13}C NMR spectrum (DMSO-*d6*, 101 MHz) of (R)-N-(1-(naphthalen-1-yl)ethyl)quinoline-3-carboxamide **2g**

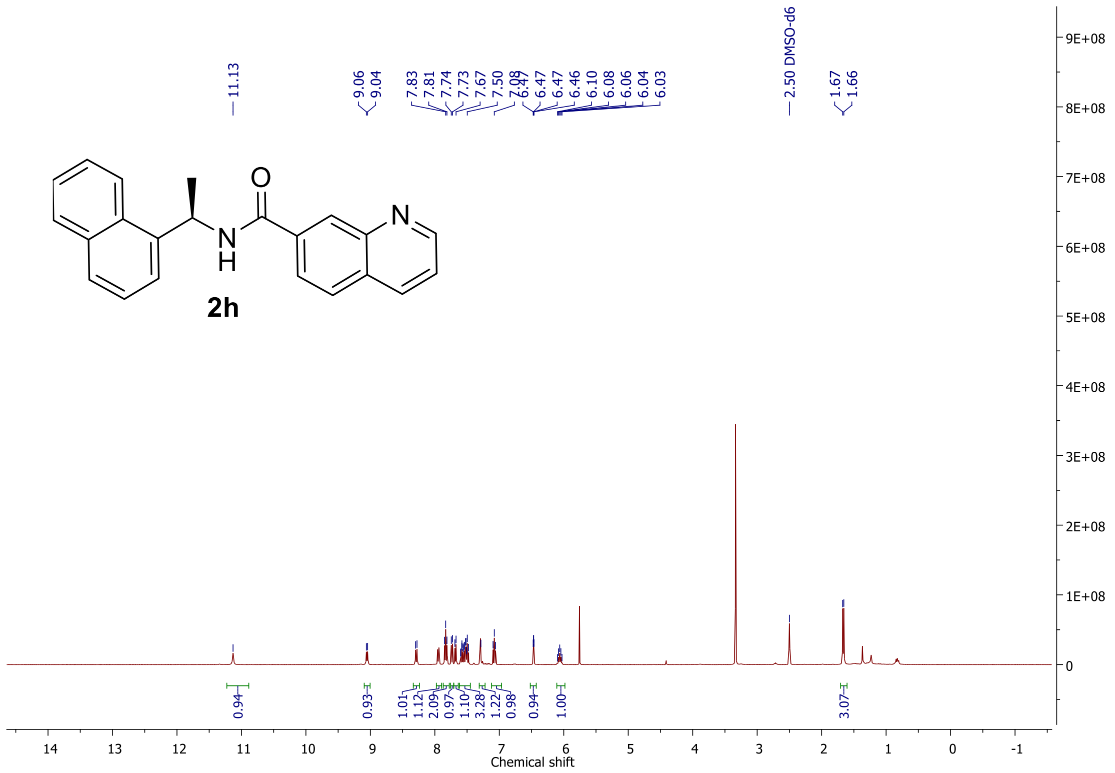

Figure S16. ^{1}H NMR spectrum (DMSO-*d6*, 400 MHz) of (R)-N-(naphthalen-1-yl)ethyl)quinoline-7-carboxamide **2h**

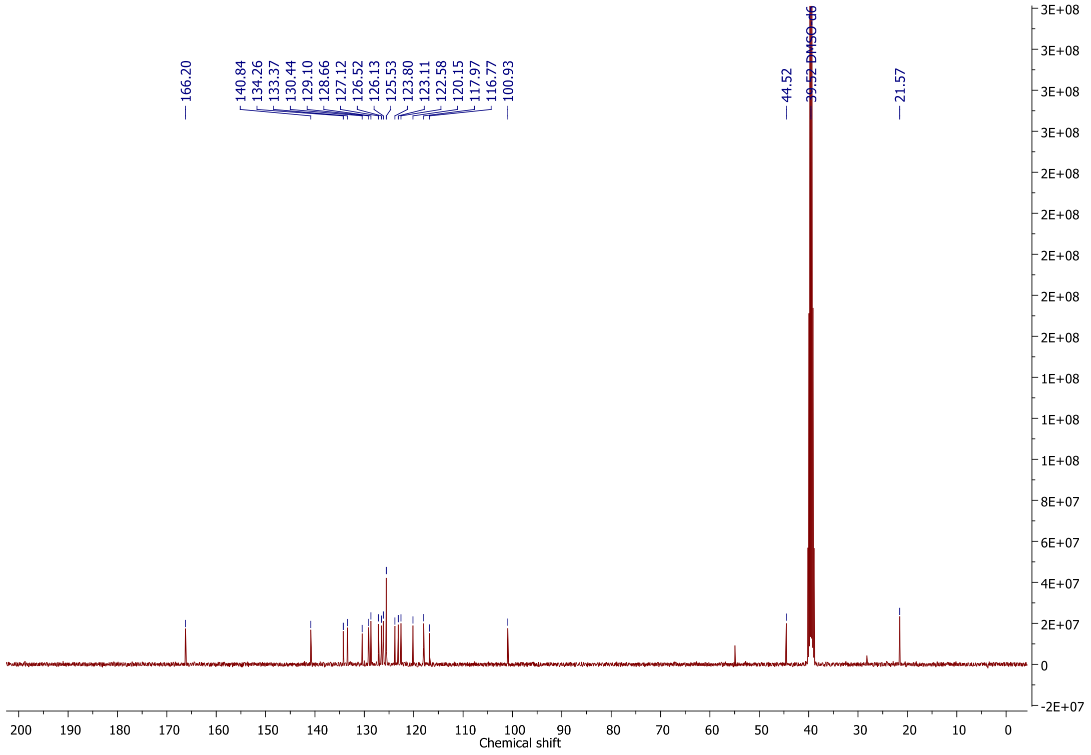

Figure S17. ^{13}C NMR spectrum (DMSO-*d6*, 101 MHz) of (R)-N-(naphthalen-1-yl)ethyl)quinoline-7-carboxamide **2h**

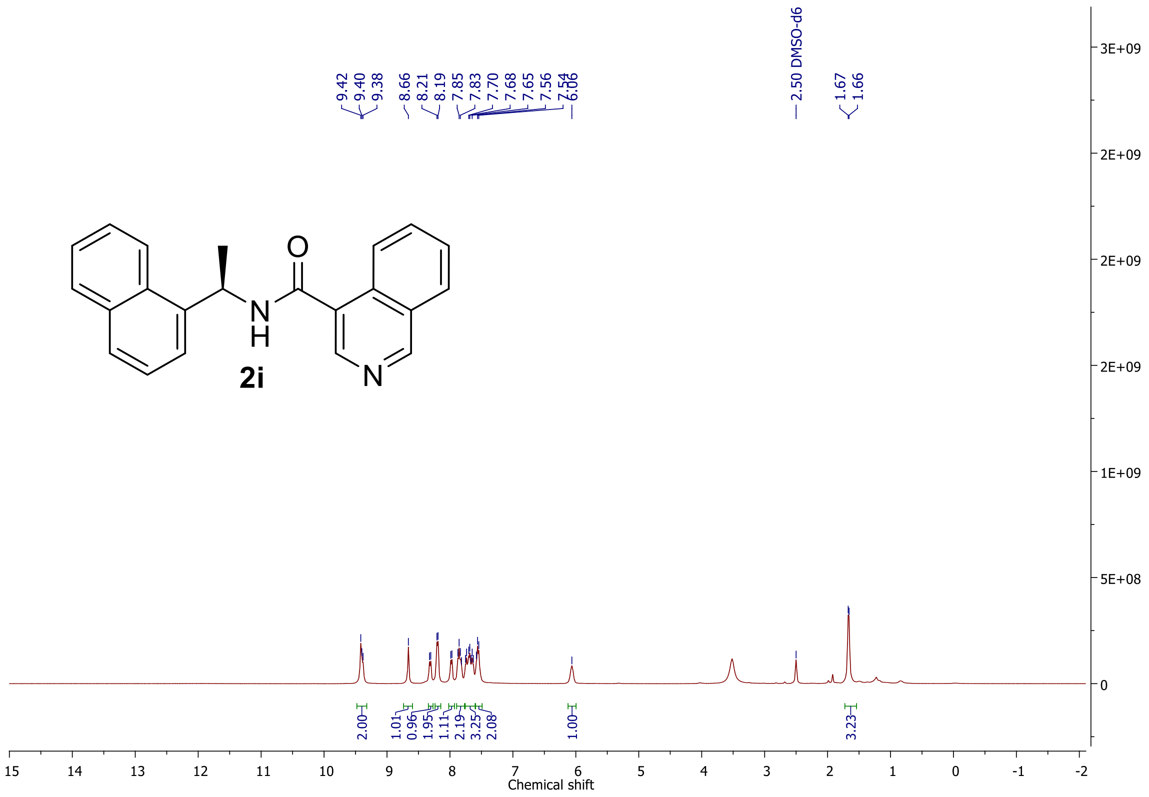

Figure S18. ^{1}H NMR spectrum (DMSO-*d6*, 400 MHz) of (R)-N-(naphthalen-1-yl)ethyl)isoquinoline-4-carboxamide **2i**

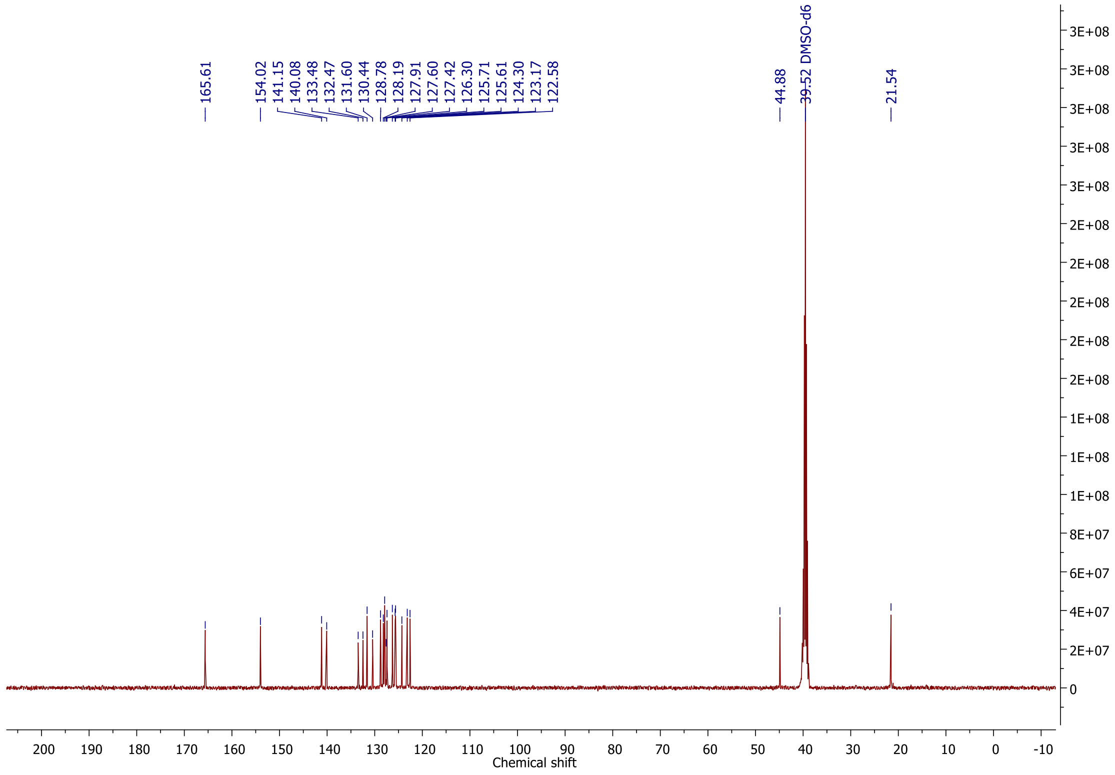

Figure S19. ^{13}C NMR spectrum (DMSO-*d6*, 101 MHz) of (R)-N-(naphthalen-1-yl)ethyl)isoquinoline-4-carboxamide **2i**

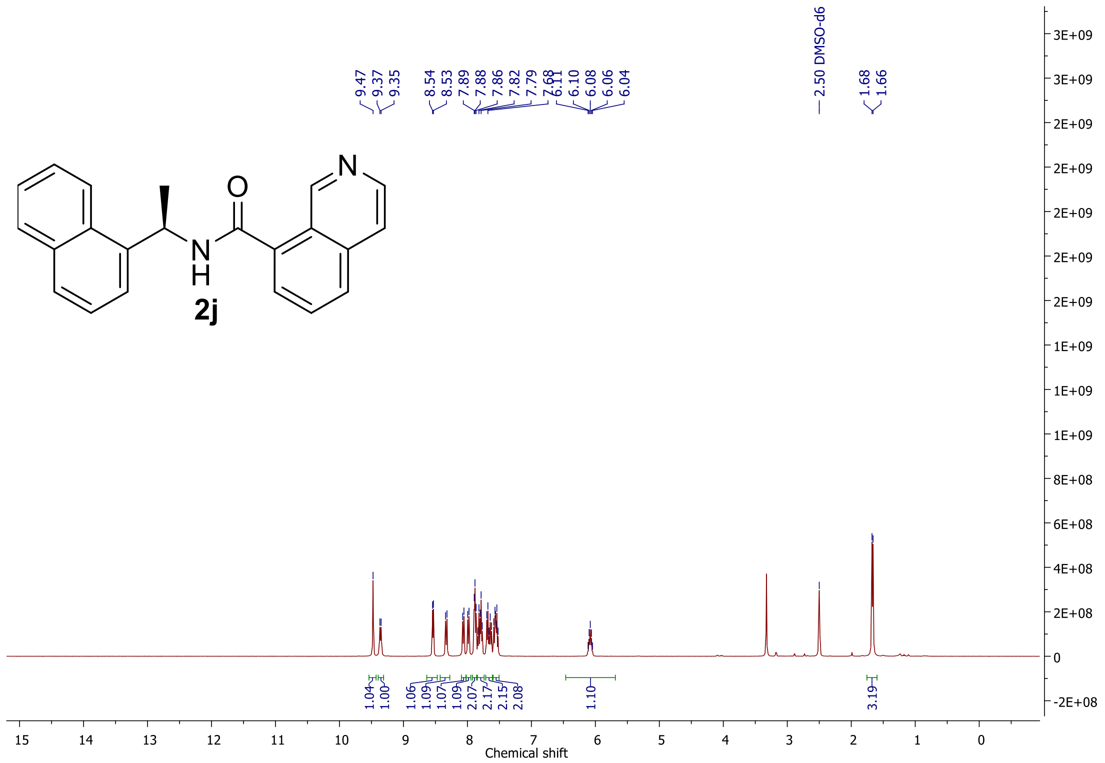

Figure S20. ^{1}H NMR spectrum (DMSO-*d6*, 400 MHz) of (R)-N-(1-(naphthalen-1-yl)ethyl)isoquinoline-8-carboxamide **2j**

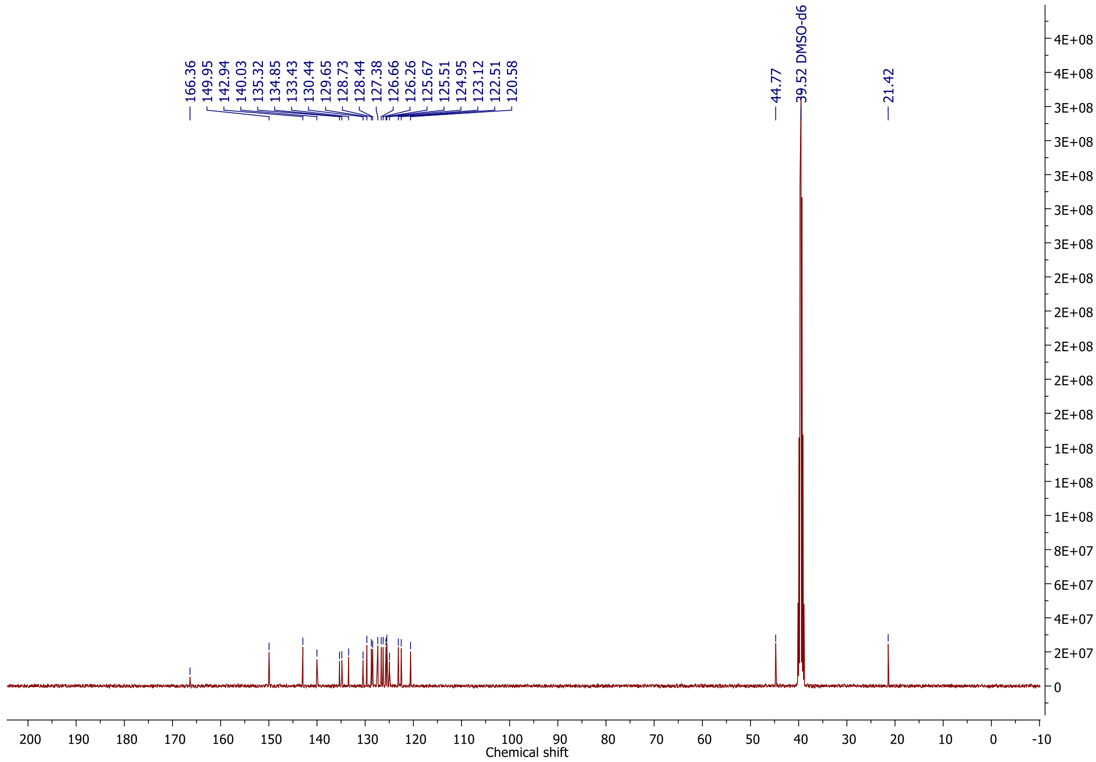

Figure S21. ^{13}C NMR spectrum (DMSO-*d6*, 101 MHz) of (R)-N-(1-(naphthalen-1-yl)ethyl)isoquinoline-8-carboxamide **2j**

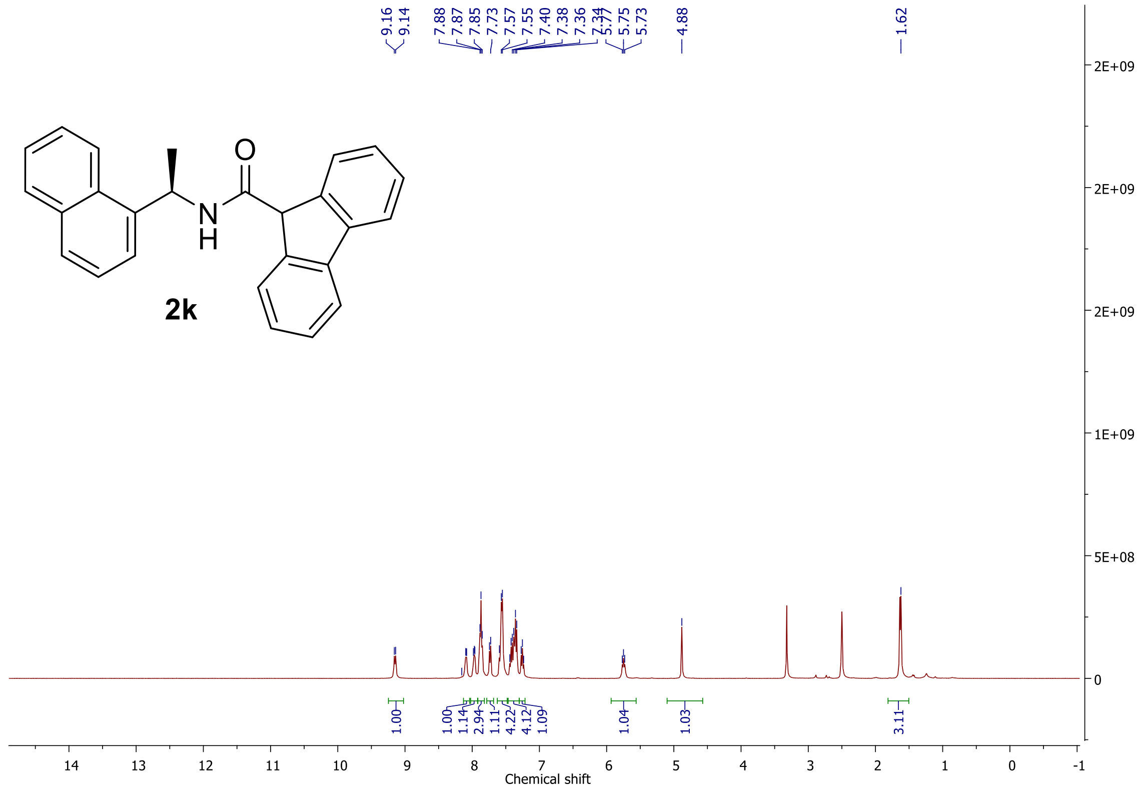

Figure S22. ^{1}H NMR spectrum (DMSO-*d6*, 400 MHz) of (R)-N-(1-(naphthalen-1-yl)ethyl)-9H-fluorene-9 carboxamide **2k**

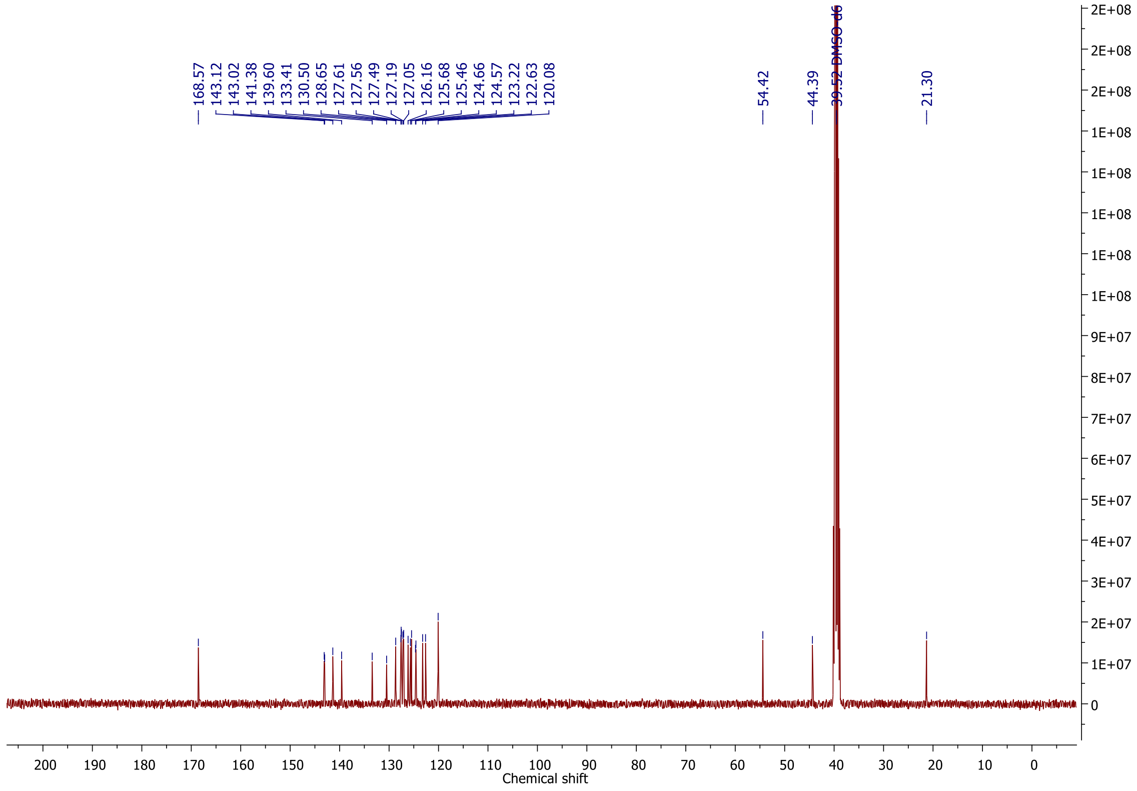

Figure S23. ^{13}C NMR spectrum (DMSO-*d6*, 101 MHz) of (R)-N-(1-(naphthalen-1-yl)ethyl)-9H-fluorene-9 carboxamide **2k**

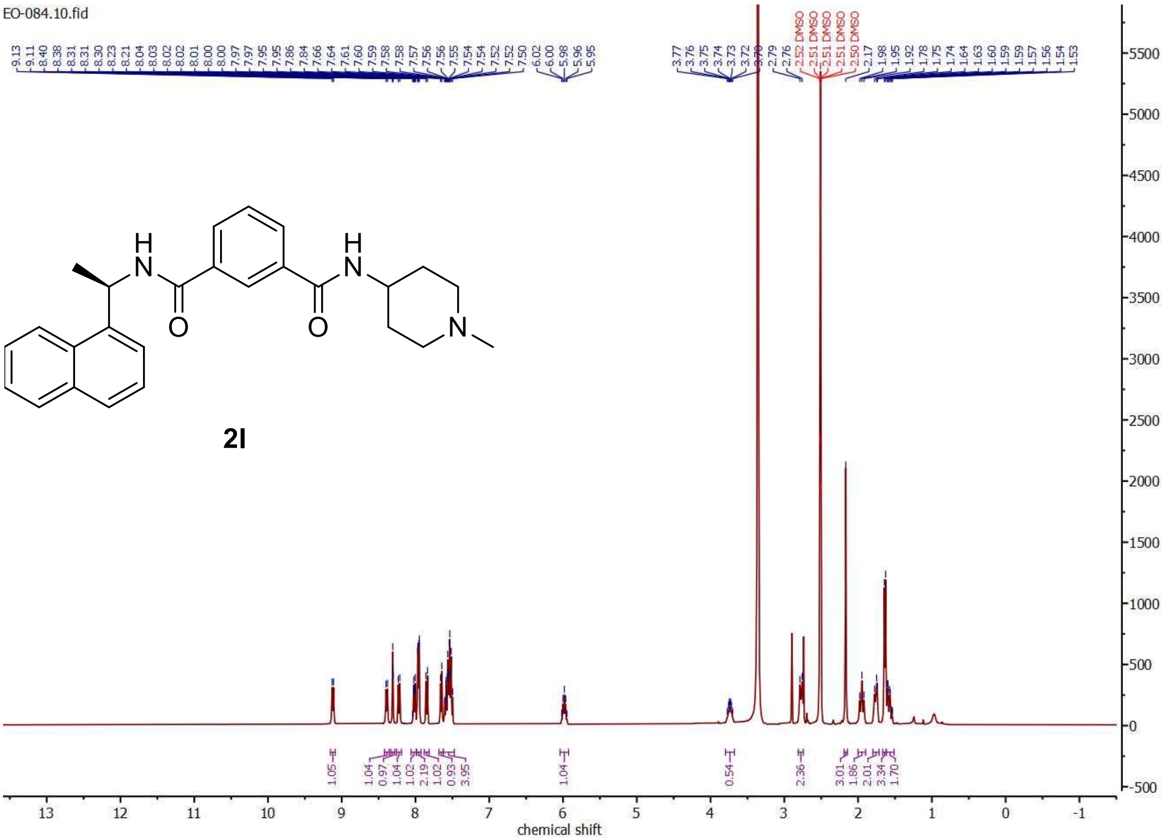

Figure S24. ^{1}H NMR spectrum (DMSO-*d6*, 400 MHz) of (R)-N^{1}-(1-methylpiperidin-4-yl)-N^{3}-(1-(naphthalen-1-yl)ethyl)isophthalamide **2l**

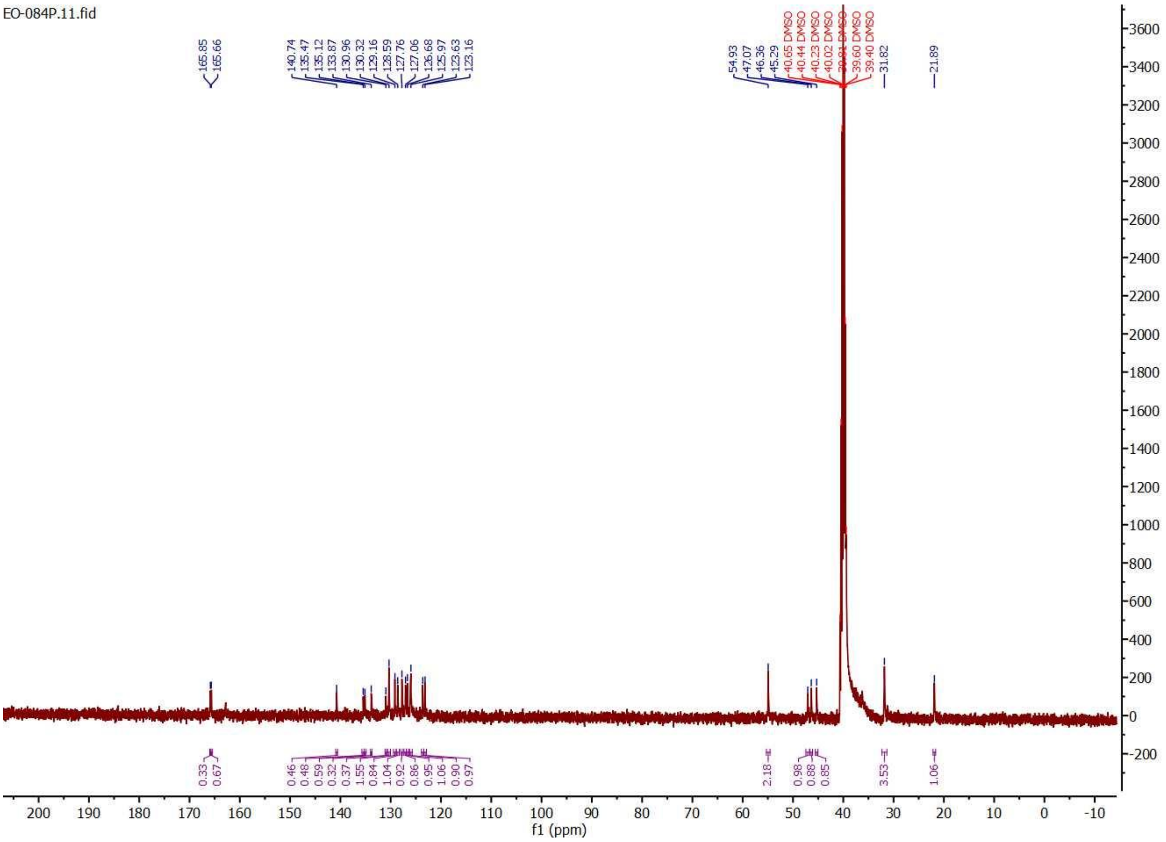

Figure S25. ^{13}C NMR spectrum (DMSO-*d6*, 101 MHz) of (R)-N^{1}-(1-methylpiperidin-4-yl)-N^{3}-(1-(naphthalen-1-yl)ethyl)isophthalamide **2l**

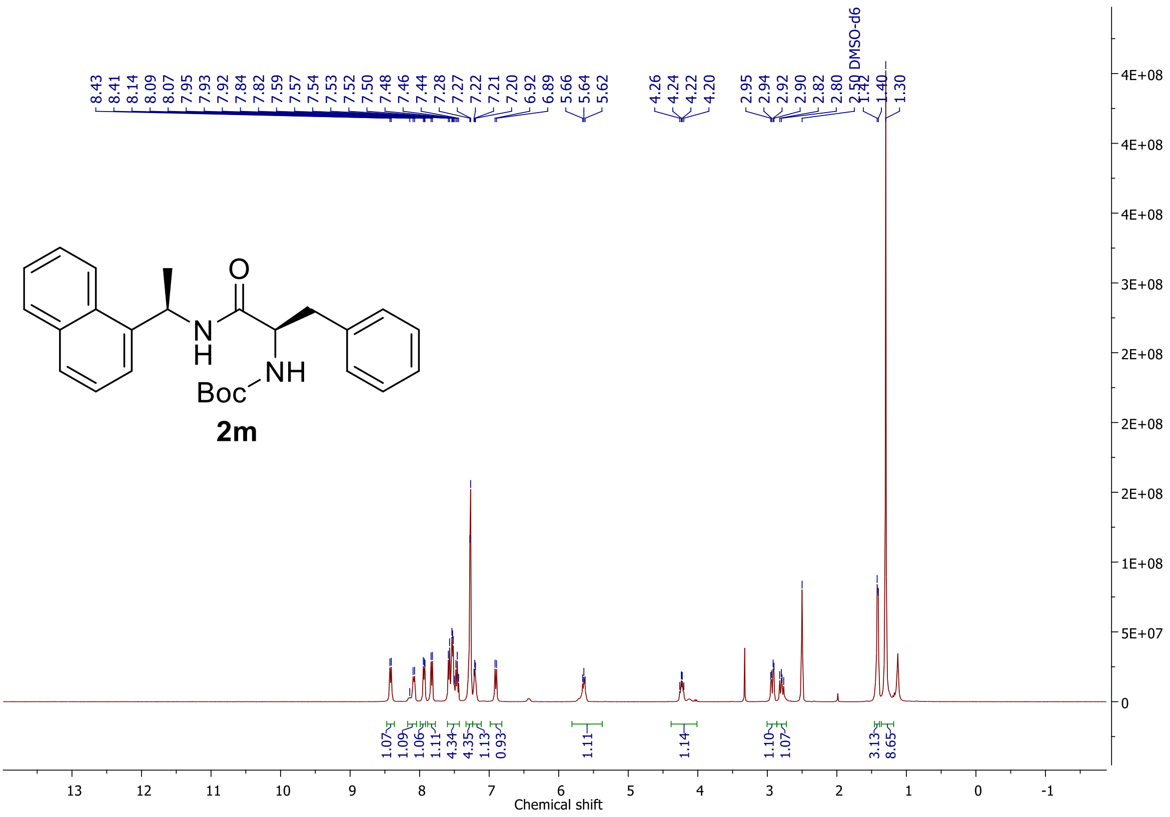

Figure S26. ^{1}H NMR spectrum (DMSO-*d6*, 400 MHz) of Tert-butyl((R)-1-(((R)-1-(naphthalen-1-yl)ethyl)amino)-1-oxo-3-phenylpropan-2-yl)carbamate **2m**

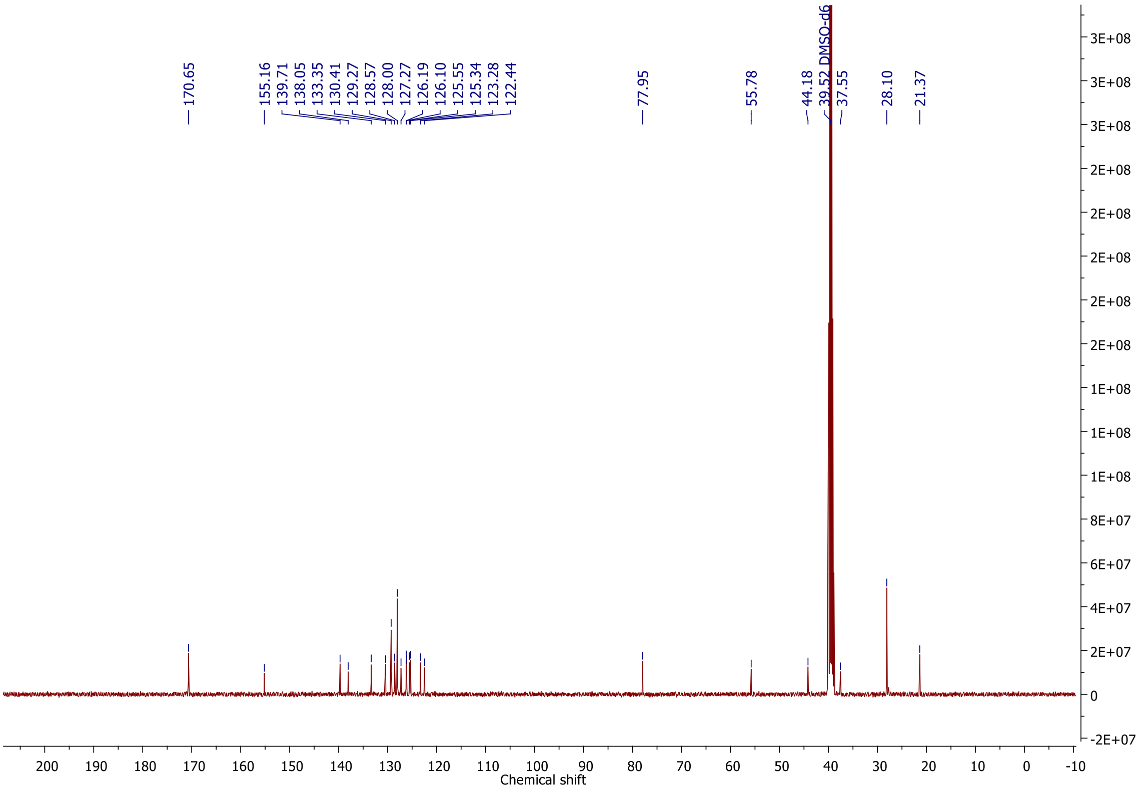

Figure S27. ^{13}C NMR spectrum (DMSO-*d6*, 101 MHz) of Tert-butyl((R)-1-(((R)-1-(naphthalen-1-yl)ethyl)amino)-1-oxo-3-phenylpropan-2-yl)carbamate **2m**

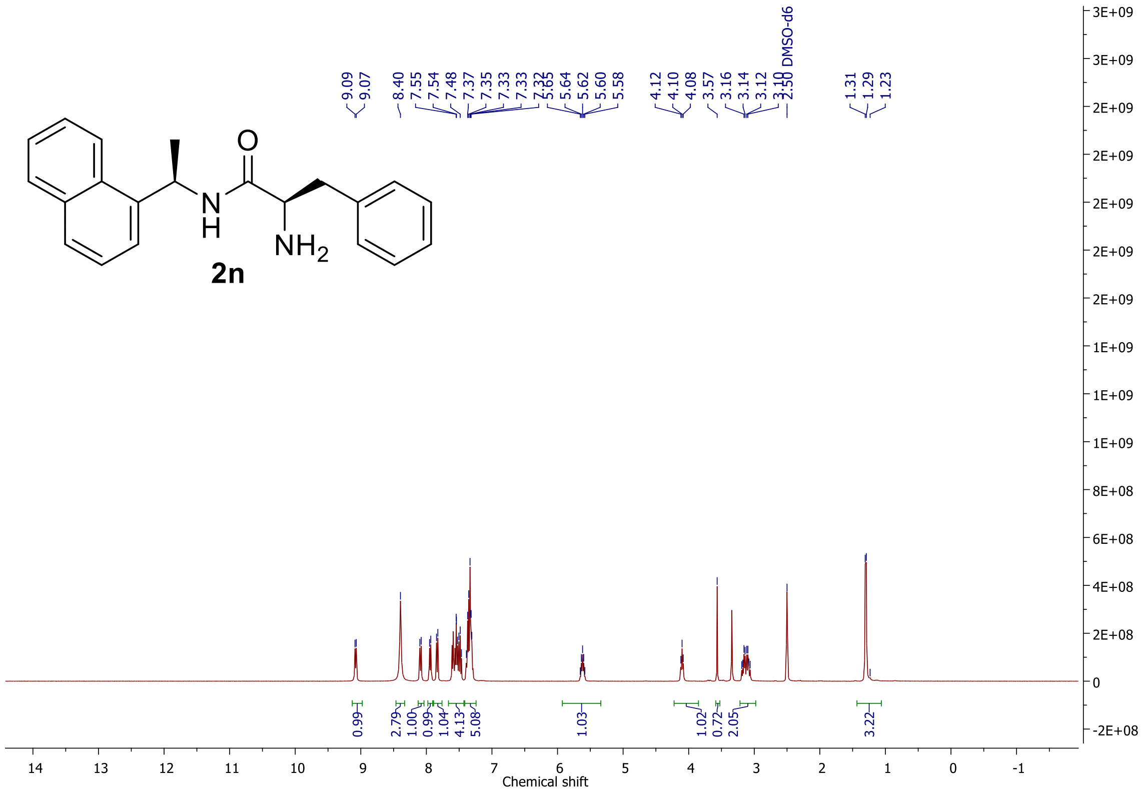

Figure S28. ^{1}H NMR spectrum (DMSO-*d6*, 400 MHz) of (R)-2-amino-N-((R)-naphthalen-1-yl)ethyl)-3-phenylpropanamide **2n**

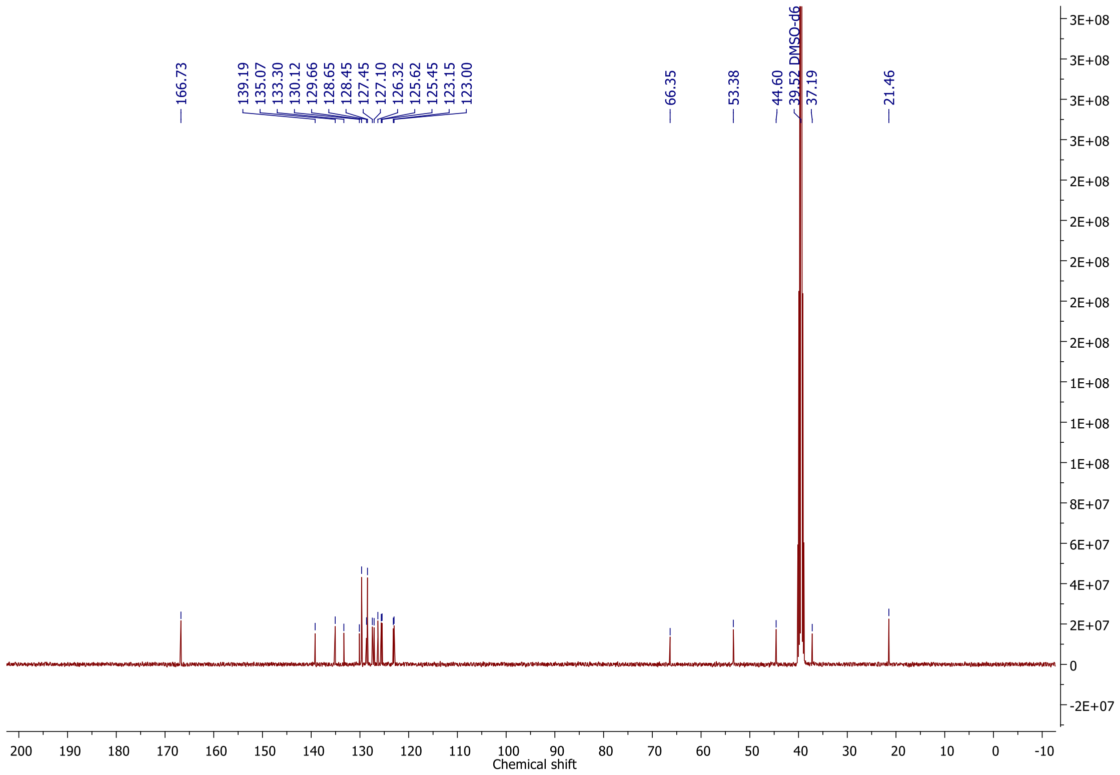

Figure S29. ^{13}C NMR spectrum (DMSO-*d6*, 101 MHz) of (R)-2-amino-N-((R)-naphthalen-1-yl)ethyl)-3-phenylpropanamide **2n**

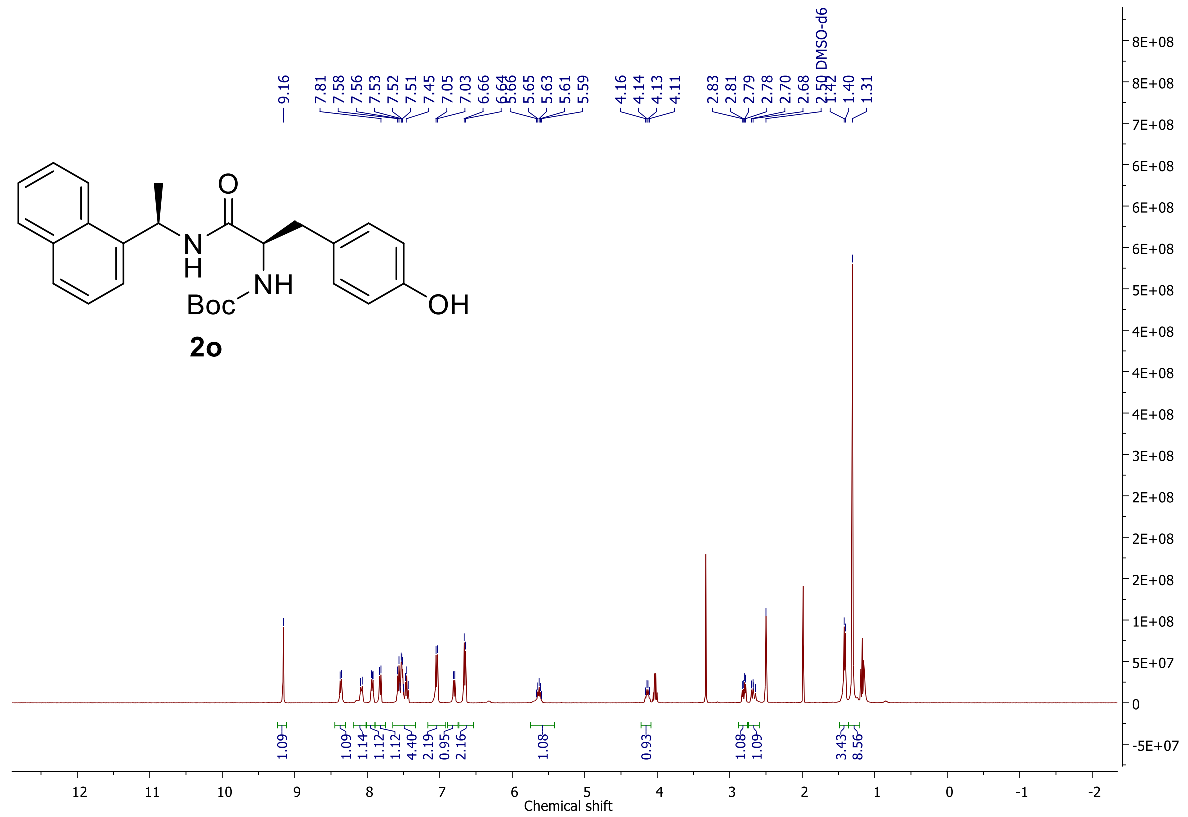

Figure S30. ^{1}H NMR spectrum (DMSO-*d6*, 400 MHz) of *tert*-butyl((R)-3-(4-hydroxyphenyl)-1-(((R)-1-(naphthalen-1-yl)ethyl)amino)-1-oxopropan-2-yl)carbamate **2o**

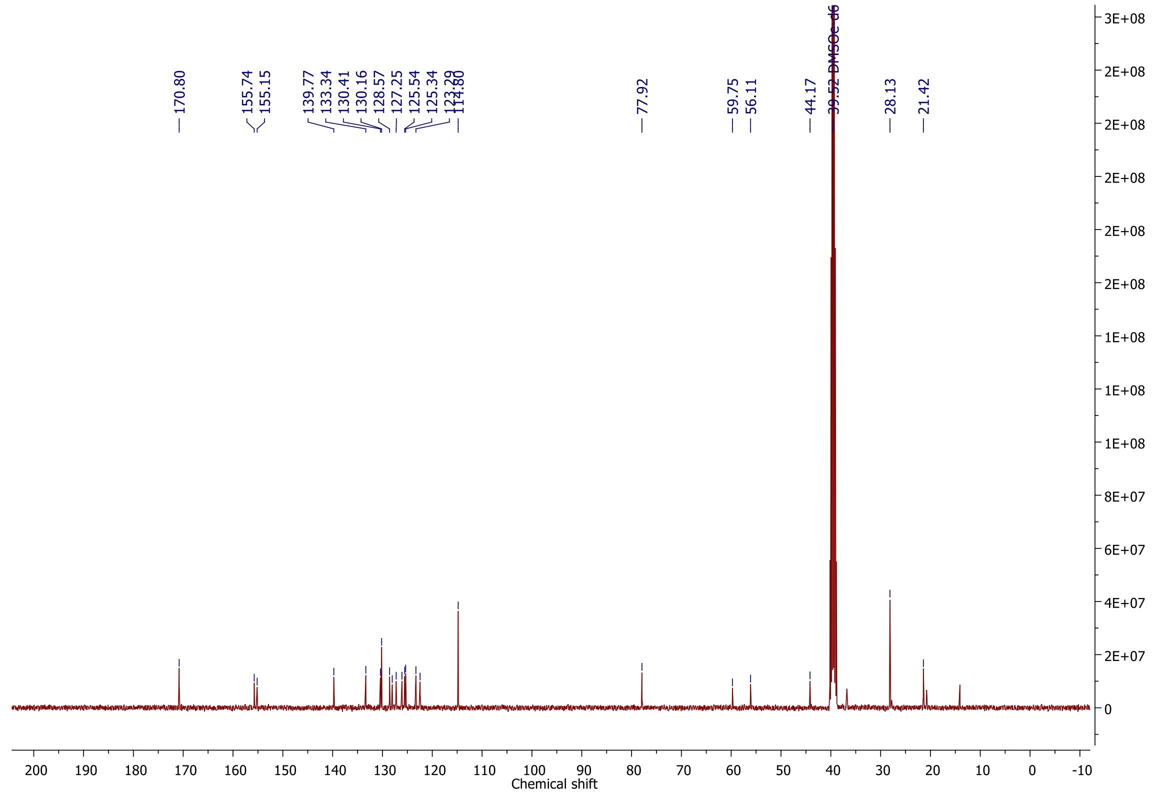

Figure S31. ^{13}C NMR spectrum (DMSO-*d6*, 101 MHz) of *tert*-butyl((R)-3-(4-hydroxyphenyl)-1-(((R)-1-(naphthalen-1-yl)ethyl)amino)-1-oxopropan-2-yl)carbamate **2o**

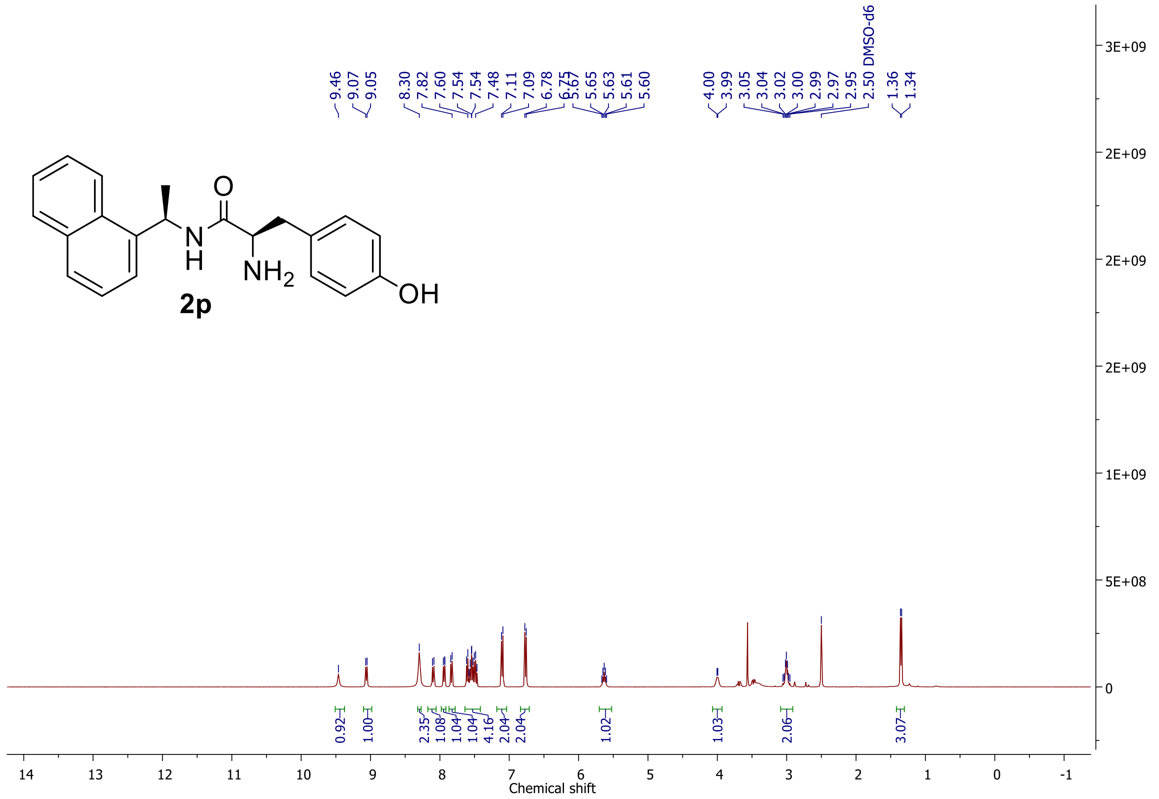

Figure S32. ^{1}H NMR spectrum (DMSO-*d6*, 400 MHz) of (R)-2-amino-3-(4-hydroxyphenyl)-N-((R)-1-(naphthalen-1-yl)ethyl)propanamide **2p**

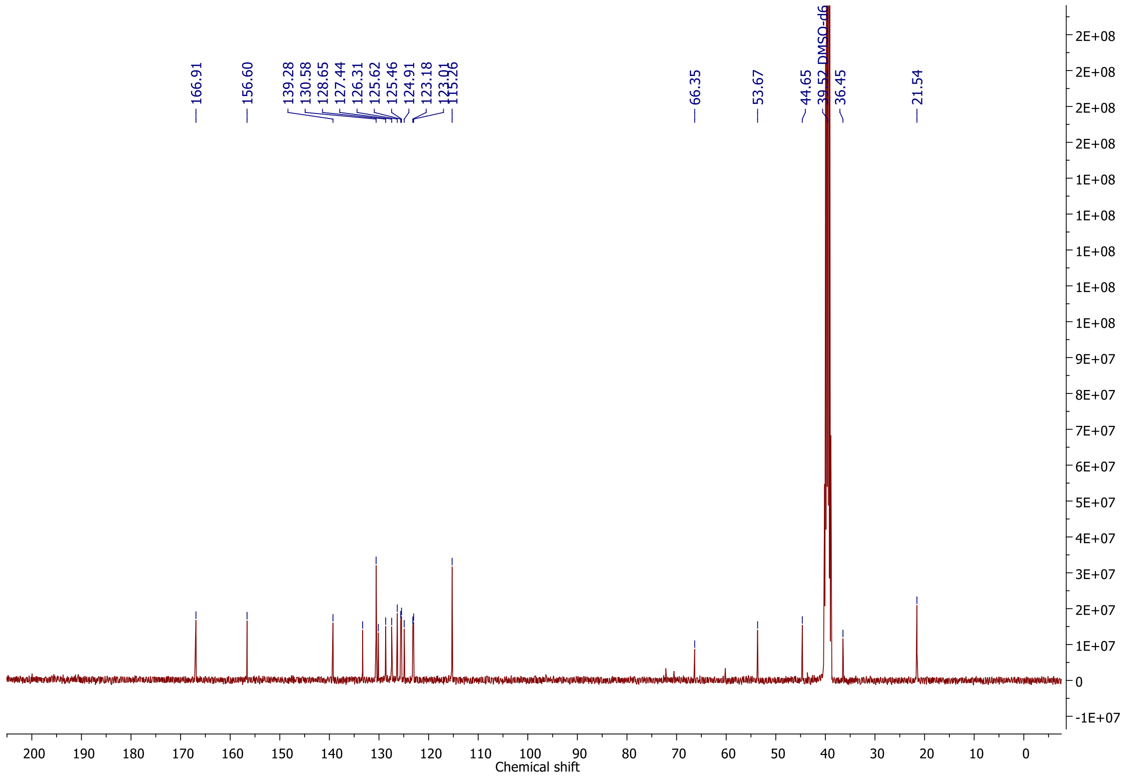

Figure S33. ^{13}C NMR spectrum (DMSO-*d6*, 101 MHz) of (R)-2-amino-3-(4-hydroxyphenyl)-N-((R)-1-(naphthalen-1-yl)ethyl)propanamide **2p**

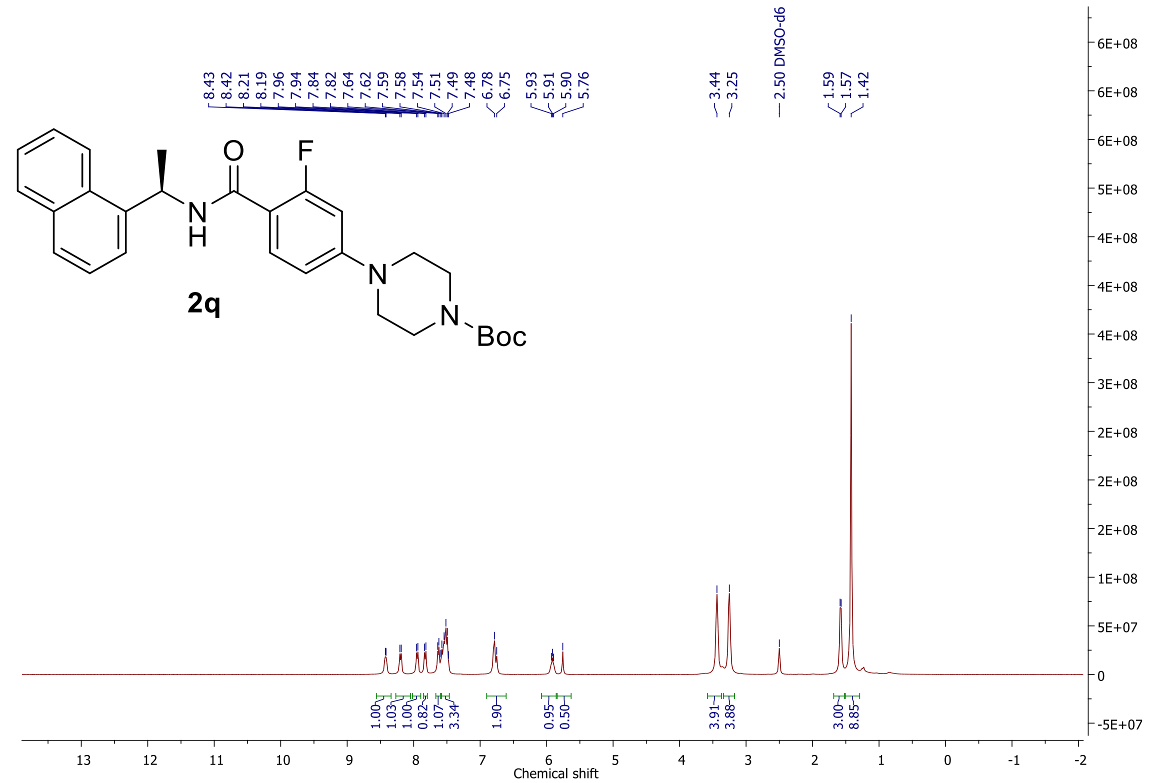

Figure S34. ^{1}H NMR spectrum (DMSO-*d6*, 400 MHz) of (R)-tert-butyl-4-(3-fluoro-4-((1-(naphthalen-1-yl)ethyl)carbamoyl)phenyl)-piperazine-1-carboxylate **2q**

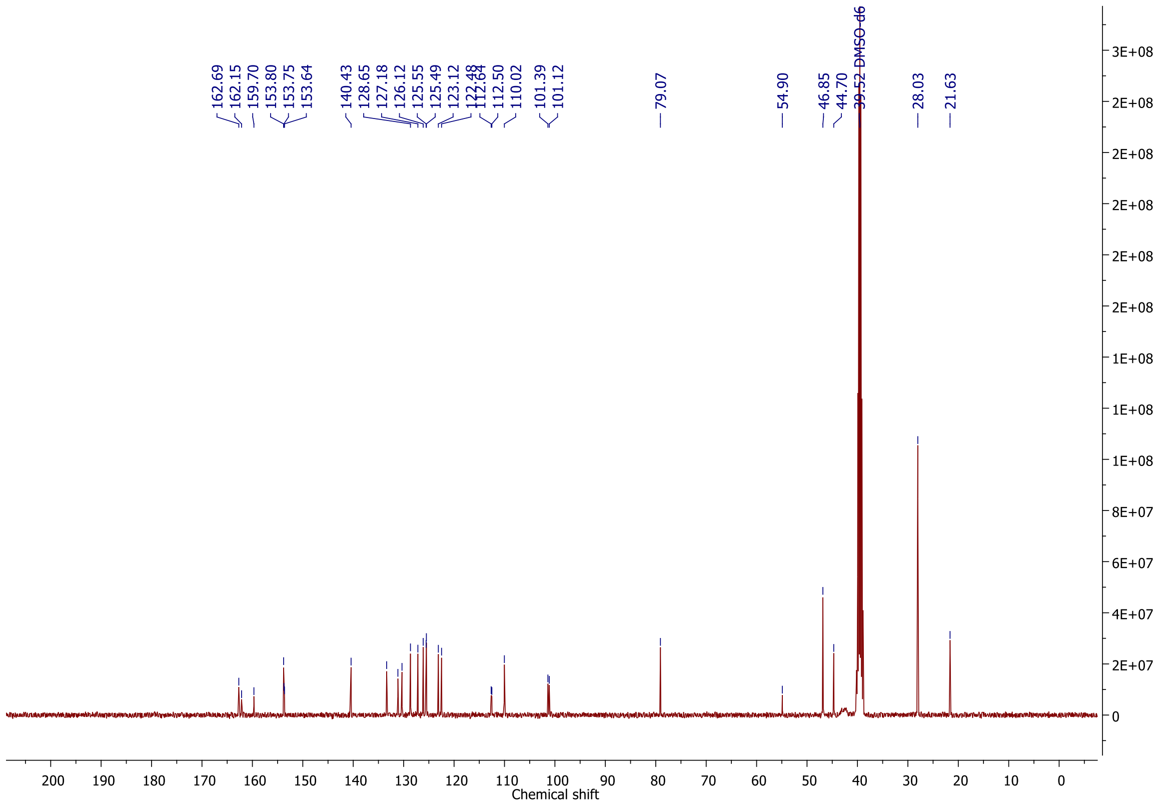

Figure S35. ^{13}C NMR spectrum (DMSO-*d6*, 101 MHz) of (R)-tert-butyl-4-(3-fluoro-4-((1-(naphthalen-1-yl)ethyl)carbamoyl)phenyl)-piperazine-1-carboxylate **2q**

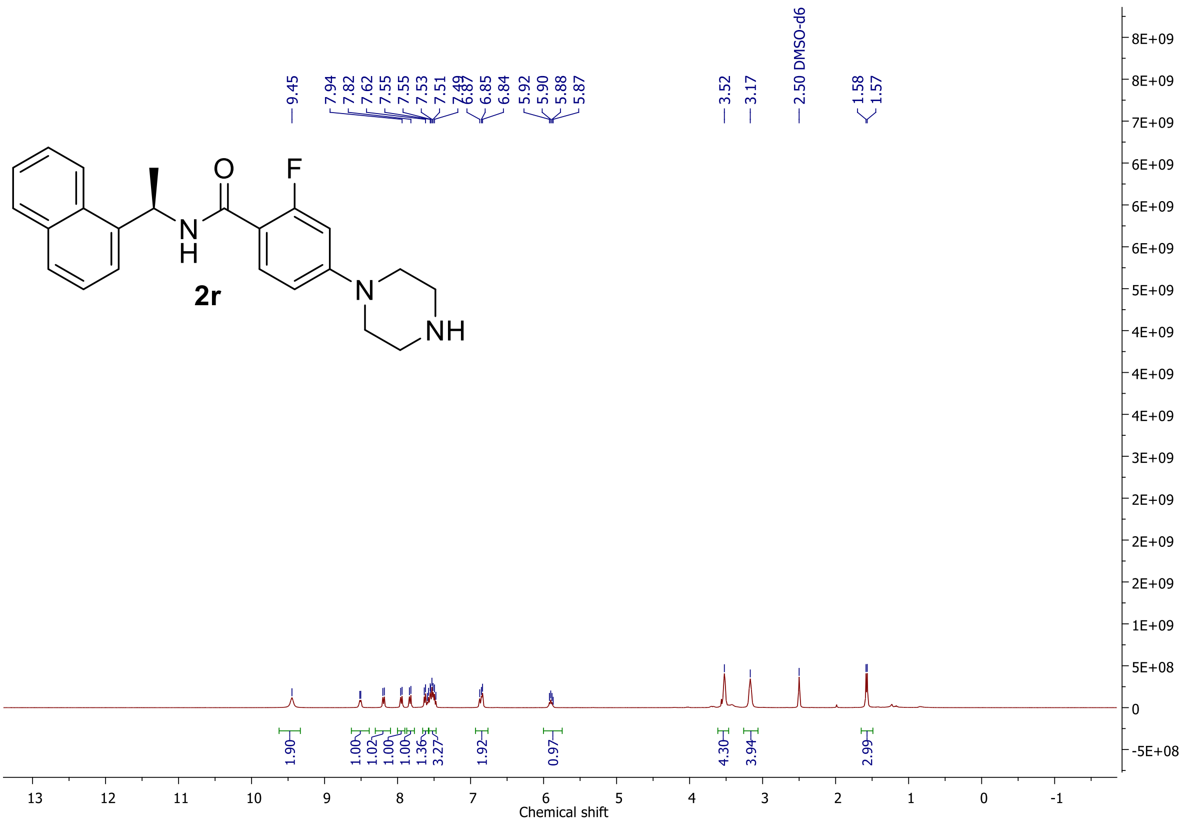

Figure S36. ^{1}H NMR spectrum (DMSO-*d6*, 400 MHz) of (R)-2-fluoro-N-(1-(naphthalen-1-yl)ethyl)-4-(piperazin-1-yl)benzamide **2r**

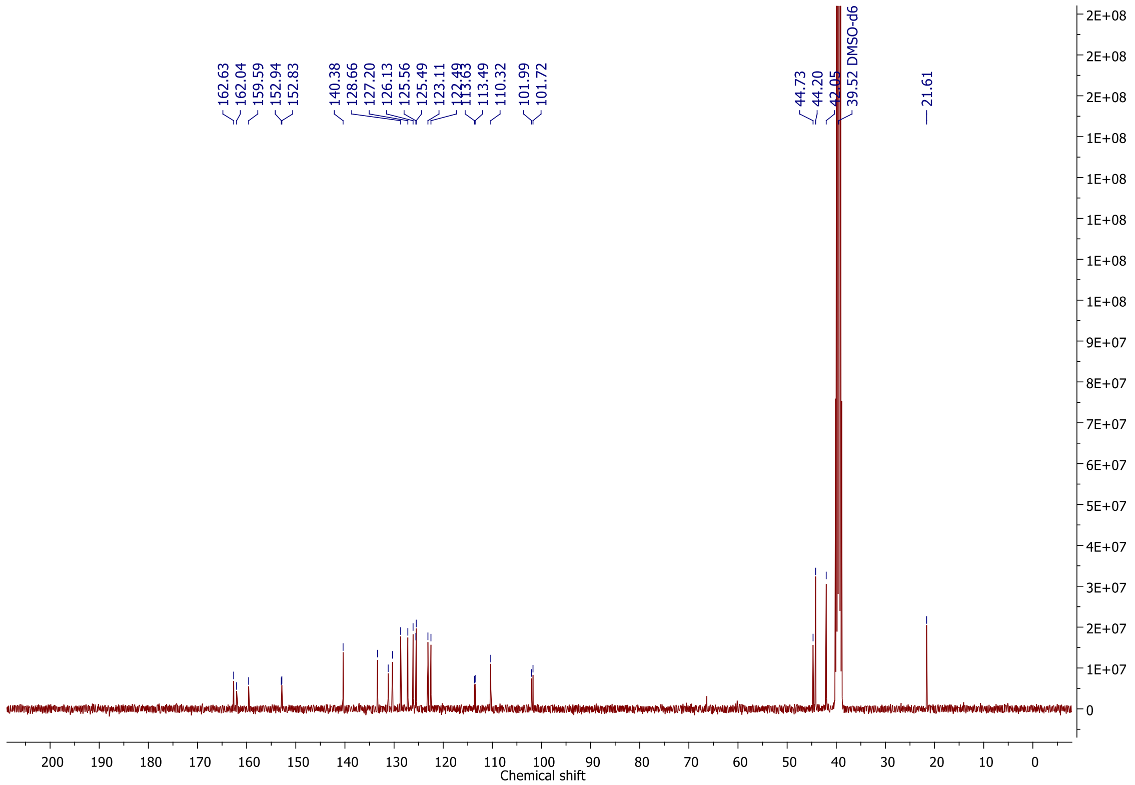

Figure S37. ^{13}C NMR spectrum (DMSO-*d6*, 101 MHz) of (R)-2-fluoro-N-(1-(naphthalen-1-yl)ethyl)-4-(piperazin-1-yl)benzamide **2r**

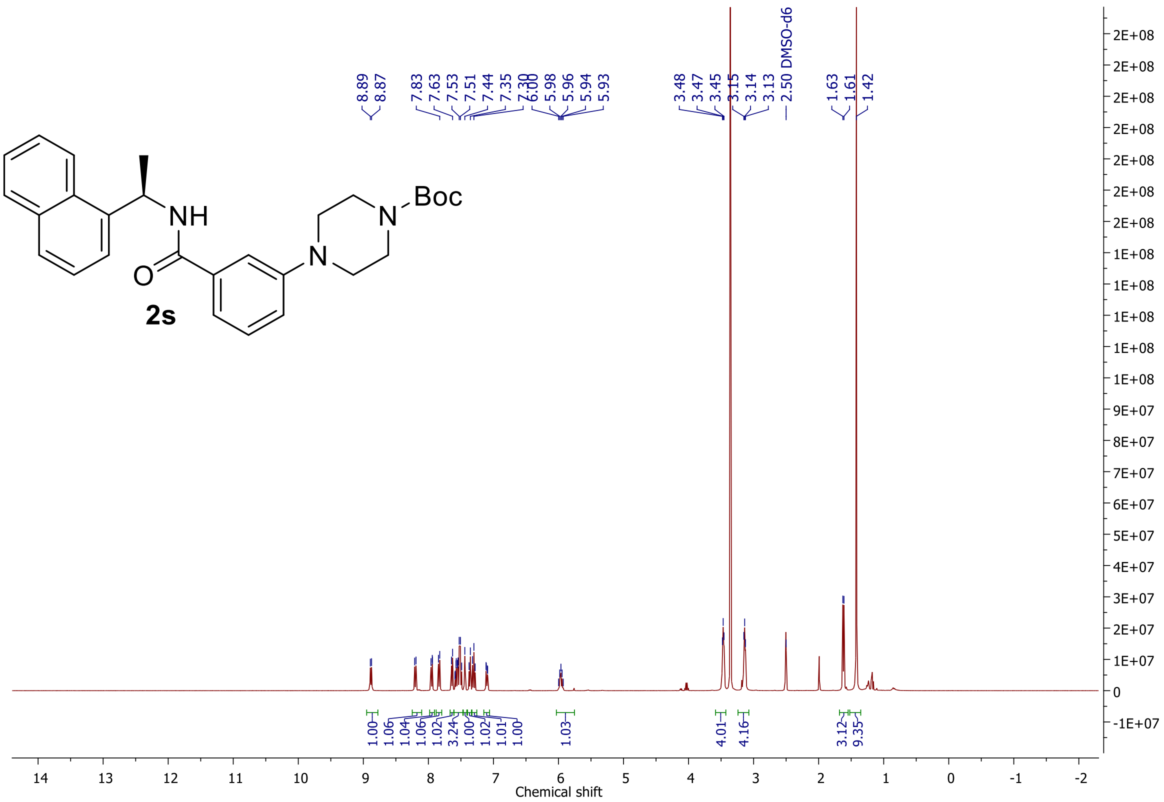

Figure S38. ^{1}H NMR spectrum (DMSO-*d6*, 400 MHz) of (R)-tert-butyl-4-(3-((1-(naphtalen-1-yl)ethyl)carbamoyl)phenyl)piperazine-1-carboxylate **2s**

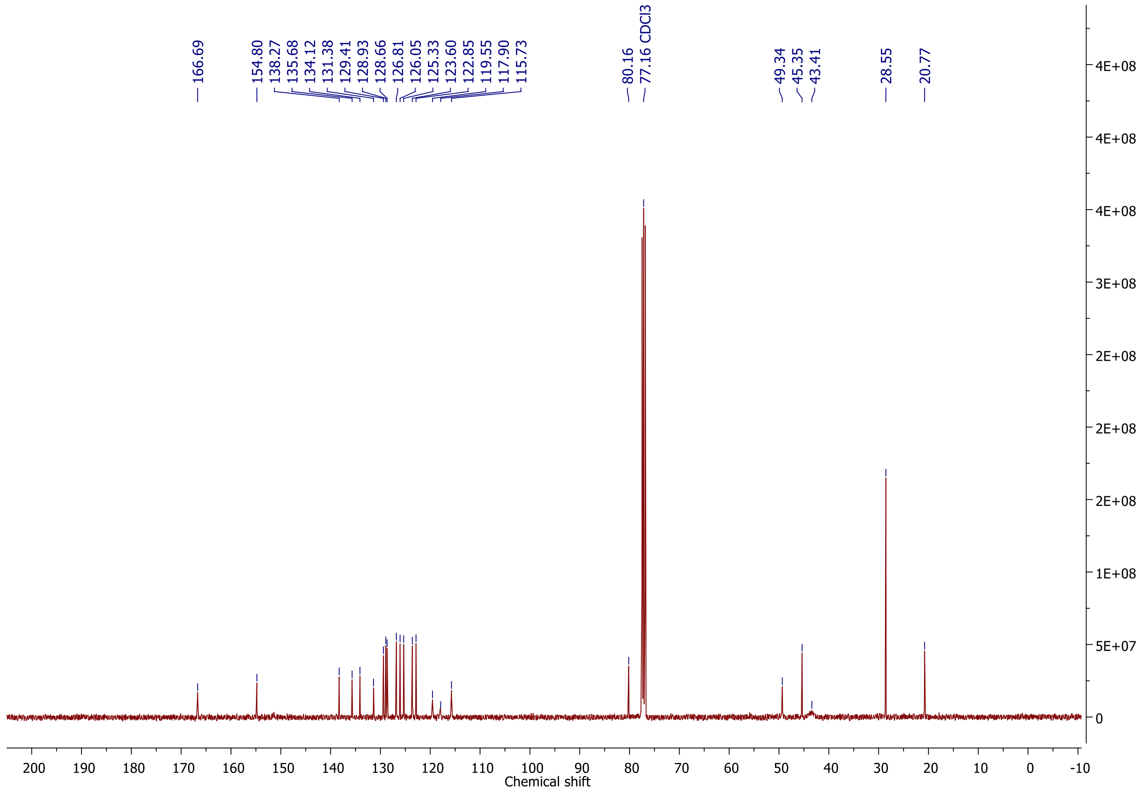

Figure S39. ^{13}C NMR spectrum (DMSO-*d6*, 101 MHz) of (R)-tert-butyl-4-(3-((1-(naphtalen-1-yl)ethyl)carbamoyl)phenyl)piperazine-1-carboxylate **2s**

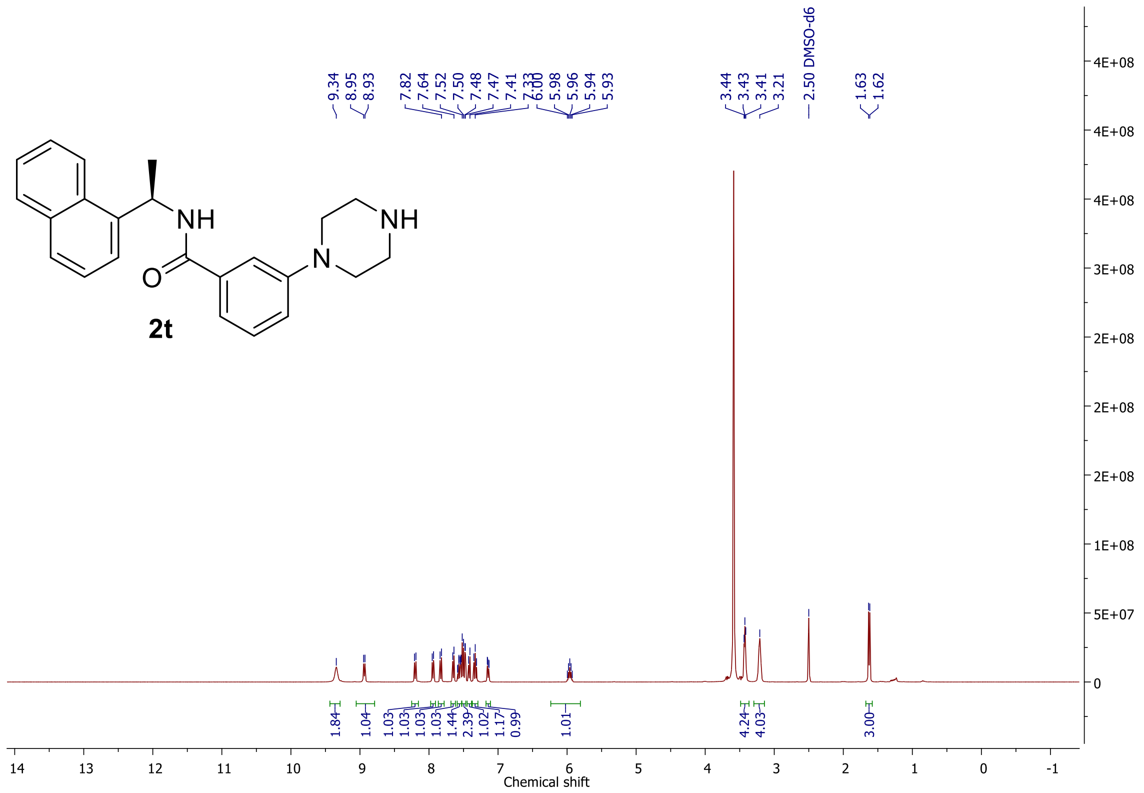

Figure S40. ^{1}H NMR spectrum (DMSO-*d6*, 400 MHz) of (R)-N-(1-(naphthalen-1-yl)ethyl)-3-(piperazin-1-yl)benzamide **2t**

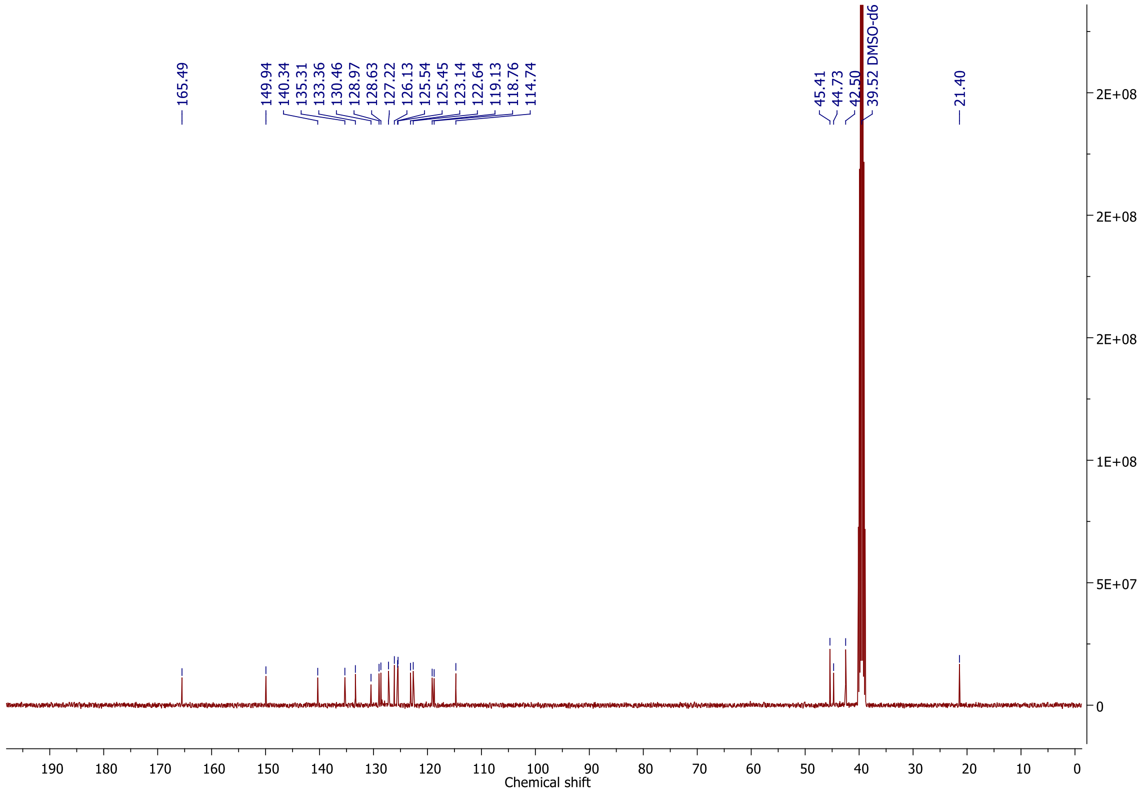

Figure S41. ^{13}C NMR spectrum (DMSO-*d6*, 101 MHz) of (R)-N-(1-(naphthalen-1-yl)ethyl)-3-(piperazin-1-yl)benzamide **2t**

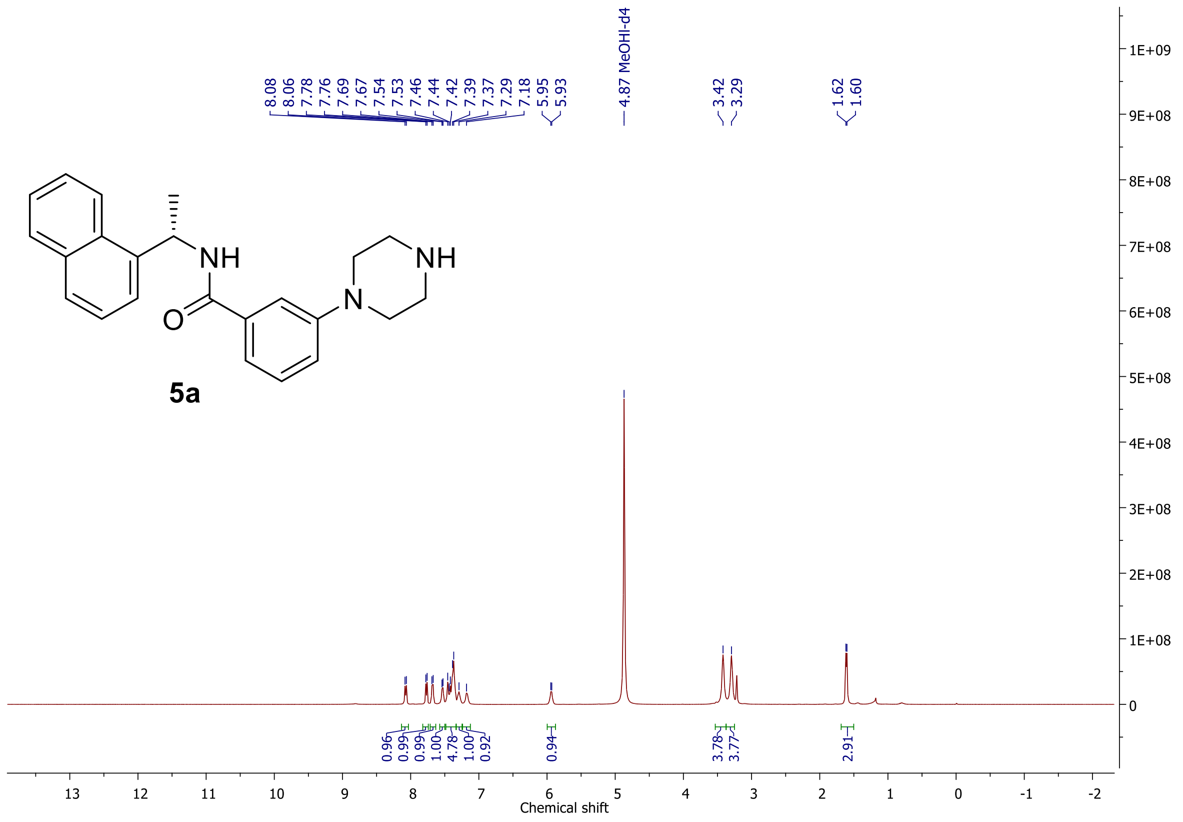

Figure S42. ¹H NMR spectrum (MeOH-*d4*, 400 MHz) of (S)-N-(1-naphthalen-1yl)ethyl)-3-(piperazin-1-yl)benzamide **5a**

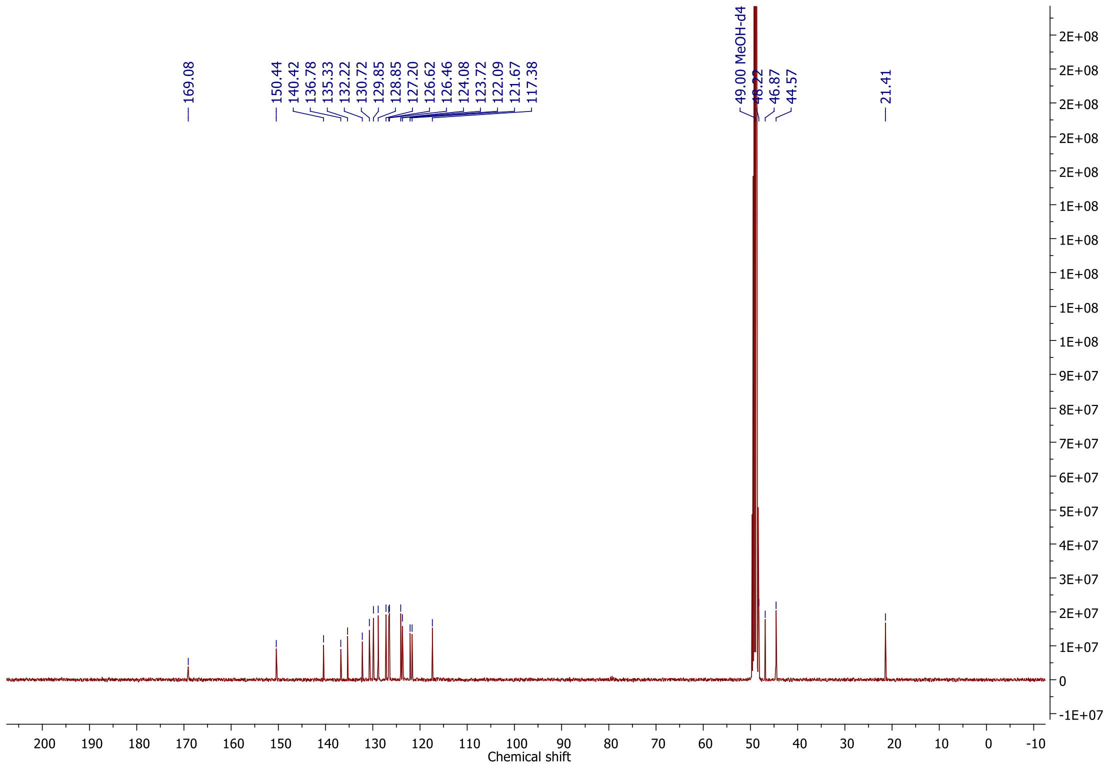

Figure S43. ¹³C NMR spectrum (MeOH-*d4*, 101 MHz) of (S)-N-(1-naphthalen-1yl)ethyl)-3-(piperazin-1-yl)benzamide **5a**

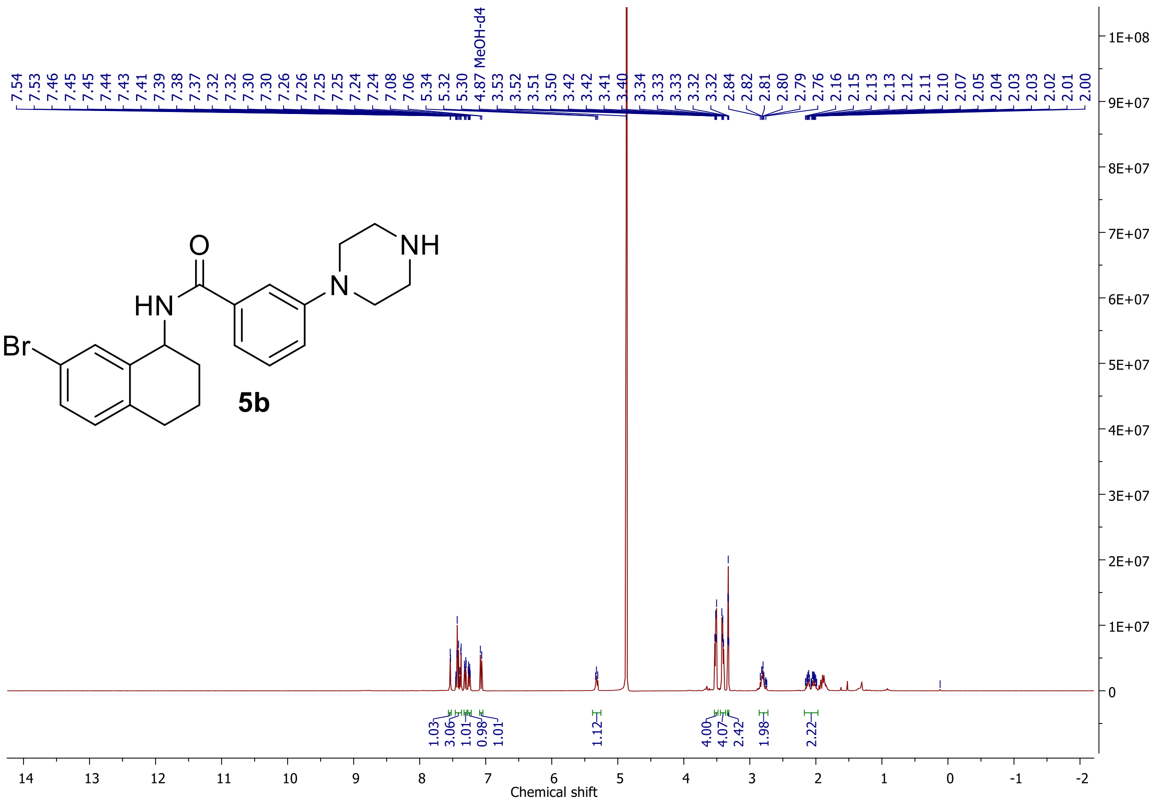

Figure S44. ^{1}H NMR spectrum (MeOH-*d4*, 400 MHz) of *tert*-butyl 4-(3-((7-bromo-1,2,3,4-tetrahydronaphthalen-1-yl)carbamoyl)phenyl)piperazine-1-carboxylate **5b**

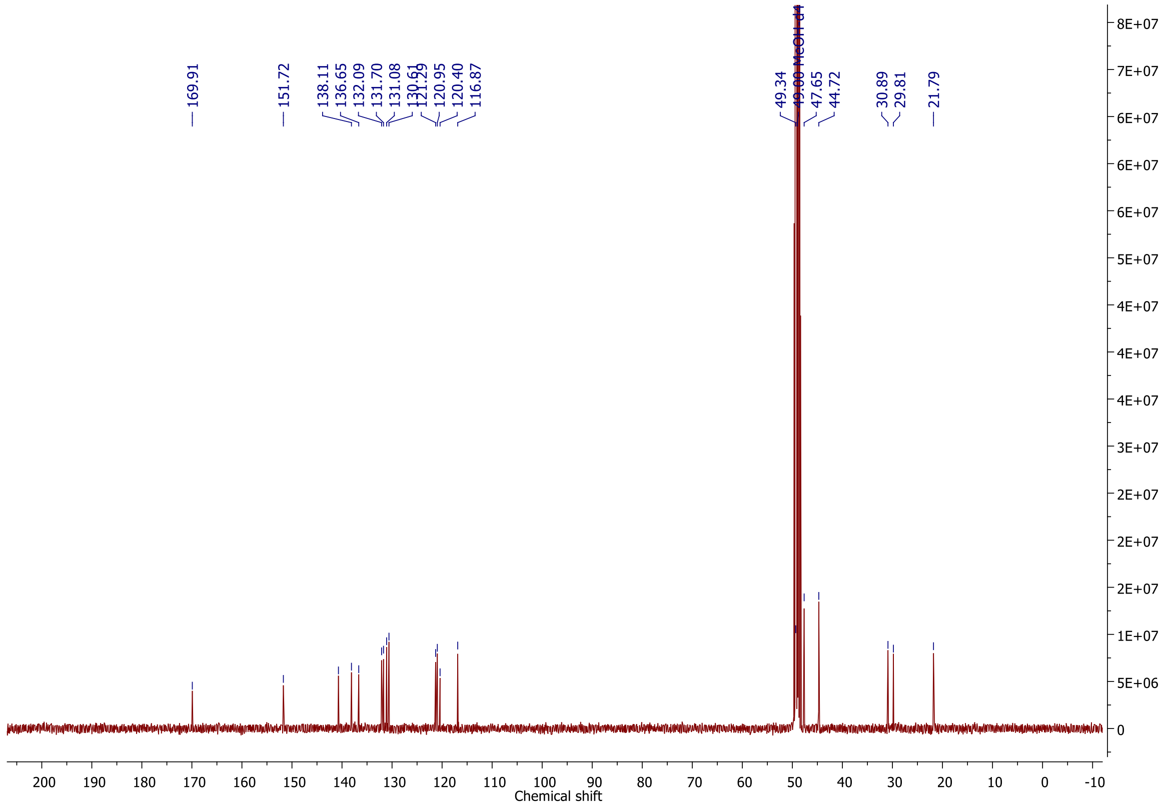

Figure S45. ^{13}C NMR spectrum (MeOH-*d4*, 101 MHz) of *tert*-butyl 4-(3-((7-bromo-1,2,3,4-tetrahydronaphthalen-1-yl)carbamoyl)phenyl)piperazine-1-carboxylate **5b**

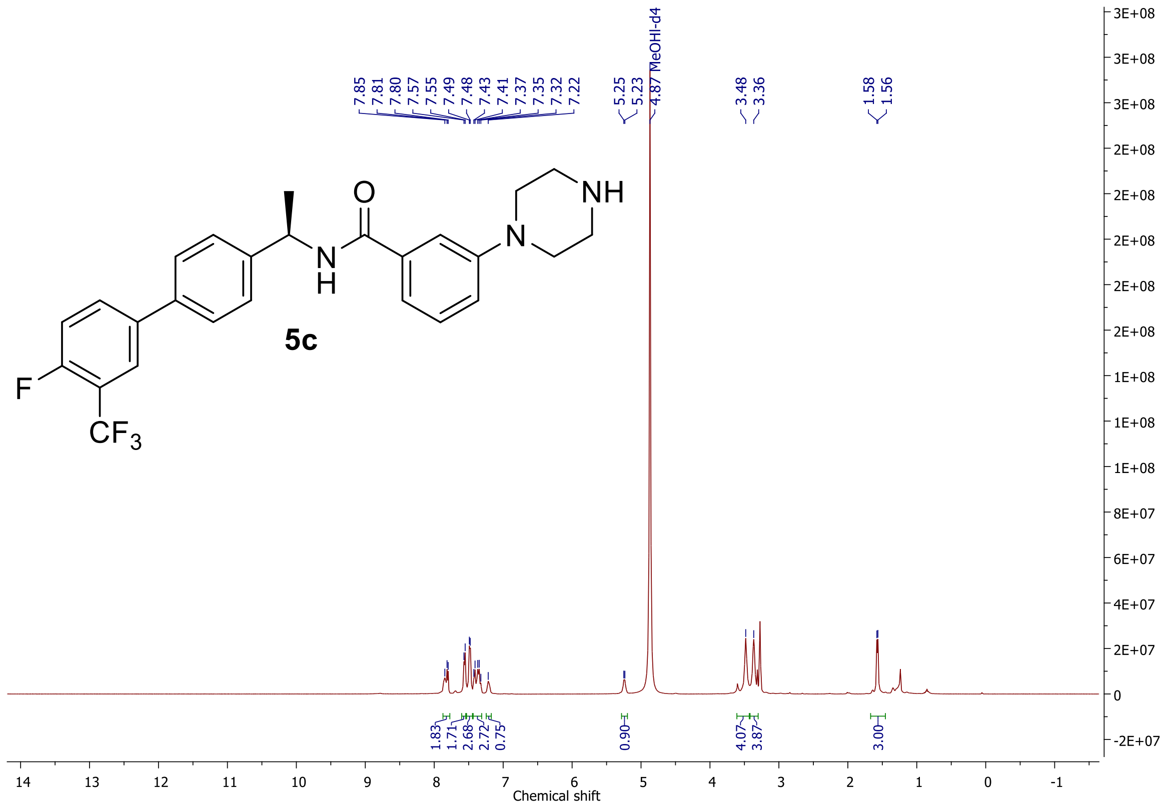

Figure S46. ^{1}H NMR spectrum (MeOH-*d4*, 400 MHz) of *(R)*-N-(1-(4'-fluoro-3'-(trifluoromethyl)-[1,1'-biphenyl]-4-yl)ethyl)-3-(piperazin-1-yl)benzamide **5c**

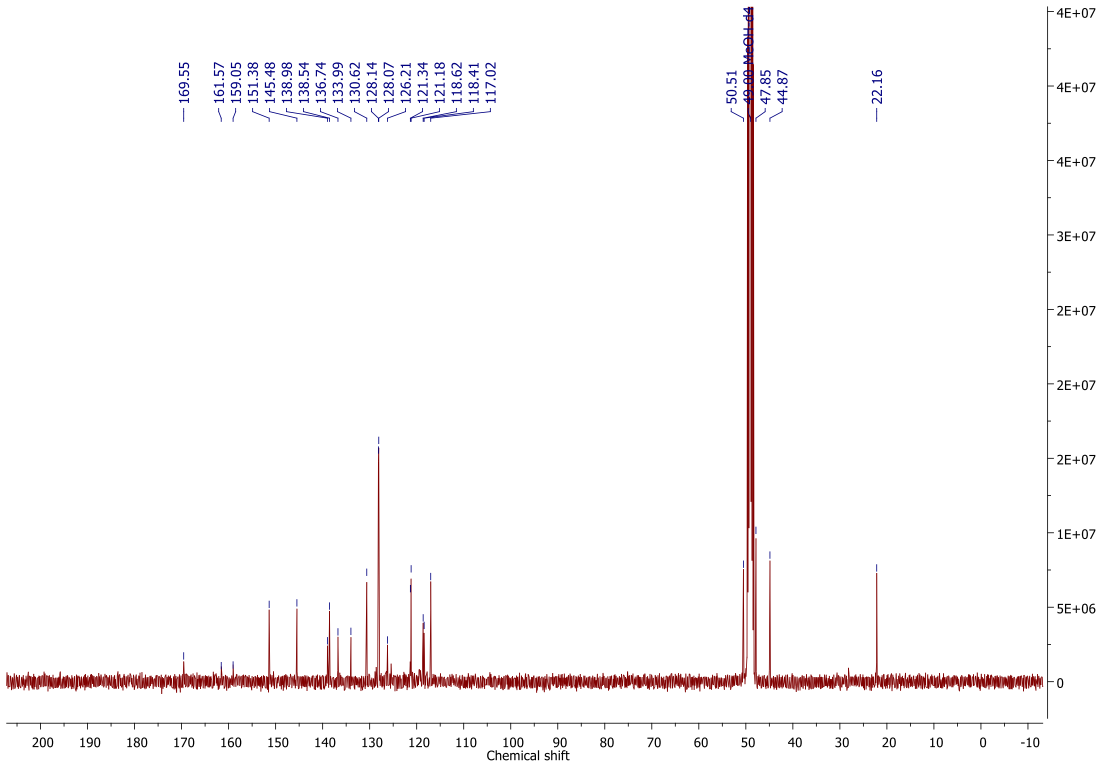

Figure S47. ^{13}C NMR spectrum (MeOH-*d4*, 101 MHz) of *(R)*-N-(1-(4'-fluoro-3'-(trifluoromethyl)-[1,1'-biphenyl]-4-yl)ethyl)-3-(piperazin-1-yl)benzamide **5c**

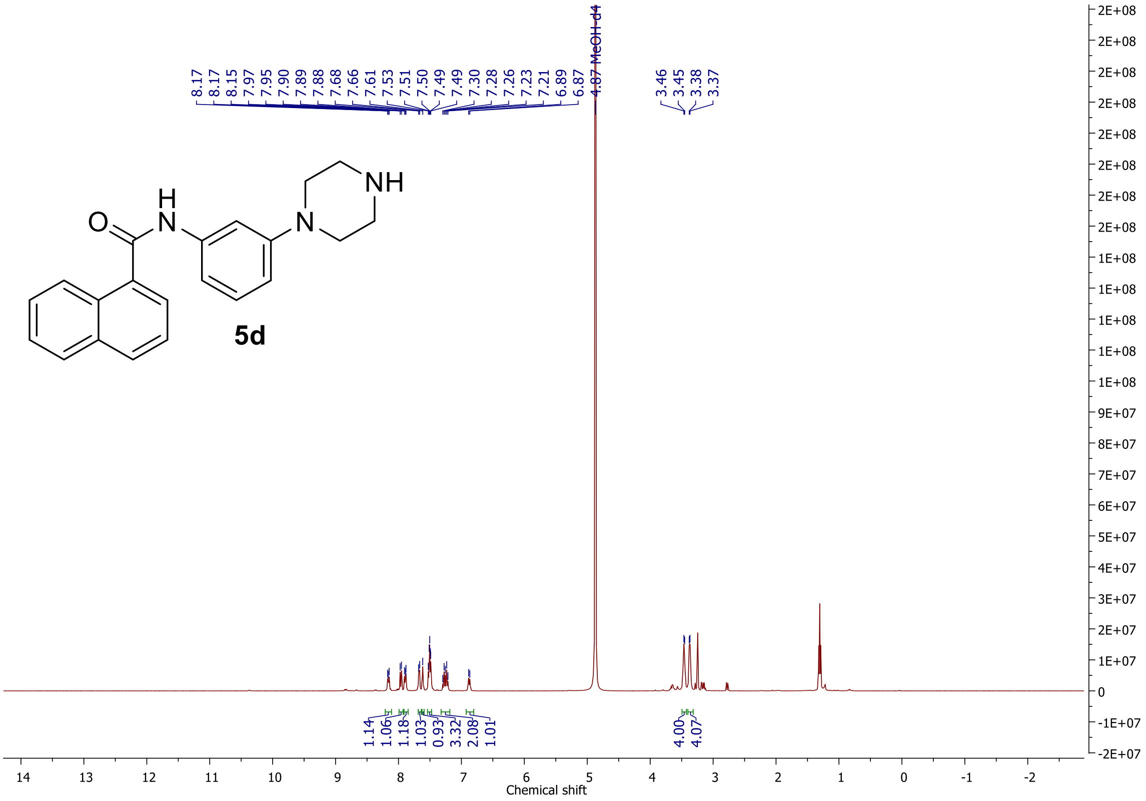

Figure S48. ^{1}H NMR spectrum (MeOH-*d4*, 400 MHz) of *N*-(3-(piperazin-1-yl)phenyl)-1-naphthamide **5d**

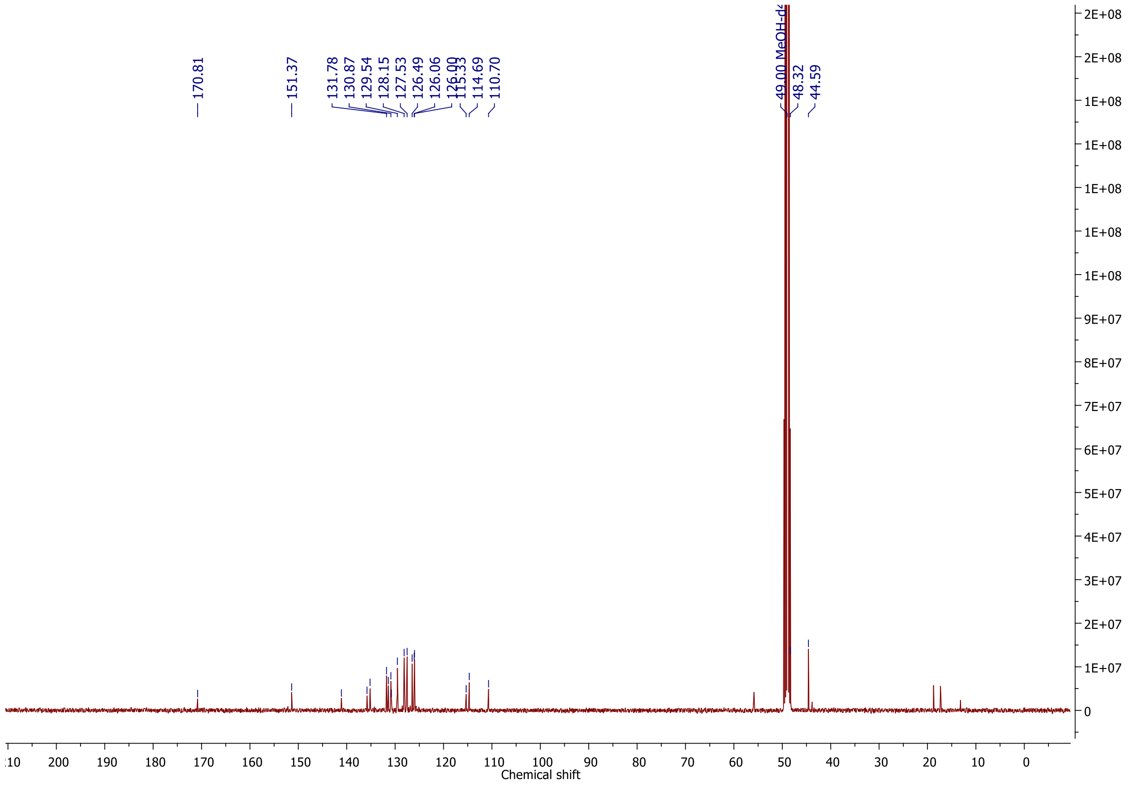

Figure S49. ^{13}C NMR spectrum (MeOH-*d4*, 101 MHz) of *N*-(3-(piperazin-1-yl)phenyl)-1-naphthamide **5d**.

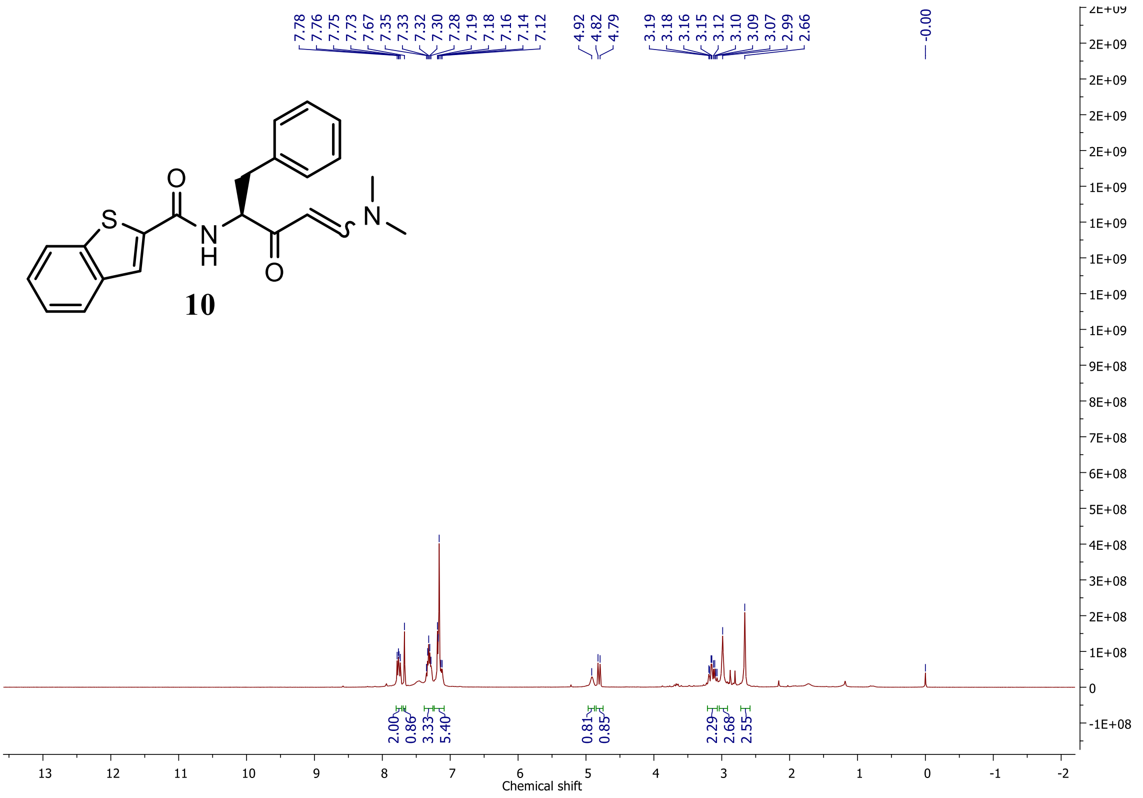

Figure S50. ^{1}H NMR spectrum ($CDCl_3$, 400 MHz) of *(S,E)*-N-(5-(dimethylamino)-3-oxo-1-phenylpent-4-en-2-yl)benzo[b]thiophene-2-carboxamide **10**

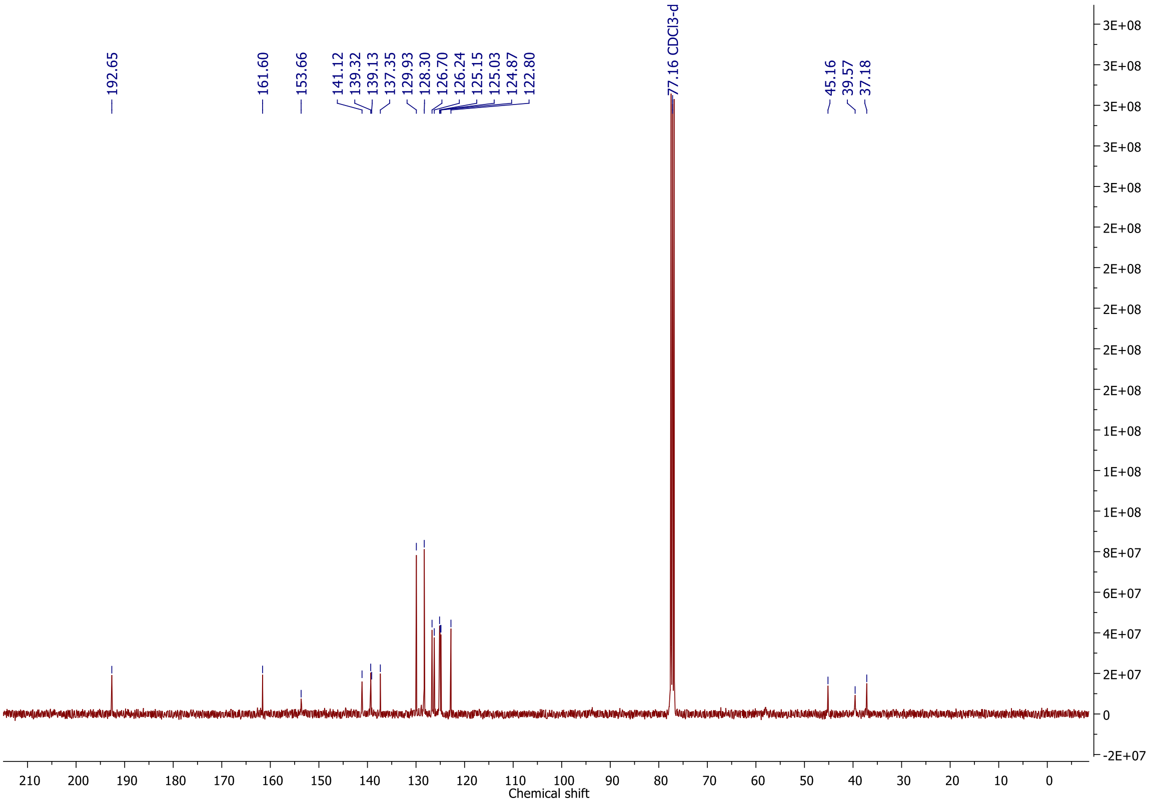

Figure S51. ^{13}C NMR spectrum ($CDCl_3$, 101 MHz) of *(S,E)*-N-(5-(dimethylamino)-3-oxo-1-phenylpent-4-en-2-yl)benzo[b]thiophene-2-carboxamide **10**

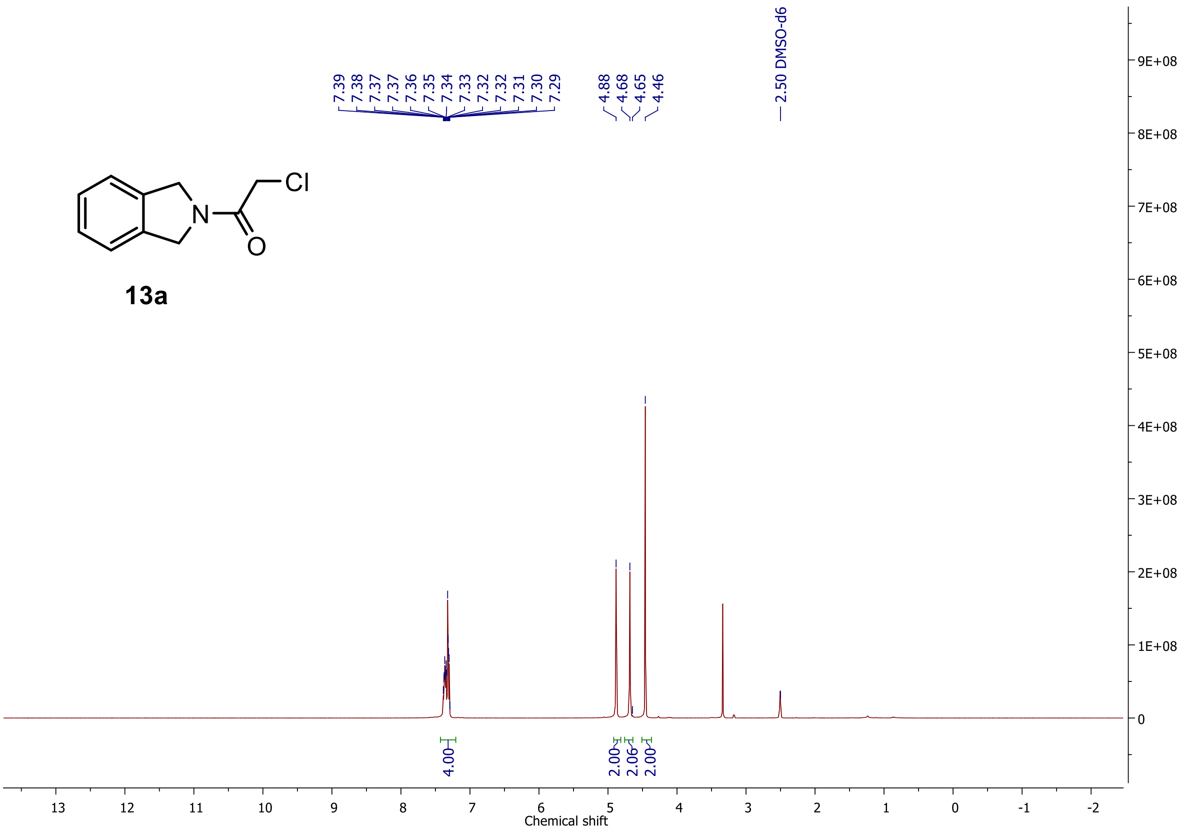

Figure S52. ^{1}H NMR spectrum (DMSO-d_6, 400 MHz) of 2-chloro-1-(isoindolin-2-yl)ethan-1-one **13a**

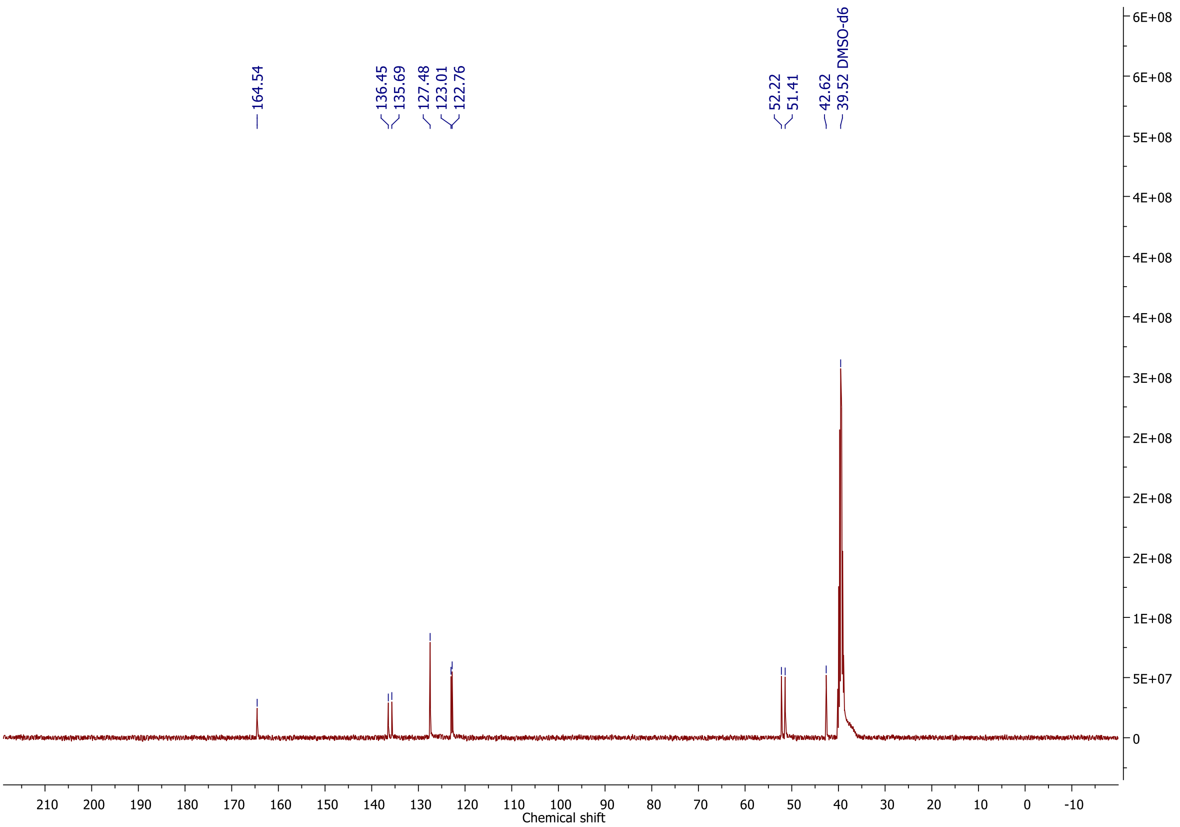

Figure S53. ^{13}C NMR spectrum (DMSO-d_6, 101 MHz) of 2-chloro-1-(isoindolin-2-yl)ethan-1-one **13a**

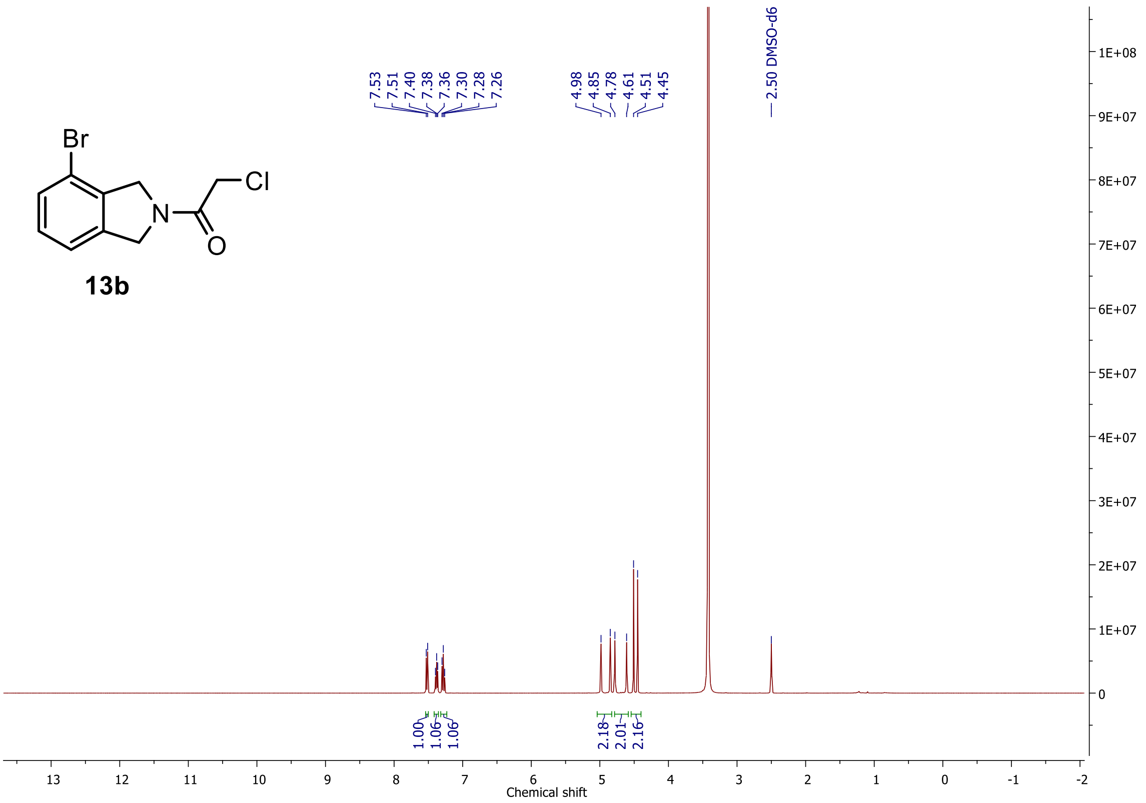

Figure S54. ¹H NMR spectrum (DMSO-d_6, 400 MHz) of 1-(4-bromoisoindolin-2-yl)-2-chloroethan-1-one **13b**

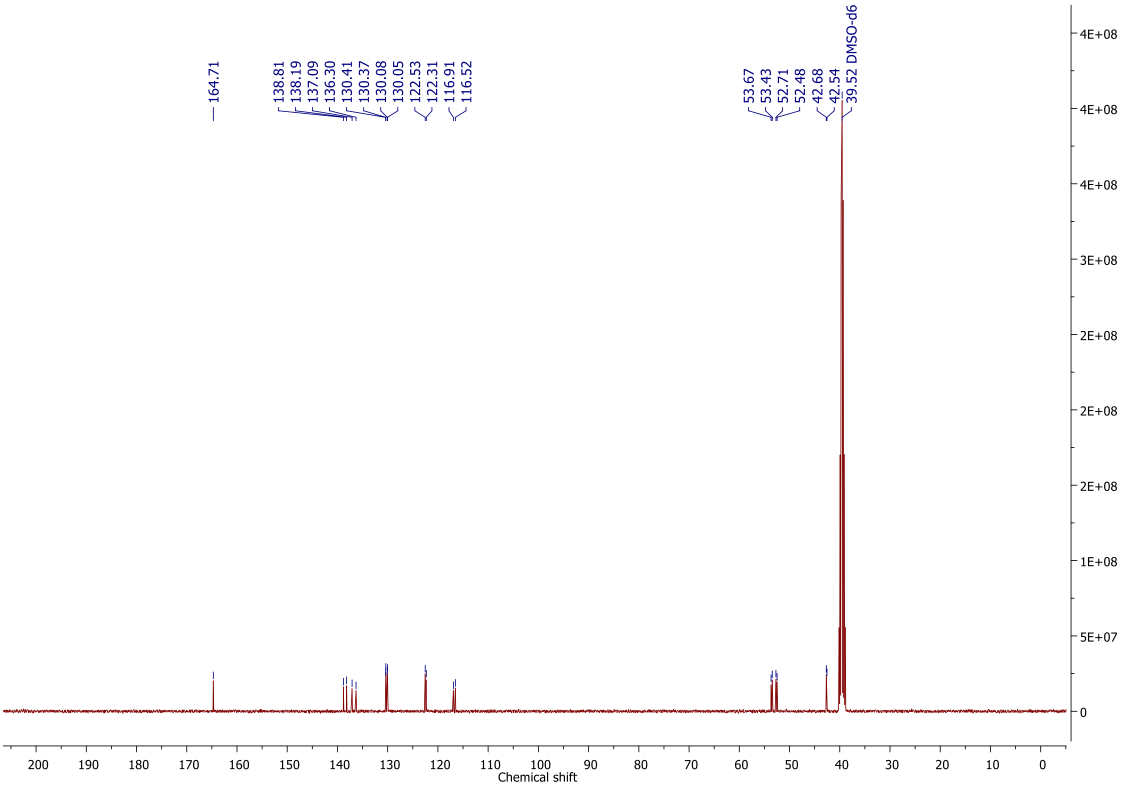

Figure S55. ¹³C NMR spectrum (DMSO-d_6, 101 MHz) of 1-(4-bromoisoindolin-2-yl)-2-chloroethan-1-one **13b**

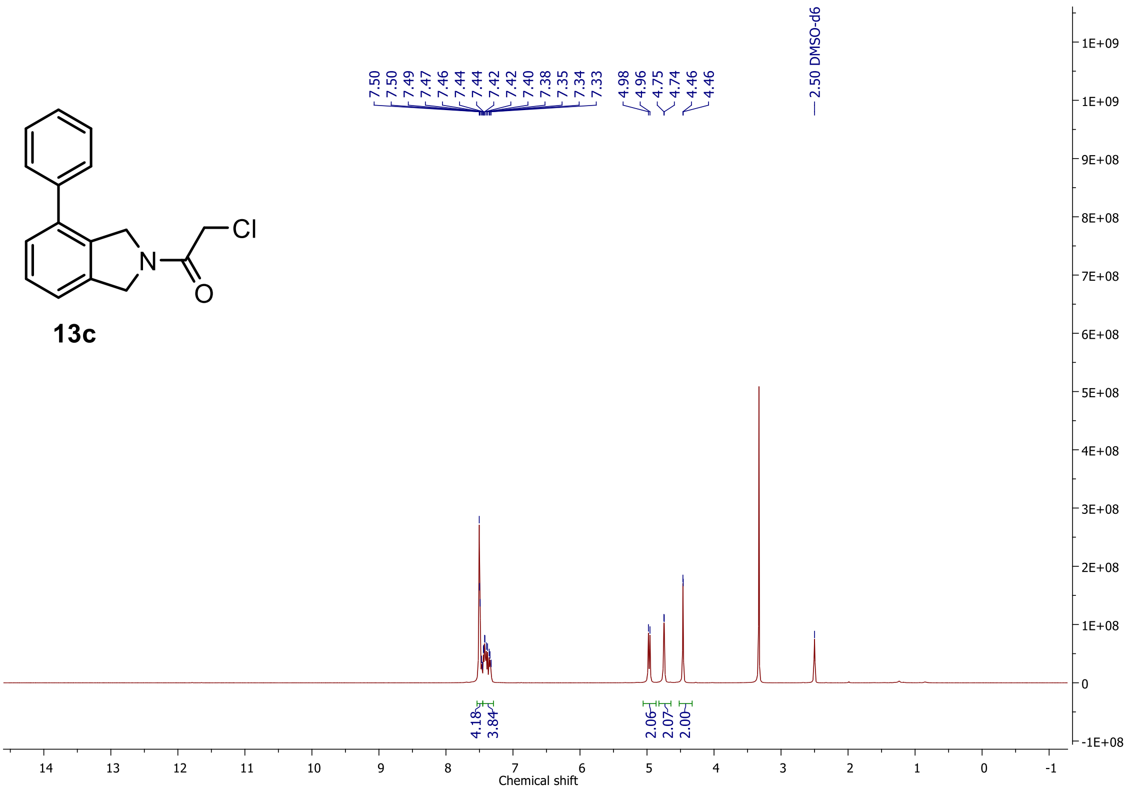

Figure S56. ^{1}H NMR spectrum (DMSO-d_6, 400 MHz) of 2-chloro-1-(4-phenylisoindolin-2-yl)ethan-1-one **13c**

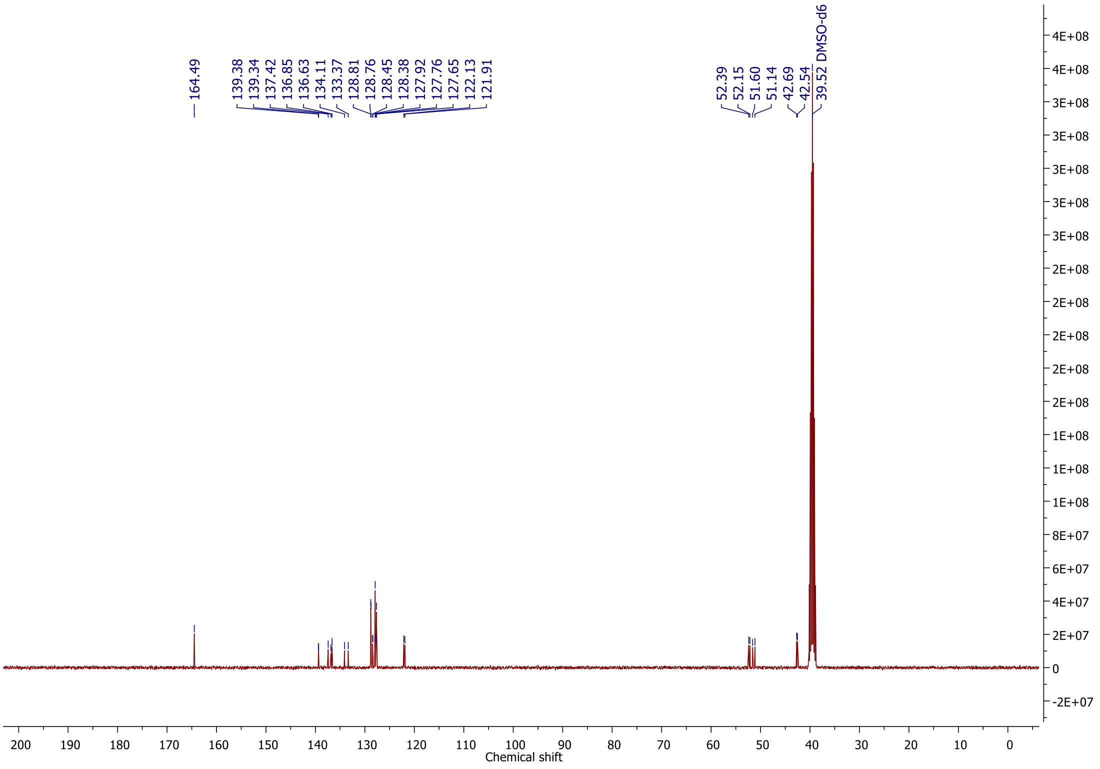

Figure S57. ^{13}C NMR spectrum (DMSO-d_6, 101 MHz) of 2-chloro-1-(4-phenylisoindolin-2-yl)ethan-1-one **13c**

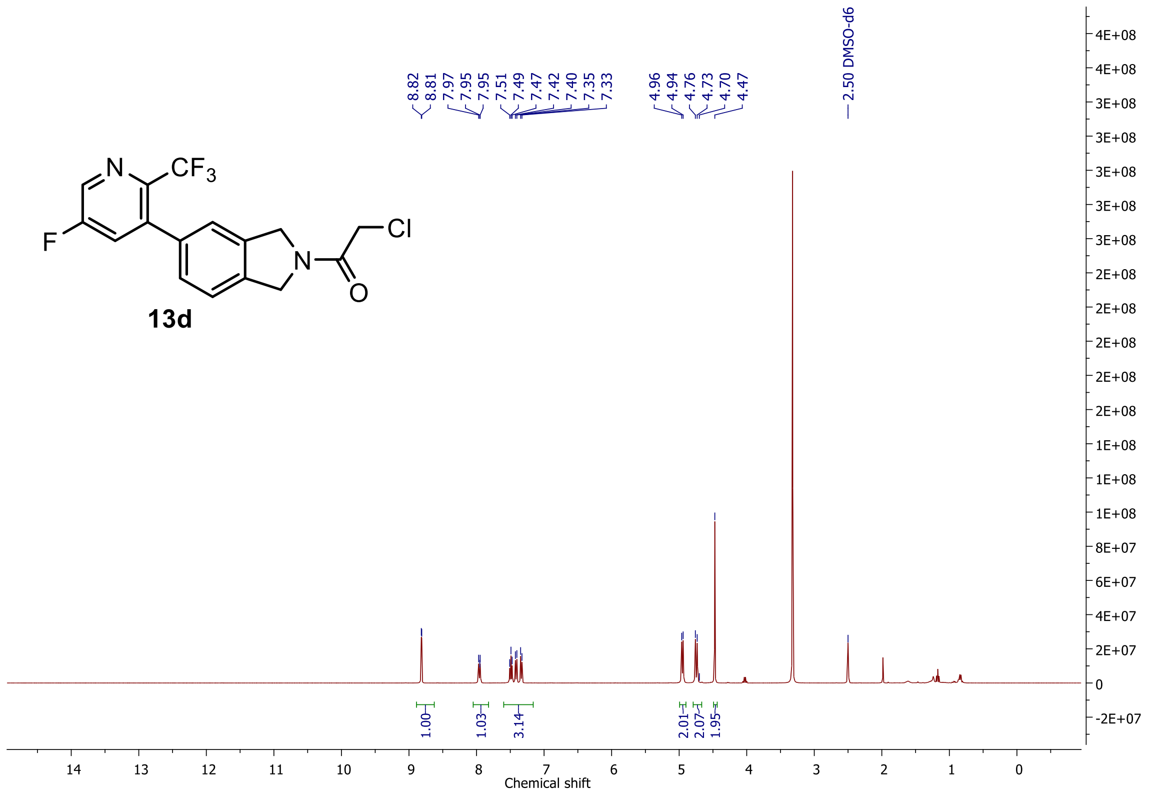

Figure S58. ^{1}H NMR spectrum (DMSO-d_6, 400 MHz) of 2-chloro-1-(5-(5-fluoro-2-(trifluoromethyl)pyridin-3-yl)isoindolin-2-yl)ethan-1-one **13d**

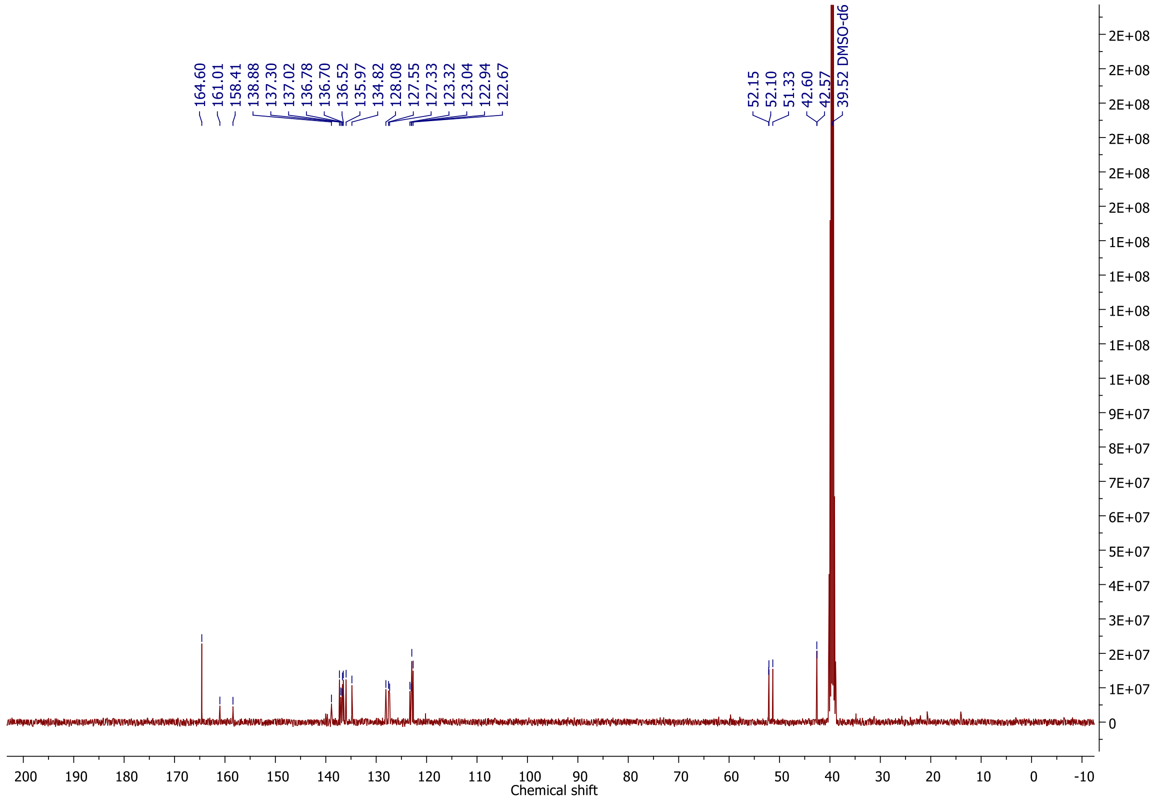

Figure S59. ^{13}C NMR spectrum (DMSO-d_6, 101 MHz) of 2-chloro-1-(5-(5-fluoro-2-(trifluoromethyl)pyridin-3-yl)isoindolin-2-yl)ethan-1-one **13d**

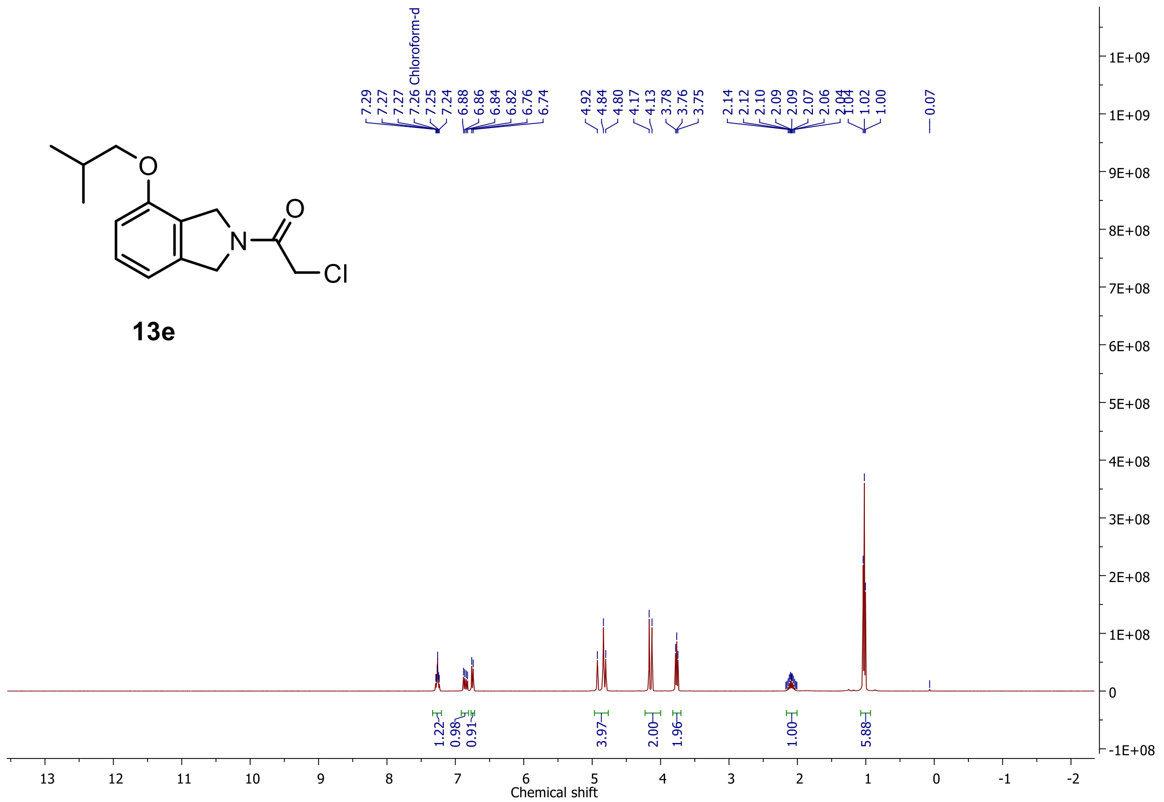

Figure S60. ^{1}H NMR spectrum ($CDCl_3$, 400 MHz) of 2-chloro-1-(4-isobutoxyisoindolin-2-yl)ethan-1-one **13e**

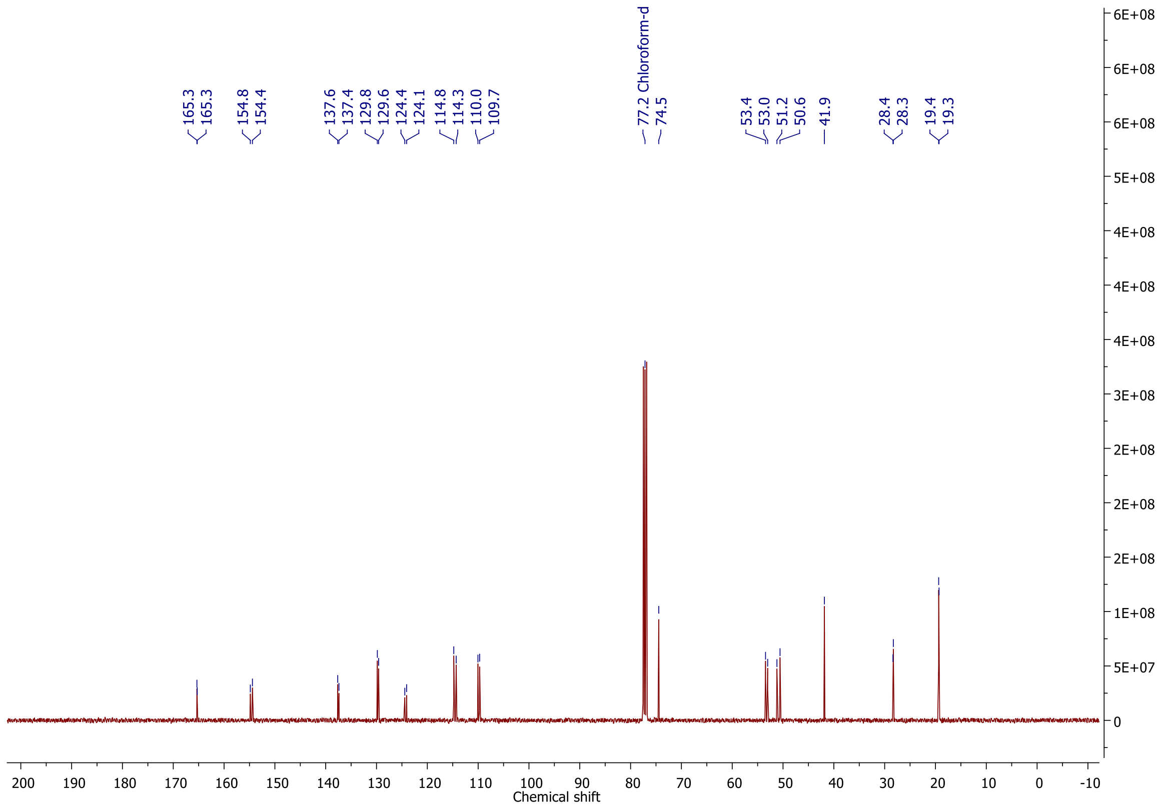

Figure S61. ^{13}C NMR spectrum ($CDCl_3$, 101 MHz) of 2-chloro-1-(4-isobutoxyisoindolin-2-yl)ethan-1-one **13e**

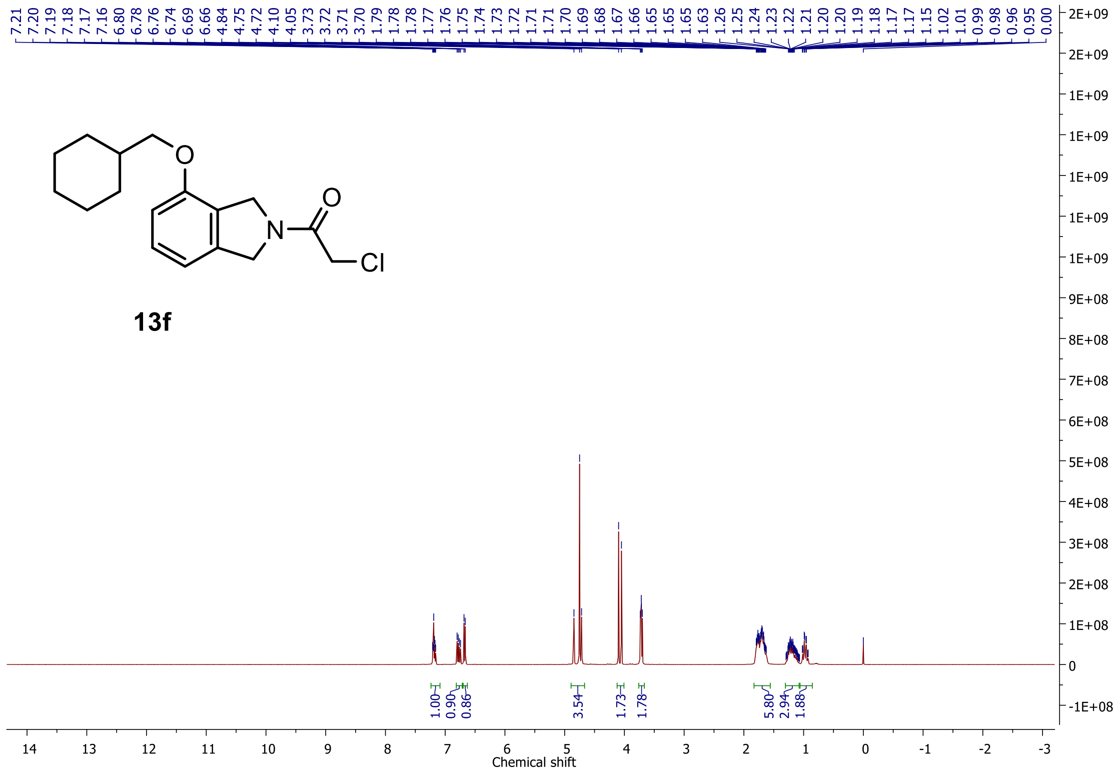

Figure S62. ^{1}H NMR spectrum ($CDCl_3$, 400 MHz) of 2-chloro-1-(4-(cyclohexylmethoxy)isoindolin-2-yl)ethan-1-one **13f**

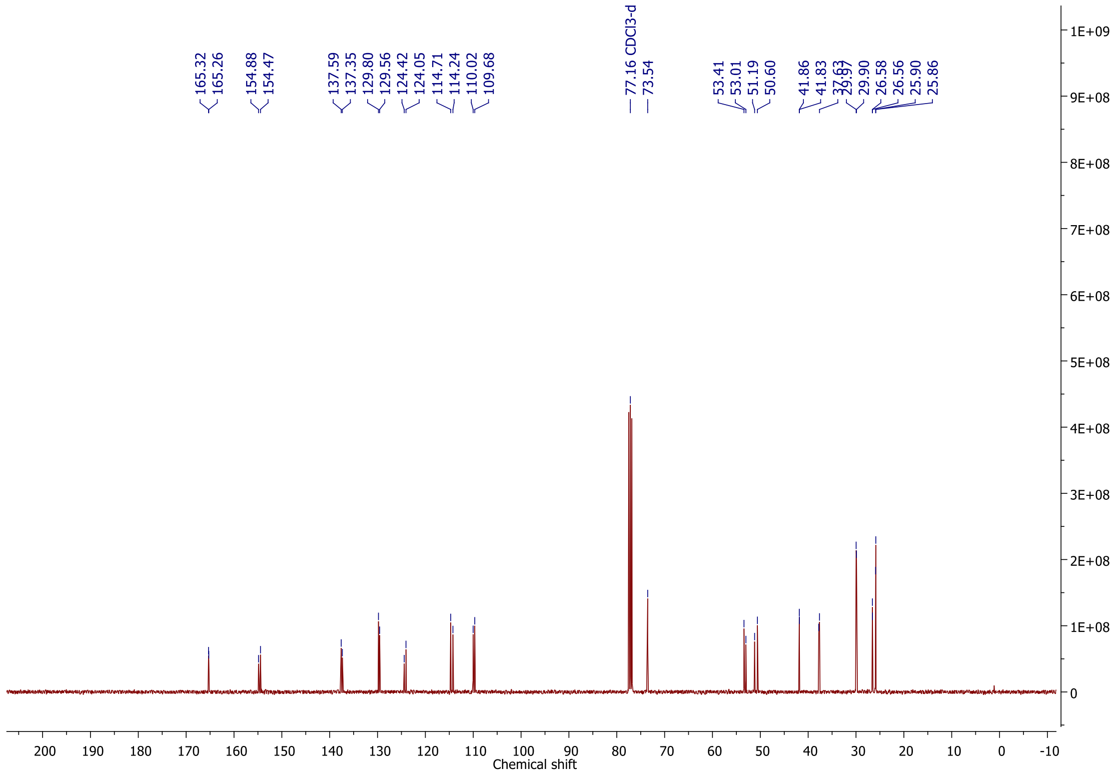

Figure S63. ^{13}C NMR spectrum ($CDCl_3$, 101 MHz) of 2-chloro-1-(4-(cyclohexylmethoxy)isoindolin-2-yl)ethan-1-one **13f**

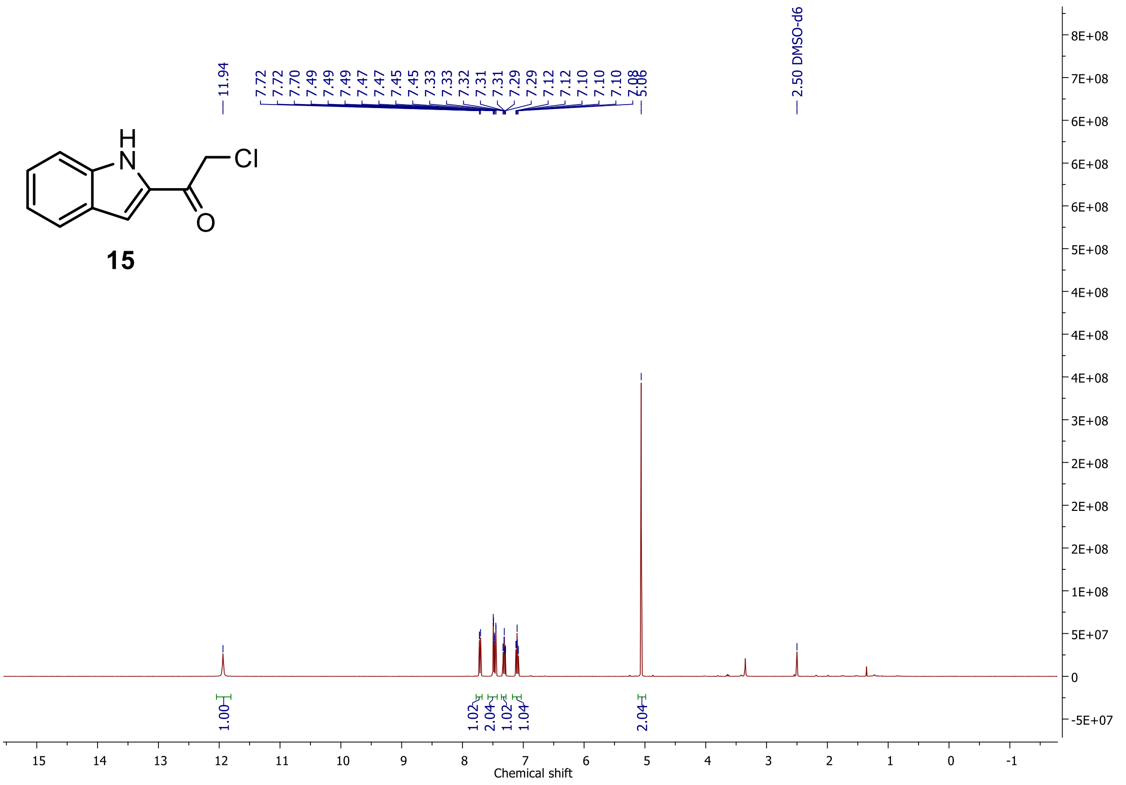

Figure S64. ^{1}H NMR spectrum (DMSO-d_6, 400 MHz) of 2-chloro-1-(1H-indol-2-yl)ethan-1-one **15**

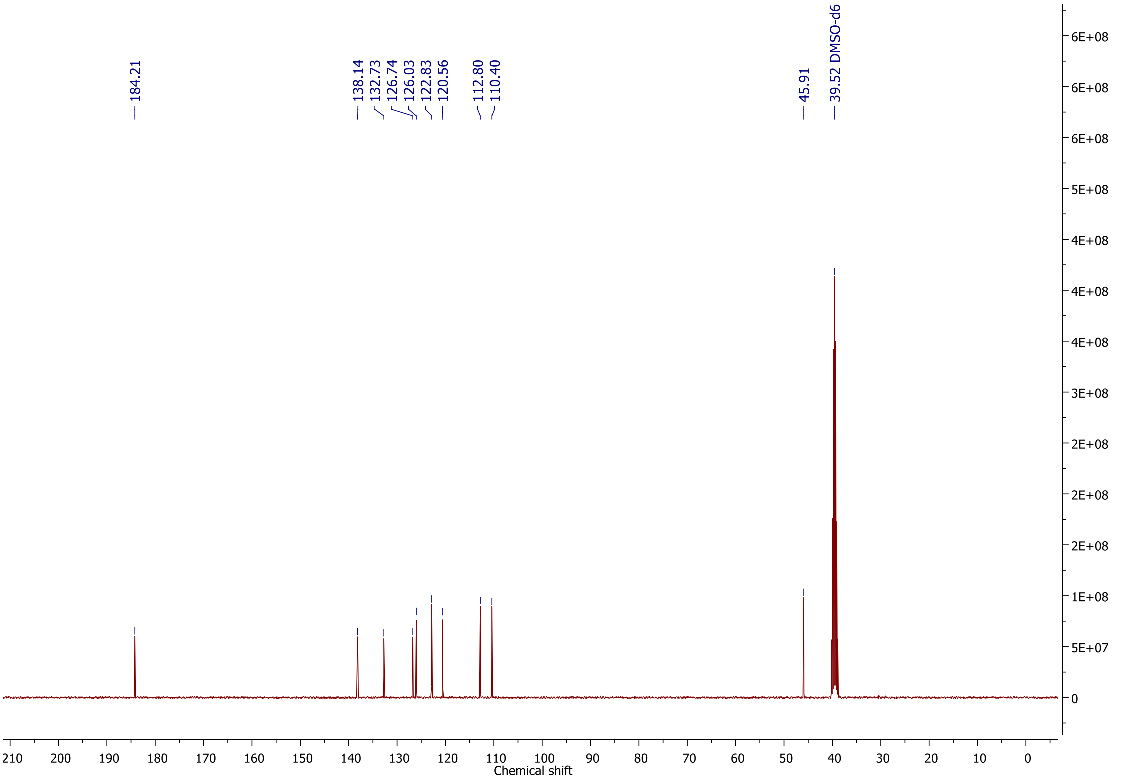

Figure S65. ^{13}C NMR spectrum (DMSO-d_6, 101 MHz) of 2-chloro-1-(1H-indol-2-yl)ethan-1-one **15**

3. HPLC data

Compound 2a

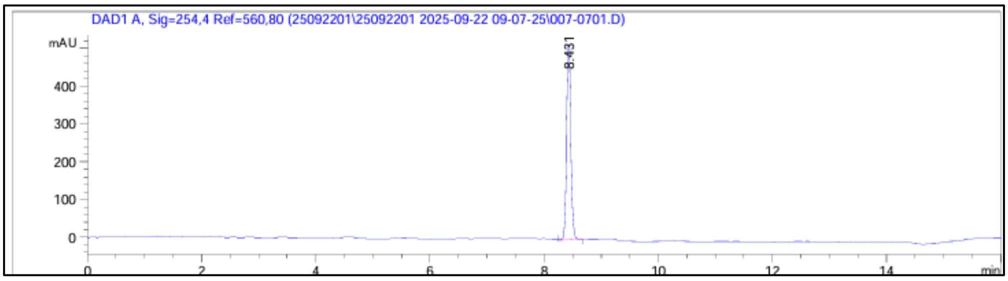

Area Percent Report

Sorted By : Signal
Multiplier: : 1.0000
Dilution: : 1.0000
Use Multiplier & Dilution Factor with ISTDs

Signal 1: DAD1 A, Sig=254,4 Ref=560,80

Peak #	RetTime [min]	Type	Width [min]	Area [mAU*s]	Height [mAU]	Area %
1	8.431	BB	0.0799	2649.74023	516.46881	100.0000

Compound 2b

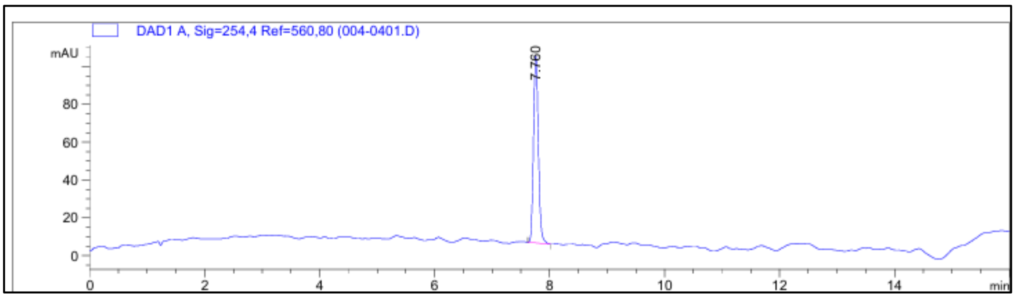

Area Percent Report

Sorted By : Signal
Multiplier: : 1.0000
Dilution: : 1.0000
Use Multiplier & Dilution Factor with ISTDs

Signal 1: DAD1 A, Sig=254,4 Ref=560,80

Peak #	RetTime [min]	Type	Width [min]	Area [mAU*s]	Height [mAU]	Area %
1	7.760	BB	0.0907	583.21228	99.00910	100.0000

Compound 2c

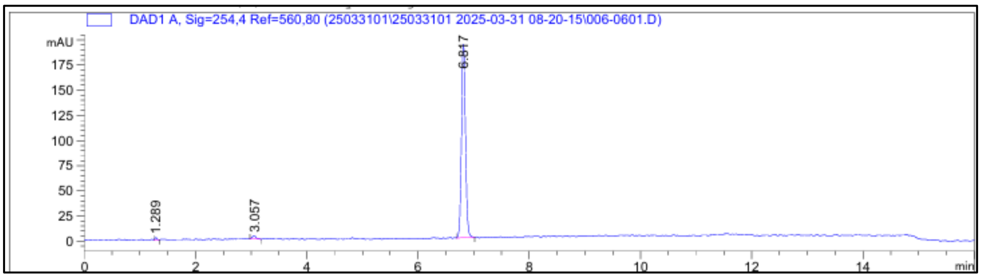

Area Percent Report

Sorted By : Signal
Multiplier: : 1.0000
Dilution: : 1.0000
Use Multiplier & Dilution Factor with ISTDs

Signal 1: DAD1 A, Sig=254,4 Ref=560,80

Peak #	RetTime [min]	Type	Width [min]	Area [mAU*s]	Height [mAU]	Area %
1	1.289	BB	0.0414	5.56369	2.20999	0.5741
2	3.057	BB	0.0812	14.55402	2.96596	1.5017
3	6.817	BB	0.0775	949.04883	192.70551	97.9242

Compound 2d

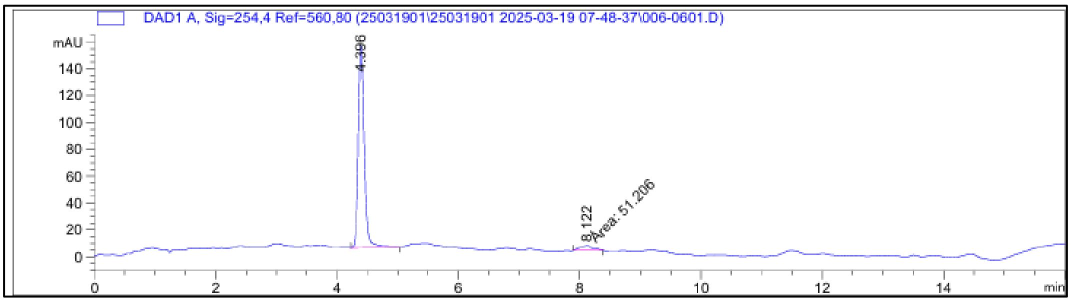

Area Percent Report

Sorted By : Signal
Multiplier: : 1.0000
Dilution: : 1.0000
Use Multiplier & Dilution Factor with ISTDs

Signal 1: DAD1 A, Sig=254,4 Ref=560,80

Peak #	RetTime [min]	Type	Width [min]	Area [mAU*s]	Height [mAU]	Area %
1	4.396	BB	0.1029	1020.83905	151.02742	95.2235
2	8.122	MM	0.2599	51.20603	3.28343	4.7765

Compound 2e

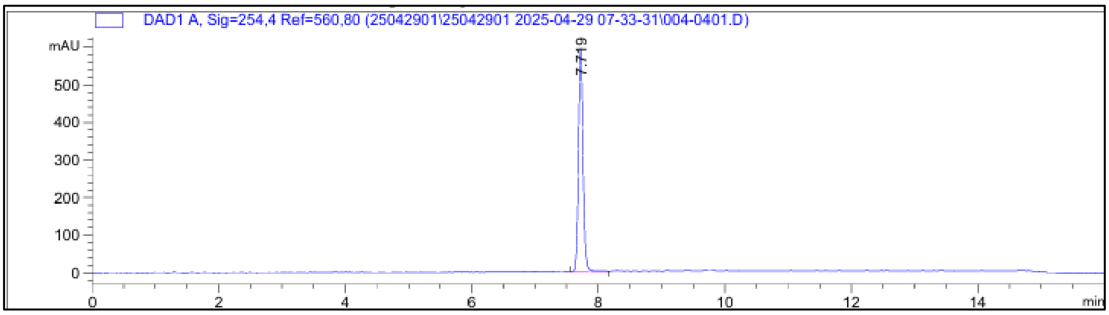

==

Area Percent Report

==

```
Sorted By              :      Signal
Multiplier:                   :       1.0000
Dilution:                     :       1.0000
Use Multiplier & Dilution Factor with ISTDs
```

Signal 1: DAD1 A, Sig=254,4 Ref=560,80

Peak #	RetTime [min]	Type	Width [min]	Area [mAU*s]	Height [mAU]	Area %
1	7.719	BB	0.0770	3003.19141	593.61530	100.0000

Compound 2f

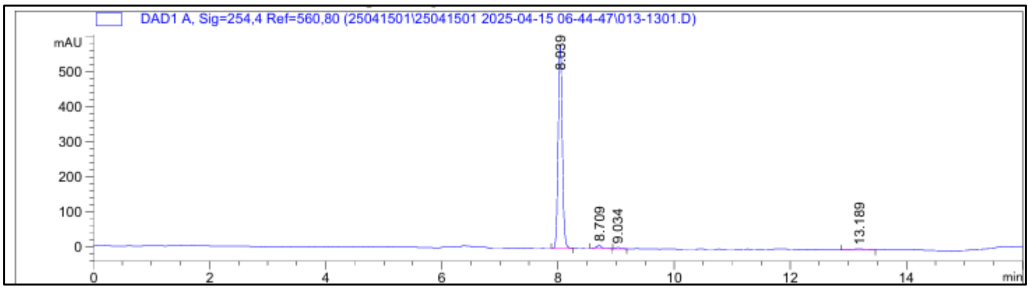

==

Area Percent Report

==

```
Sorted By              :      Signal
Multiplier:                   :       1.0000
Dilution:                     :       1.0000
Use Multiplier & Dilution Factor with ISTDs
```

Signal 1: DAD1 A, Sig=254,4 Ref=560,80

Peak #	RetTime [min]	Type	Width [min]	Area [mAU*s]	Height [mAU]	Area %
1	8.039	BB	0.0778	2873.31665	579.98462	95.8560
2	8.709	BB	0.0880	55.10960	9.18207	1.8385
3	9.034	BB	0.0847	20.71635	3.73416	0.6911
4	13.189	BB	0.2022	48.39191	3.48140	1.6144

Compound 2g

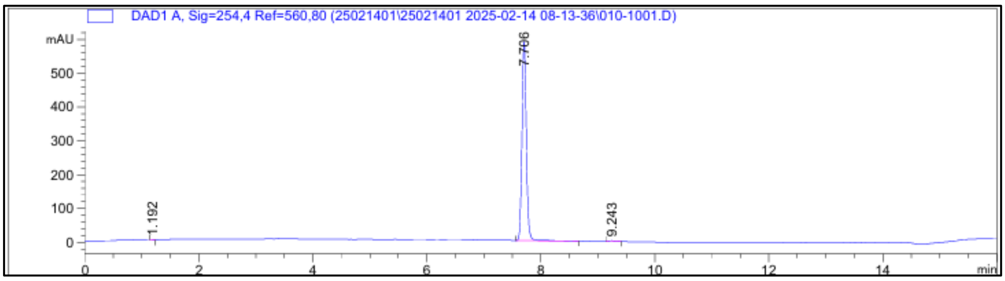


```
=====================================================================
                         Area Percent Report
=====================================================================

Sorted By             :      Signal
Multiplier:                  :      1.0000
Dilution:                    :      1.0000
Use Multiplier & Dilution Factor with ISTDs


Signal 1: DAD1 A, Sig=254,4 Ref=560,80
```

Peak #	RetTime [min]	Type	Width [min]	Area [mAU*s]	Height [mAU]	Area %
1	1.192	BB	0.0451	5.10998	1.70018	0.1629
2	7.706	BB	0.0818	3118.58057	588.93805	99.3998
3	9.243	BB	0.0814	13.72128	2.52461	0.4373

Compound 2h

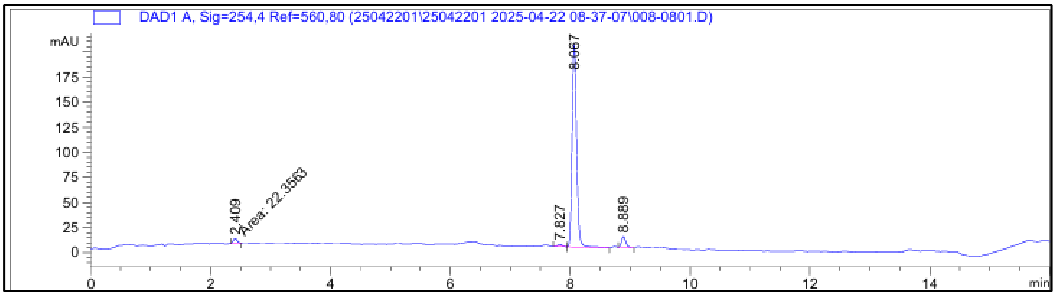


```
=====================================================================
                         Area Percent Report
=====================================================================

Sorted By             :      Signal
Multiplier:                  :      1.0000
Dilution:                    :      1.0000
Use Multiplier & Dilution Factor with ISTDs


Signal 1: DAD1 A, Sig=254,4 Ref=560,80
```

Peak #	RetTime [min]	Type	Width [min]	Area [mAU*s]	Height [mAU]	Area %
1	2.409	MM	0.0781	22.35626	4.77058	1.9494
2	7.827	BB	0.0828	12.25033	2.27626	1.0682
3	8.067	BB	0.0790	1056.61646	201.97330	92.1317
4	8.889	VB	0.0797	55.63163	10.86366	4.8508

Compound 2i

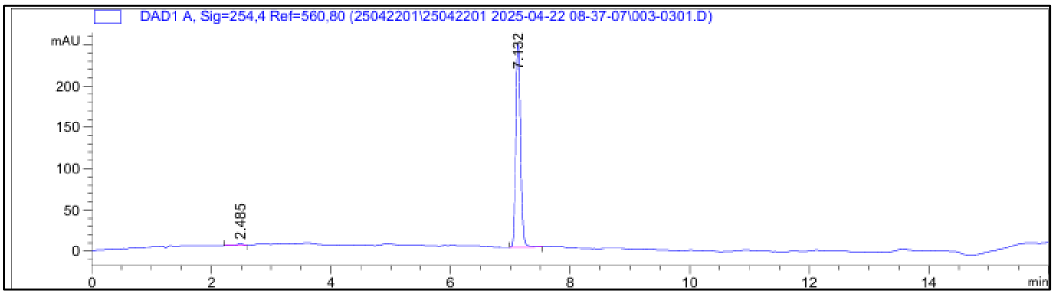


```
=====================================================================
                         Area Percent Report
=====================================================================

Sorted By             :      Signal
Multiplier:                  :       1.0000
Dilution:                    :       1.0000
Use Multiplier & Dilution Factor with ISTDs


Signal 1: DAD1 A, Sig=254,4 Ref=560,80

Peak RetTime Type  Width     Area       Height     Area
  #   [min]        [min]   [mAU*s]     [mAU]        %
----|-------|----|-------|----------|----------|--------|
   1   2.485 BB    0.0980   13.89077    2.07658   1.0199
   2   7.132 BB    0.0837 1348.10974  247.05472  98.9801
```

Compound 2j

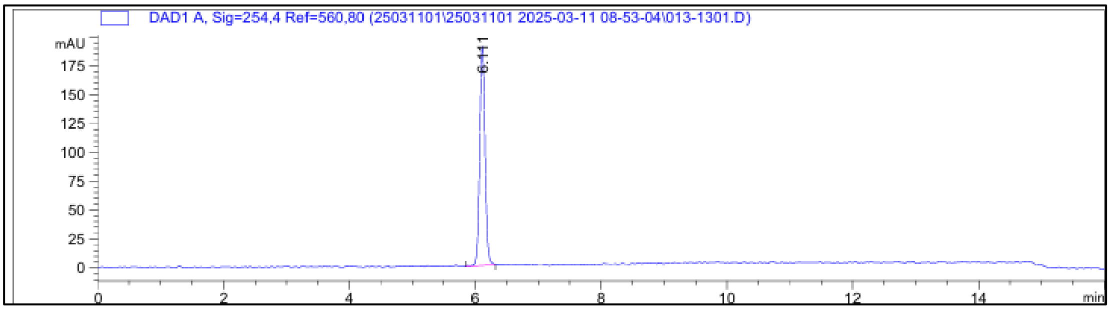


```
=====================================================================
                         Area Percent Report
=====================================================================

Sorted By             :      Signal
Multiplier:                  :       1.0000
Dilution:                    :       1.0000
Use Multiplier & Dilution Factor with ISTDs


Signal 1: DAD1 A, Sig=254,4 Ref=560,80

Peak RetTime Type  Width     Area       Height     Area
  #   [min]        [min]   [mAU*s]     [mAU]        %
----|-------|----|-------|----------|----------|--------|
   1   6.111 BB    0.0928 1121.78076  190.22624 100.0000
```

Compound 2k

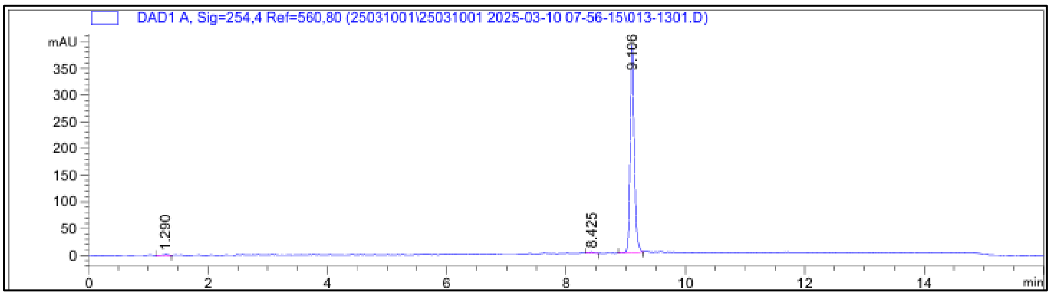

Area Percent Report

Sorted By : Signal
Multiplier: : 1.0000
Dilution: : 1.0000
Use Multiplier & Dilution Factor with ISTDs

Signal 1: DAD1 A, Sig=254,4 Ref=560,80

Peak #	RetTime [min]	Type	Width [min]	Area [mAU*s]	Height [mAU]	Area %
1	1.290	BB	0.0822	13.65527	2.26818	0.7158
2	8.425	BB	0.0690	11.23257	2.29797	0.5888
3	9.106	BB	0.0744	1882.90173	389.37674	98.6955

Compound 2l

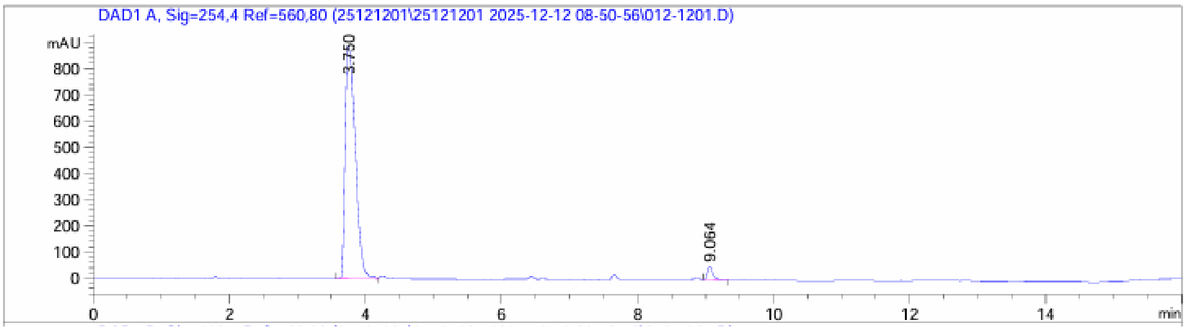

Area Percent Report

Sorted By : Signal
Multiplier: : 1.0000
Dilution: : 1.0000
Use Multiplier & Dilution Factor with ISTDs

Signal 1: DAD1 A, Sig=254,4 Ref=560,80

Peak #	RetTime [min]	Type	Width [min]	Area [mAU*s]	Height [mAU]	Area %
1	3.750	BV	0.1674	9246.71777	888.95404	97.0083
2	9.064	VV	0.0850	285.16812	51.21119	2.9917

Compound 2m

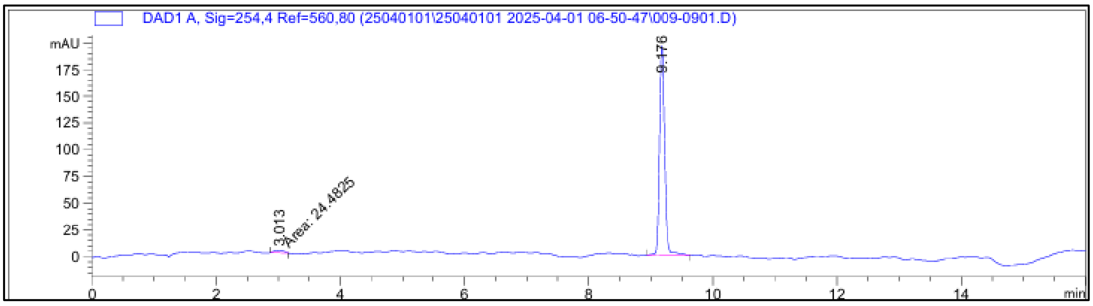


```
=====================================================================
                         Area Percent Report
=====================================================================

Sorted By             :      Signal
Multiplier:                  :      1.0000
Dilution:                    :      1.0000
Use Multiplier & Dilution Factor with ISTDs


Signal 1: DAD1 A, Sig=254,4 Ref=560,80

Peak RetTime Type  Width     Area      Height     Area
  #   [min]        [min]   [mAU*s]     [mAU]       %
----|-------|----|-------|----------|----------|--------|
   1   3.013 MM    0.1825   24.48246    2.23644   2.1150
   2   9.176 BB    0.0874 1133.06909  196.09114  97.8850
```

Compound 2n

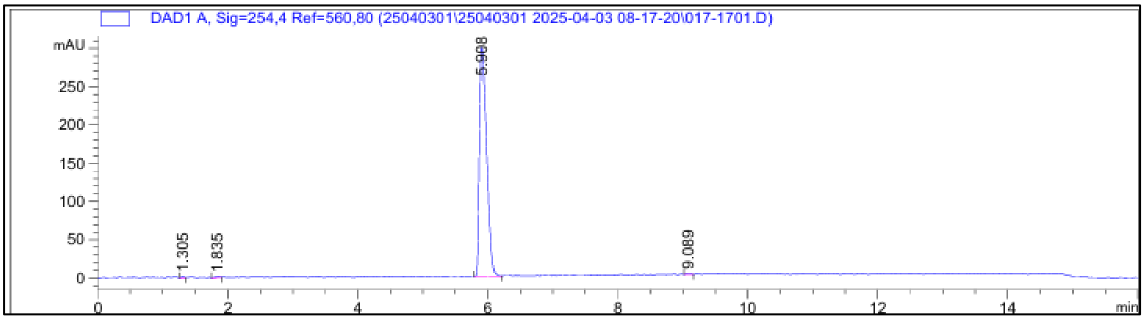


```
=====================================================================
                         Area Percent Report
=====================================================================

Sorted By             :      Signal
Multiplier:                  :      1.0000
Dilution:                    :      1.0000
Use Multiplier & Dilution Factor with ISTDs


Signal 1: DAD1 A, Sig=254,4 Ref=560,80

Peak RetTime Type  Width     Area      Height     Area
  #   [min]        [min]   [mAU*s]     [mAU]       %
----|-------|----|-------|----------|----------|--------|
   1   1.305 BB    0.0428    5.15453    1.95070   0.2276
   2   1.835 BB    0.0616    5.29476    1.11159   0.2337
   3   5.908 BV    0.1176 2248.37036  299.54123  99.2568
   4   9.089 BB    0.0766    6.38632    1.41576   0.2819
```

Compound 2o

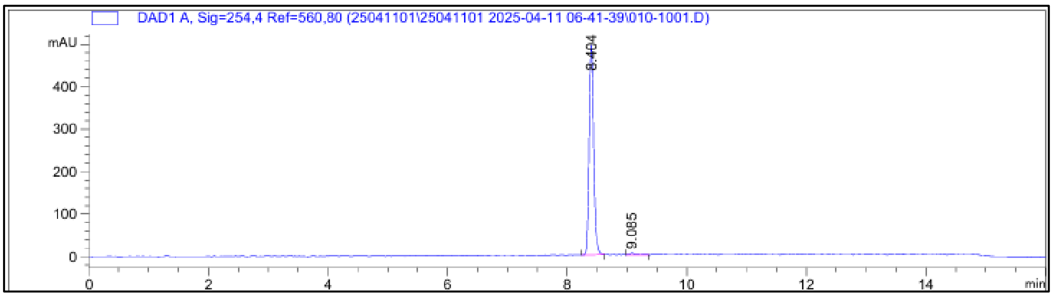


```
=====================================================================
                     Area Percent Report
=====================================================================

Sorted By             :      Signal
Multiplier:                  :      1.0000
Dilution:                    :      1.0000
Use Multiplier & Dilution Factor with ISTDs


Signal 1: DAD1 A, Sig=254,4 Ref=560,80
```

Peak #	RetTime [min]	Type	Width [min]	Area [mAU*s]	Height [mAU]	Area %
1	8.404	BB	0.0788	2502.52051	496.15714	98.9453
2	9.085	BB	0.0981	26.67437	3.78869	1.0547

Compound 2p

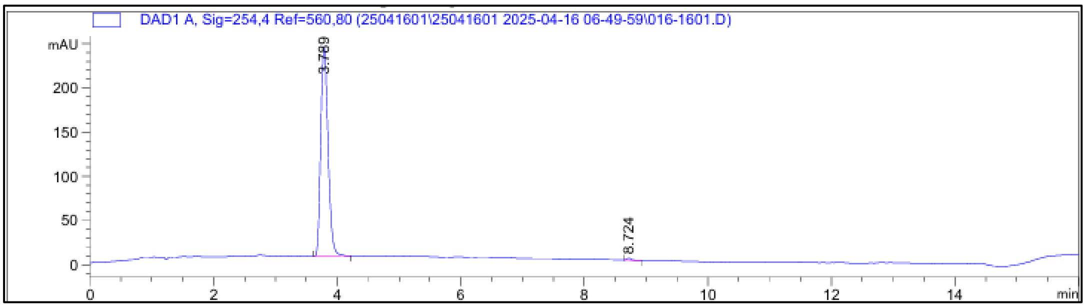


```
=====================================================================
                     Area Percent Report
=====================================================================

Sorted By             :      Signal
Multiplier:                  :      1.0000
Dilution:                    :      1.0000
Use Multiplier & Dilution Factor with ISTDs


Signal 1: DAD1 A, Sig=254,4 Ref=560,80
```

Peak #	RetTime [min]	Type	Width [min]	Area [mAU*s]	Height [mAU]	Area %
1	3.789	BB	0.1273	1930.73584	236.47511	99.4151
2	8.724	BB	0.0828	11.35921	2.11186	0.5849

Compound 2q

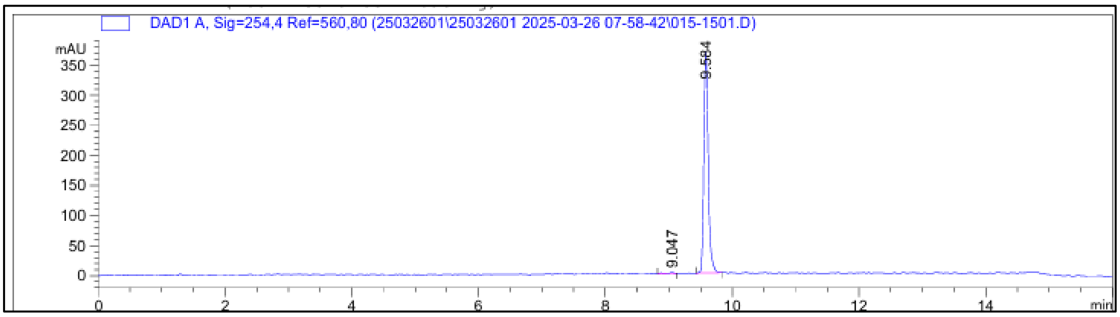

===
Area Percent Report
===

Sorted By : Signal
Multiplier: : 1.0000
Dilution: : 1.0000
Use Multiplier & Dilution Factor with ISTDs

Signal 1: DAD1 A, Sig=254,4 Ref=560,80

Peak #	RetTime [min]	Type	Width [min]	Area [mAU*s]	Height [mAU]	Area %
1	9.047	BB	0.0884	11.79218	1.85018	0.6623
2	9.584	BB	0.0719	1768.76440	368.87231	99.3377

Compound 2r

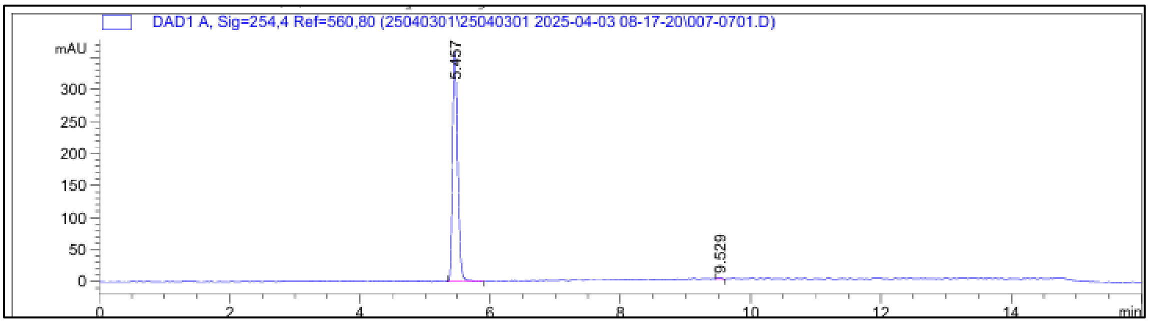

===
Area Percent Report
===

Sorted By : Signal
Multiplier: : 1.0000
Dilution: : 1.0000
Use Multiplier & Dilution Factor with ISTDs

Signal 1: DAD1 A, Sig=254,4 Ref=560,80

Peak #	RetTime [min]	Type	Width [min]	Area [mAU*s]	Height [mAU]	Area %
1	5.457	VB	0.0829	1943.77393	360.40912	99.6381
2	9.529	BB	0.0573	7.06062	1.81687	0.3619

Compound 2s

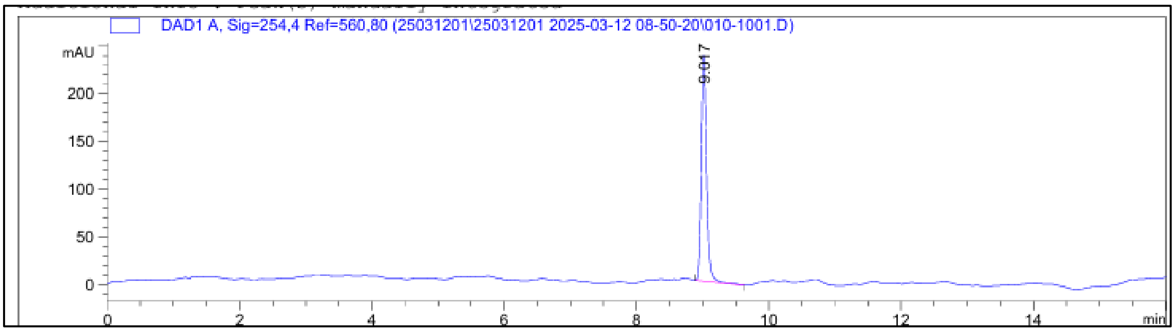

==

Area Percent Report

==

Sorted By : Signal
Multiplier: : 1.0000
Dilution: : 1.0000
Use Multiplier & Dilution Factor with ISTDs

Signal 1: DAD1 A, Sig=254,4 Ref=560,80

Peak #	RetTime [min]	Type	Width [min]	Area [mAU*s]	Height [mAU]	Area %
1	9.017	BB	0.0844	1313.69580	238.01314	100.0000

Compound 2t

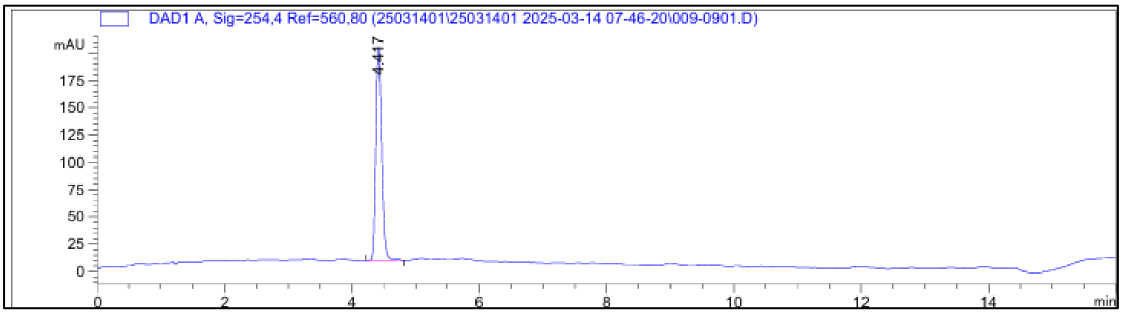

==

Area Percent Report

==

Sorted By : Signal
Multiplier: : 1.0000
Dilution: : 1.0000
Use Multiplier & Dilution Factor with ISTDs

Signal 1: DAD1 A, Sig=254,4 Ref=560,80

Peak #	RetTime [min]	Type	Width [min]	Area [mAU*s]	Height [mAU]	Area %
1	4.417	BB	0.0968	1222.40625	195.90395	100.0000

Compound 5a

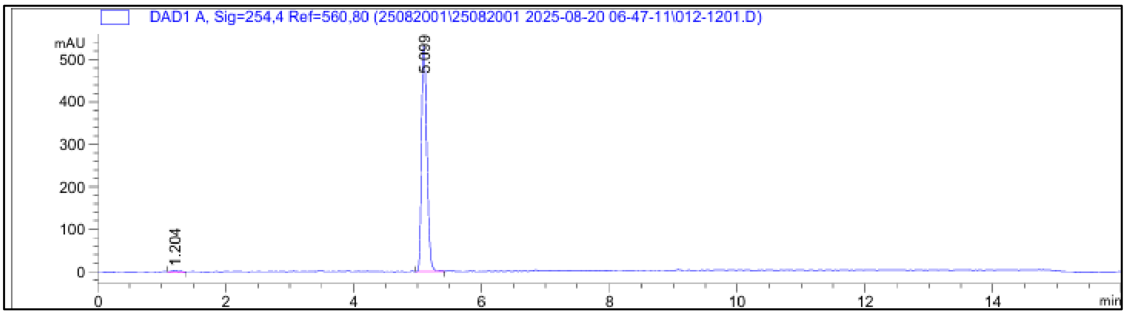

==

Area Percent Report

==

Sorted By : Signal
Multiplier: : 1.0000
Dilution: : 1.0000
Use Multiplier & Dilution Factor with ISTDs

Signal 1: DAD1 A, Sig=254,4 Ref=560,80

Peak #	RetTime [min]	Type	Width [min]	Area [mAU*s]	Height [mAU]	Area %
1	1.204	BB	0.0843	21.01057	3.69805	0.6924
2	5.099	BB	0.0880	3013.63843	532.61414	99.3076

Compound 5b

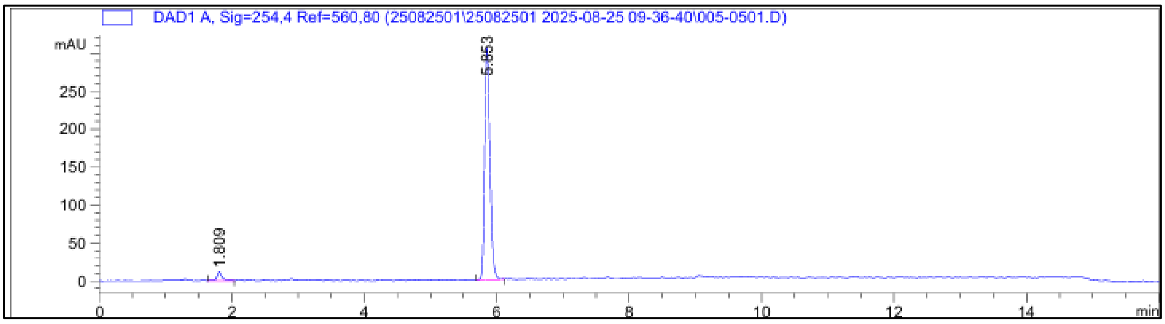

==

Area Percent Report

==

Sorted By : Signal
Multiplier: : 1.0000
Dilution: : 1.0000
Use Multiplier & Dilution Factor with ISTDs

Signal 1: DAD1 A, Sig=254,4 Ref=560,80

Peak #	RetTime [min]	Type	Width [min]	Area [mAU*s]	Height [mAU]	Area %
1	1.809	BB	0.0807	63.71040	11.48189	3.6448
2	5.853	BB	0.0862	1684.28284	306.23120	96.3552

Compound 5c

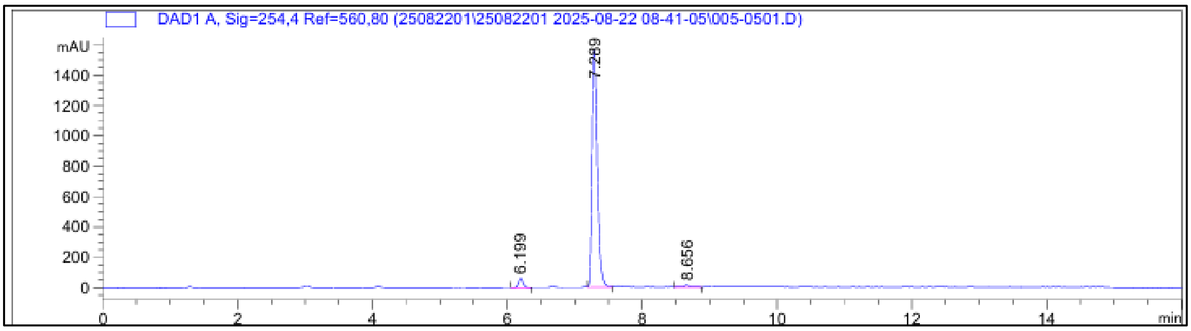


```
=====================================================================
                         Area Percent Report
=====================================================================

Sorted By             :      Signal
Multiplier:                   :      1.0000
Dilution:                     :      1.0000
Use Multiplier & Dilution Factor with ISTDs


Signal 1: DAD1 A, Sig=254,4 Ref=560,80
```

Peak #	RetTime [min]	Type	Width [min]	Area [mAU*s]	Height [mAU]	Area %
1	6.199	BB	0.0707	265.67923	58.86242	2.9773
2	7.289	VV	0.0838	8580.32422	1569.20605	96.1548
3	8.656	VB	0.0821	77.44819	13.67719	0.8679

Compound 5d

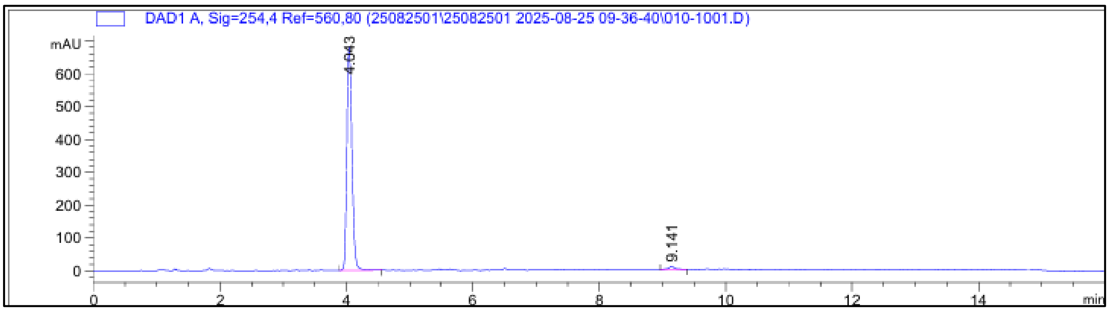


```
=====================================================================
                         Area Percent Report
=====================================================================

Sorted By             :      Signal
Multiplier:                   :      1.0000
Dilution:                     :      1.0000
Use Multiplier & Dilution Factor with ISTDs


Signal 1: DAD1 A, Sig=254,4 Ref=560,80
```

Peak #	RetTime [min]	Type	Width [min]	Area [mAU*s]	Height [mAU]	Area %
1	4.043	BB	0.0884	3880.44873	681.83142	98.2704
2	9.141	BB	0.0977	68.29834	10.26120	1.7296

Compound 10

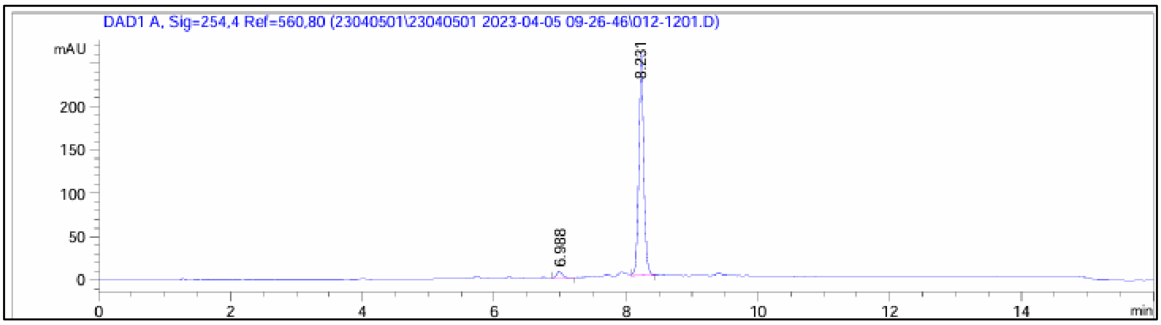


```
==================================================================
                    Area Percent Report
==================================================================

Sorted By             :      Signal
Multiplier:                  :      1.0000
Dilution:                    :      1.0000
Use Multiplier & Dilution Factor with ISTDs


Signal 1: DAD1 A, Sig=254,4 Ref=560,80

Peak RetTime Type  Width     Area      Height     Area
  #   [min]        [min]   [mAU*s]     [mAU]        %
----|-------|----|-------|----------|----------|--------|
   1   6.988 BB    0.0832   38.33504    7.07458   2.7548
   2   8.231 VB    0.0791 1353.23389  258.50446  97.2452
```

Compound 13a

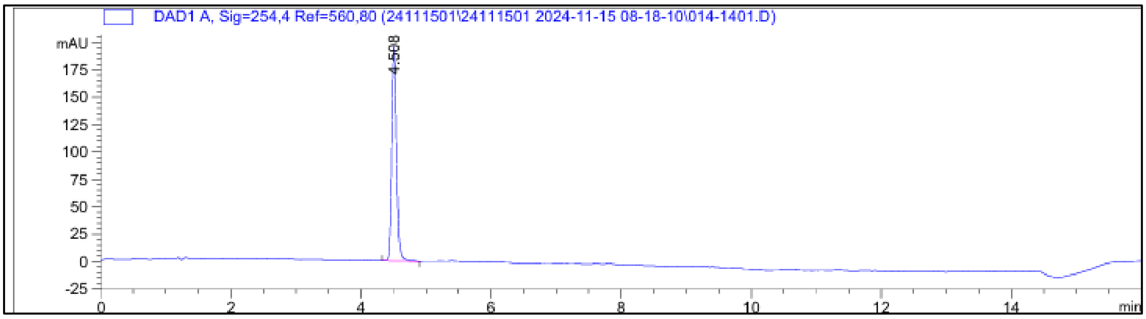


```
==================================================================
                    Area Percent Report
==================================================================

Sorted By             :      Signal
Multiplier:                  :      1.0000
Dilution:                    :      1.0000
Use Multiplier & Dilution Factor with ISTDs


Signal 1: DAD1 A, Sig=254,4 Ref=560,80

Peak RetTime Type  Width     Area      Height     Area
  #   [min]        [min]   [mAU*s]     [mAU]        %
----|-------|----|-------|----------|----------|--------|
   1   4.508 BB    0.0781 1010.85461  196.28621 100.0000
```

Compound 13b

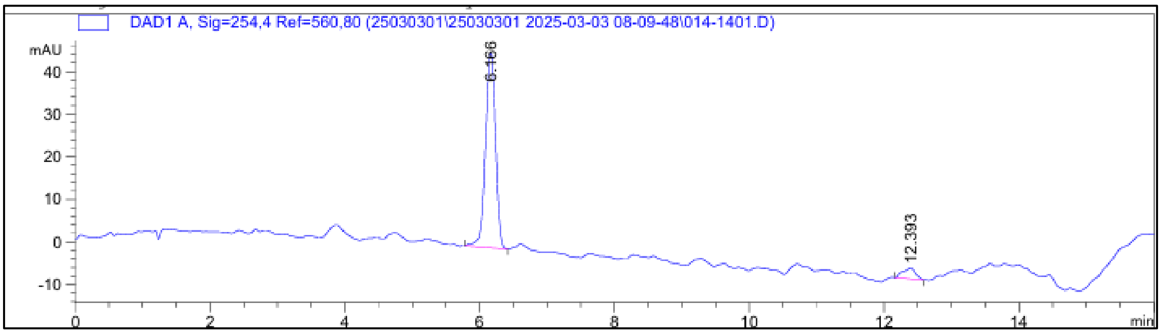


```
=====================================================================
                         Area Percent Report
=====================================================================

Sorted By             :      Signal
Multiplier:                  :      1.0000
Dilution:                    :      1.0000
Use Multiplier & Dilution Factor with ISTDs


Signal 1: DAD1 A, Sig=254,4 Ref=560,80
```

Peak #	RetTime [min]	Type	Width [min]	Area [mAU*s]	Height [mAU]	Area %
1	6.166	BB	0.1623	472.50555	45.80575	93.2232
2	12.393	BB	0.1864	34.34830	2.62960	6.7768

Compound 13c

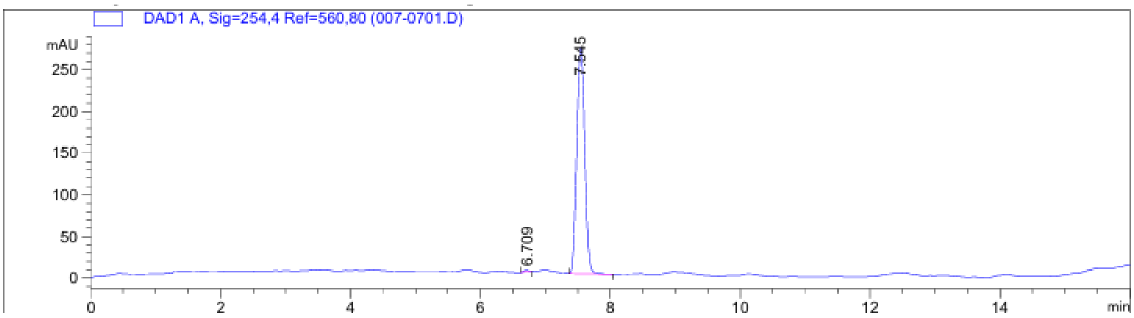


```
=====================================================================
                         Area Percent Report
=====================================================================

Sorted By             :      Signal
Multiplier:                  :      1.0000
Dilution:                    :      1.0000
Use Multiplier & Dilution Factor with ISTDs


Signal 1: DAD1 A, Sig=254,4 Ref=560,80
```

Peak #	RetTime [min]	Type	Width [min]	Area [mAU*s]	Height [mAU]	Area %
1	6.709	BB	0.0722	8.36301	1.86755	0.3573
2	7.545	BB	0.1363	2331.92749	271.37299	99.6427

Compound 13d

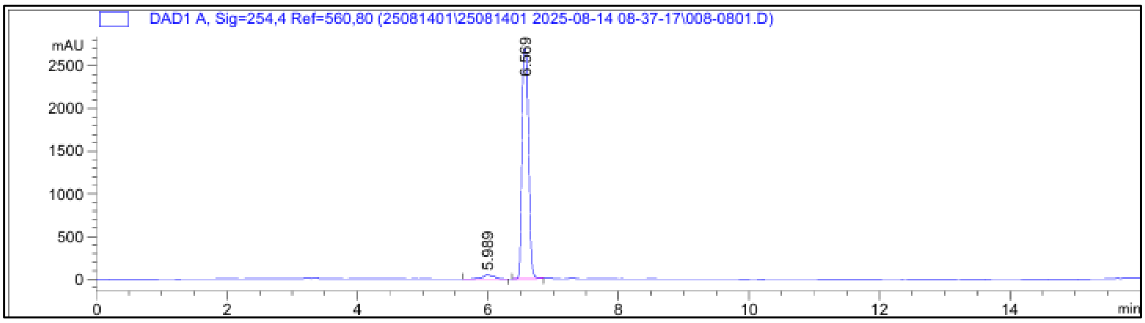


```
=====================================================================
                    Area Percent Report
=====================================================================

Sorted By             :      Signal
Multiplier:                  :       1.0000
Dilution:                    :       1.0000
Use Multiplier & Dilution Factor with ISTDs


Signal 1: DAD1 A, Sig=254,4 Ref=560,80

Peak RetTime Type  Width     Area      Height     Area
  #   [min]        [min]   [mAU*s]     [mAU]        %
----|-------|----|-------|----------|----------|--------|
   1   5.989 BB    0.1487  521.17358   47.98708   2.7247
   2   6.569 BB    0.1124 1.86064e4 2700.06836  97.2753
```

Compound 13e

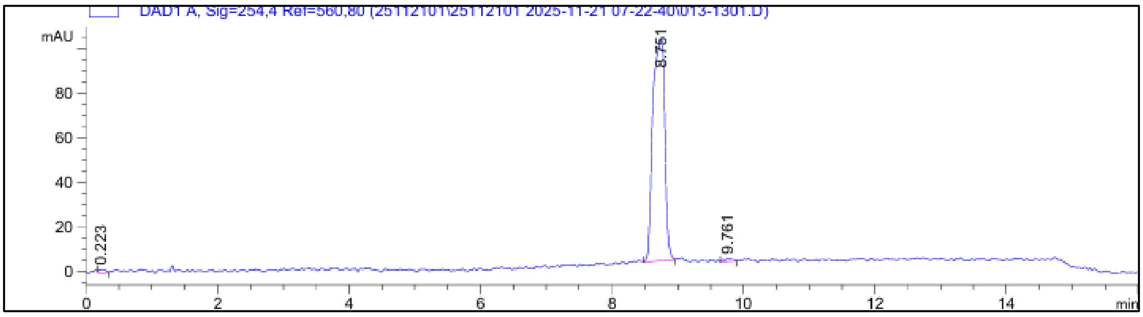


```
=====================================================================
                    Area Percent Report
=====================================================================

Sorted By             :      Signal
Multiplier:                  :       1.0000
Dilution:                    :       1.0000
Use Multiplier & Dilution Factor with ISTDs


Signal 1: DAD1 A, Sig=254,4 Ref=560,80

Peak RetTime Type  Width     Area      Height     Area
  #   [min]        [min]   [mAU*s]     [mAU]        %
----|-------|----|-------|----------|----------|--------|
   1   0.223 VB    0.0851    9.42963    1.50412   0.7388
   2   8.751 BB    0.1735 1256.40491   99.39807  98.4404
   3   9.761 BB    0.1164   10.47573    1.14906   0.8208
```

Compound 13f

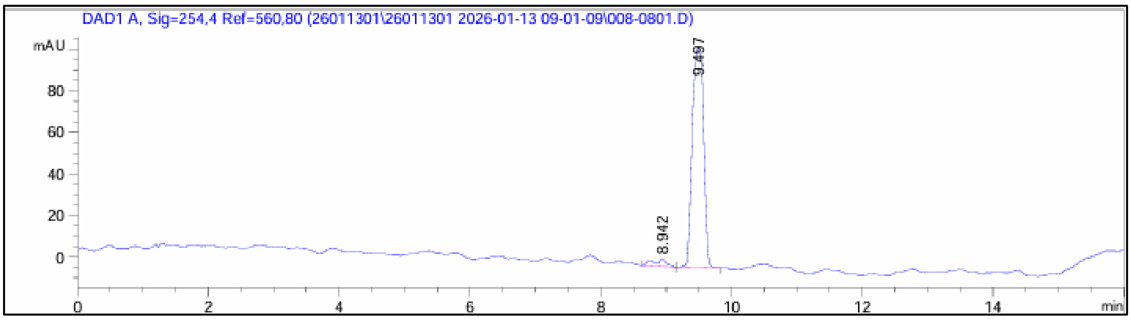

Area Percent Report

Sorted By : Signal
Multiplier: : 1.0000
Dilution: : 1.0000
Use Multiplier & Dilution Factor with ISTDs

Signal 1: DAD1 A, Sig=254,4 Ref=560,80

Peak #	RetTime [min]	Type	Width [min]	Area [mAU*s]	Height [mAU]	Area %
1	8.942	BB	0.1816	52.39964	3.73644	3.9504
2	9.497	BB	0.2025	1274.04956	105.78301	96.0496

Compound 15

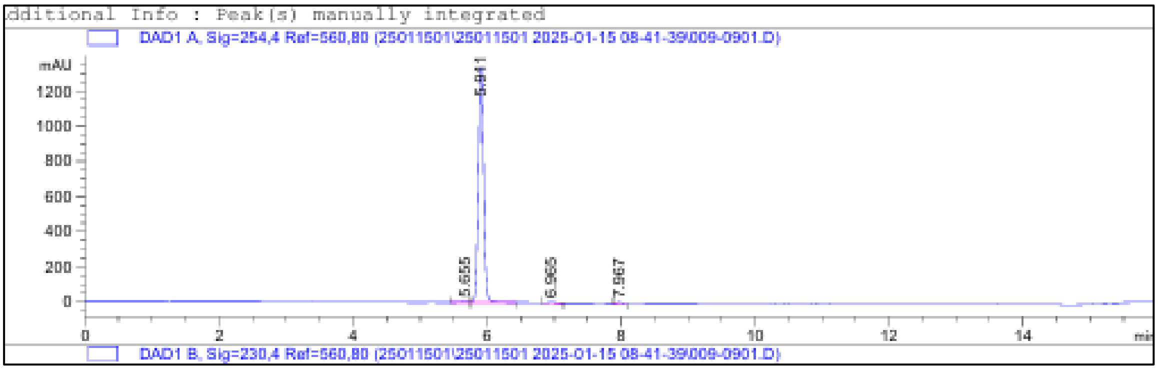

Signal 1: DAD1 A, Sig=254,4 Ref=560,80

Peak #	RetTime [min]	Type	Width [min]	Area [mAU*s]	Height [mAU]	Area %
1	5.655	BV	0.0828	28.56633	5.31079	0.3850
2	5.911	VB	0.0841	7341.55078	1337.12878	98.9575
3	6.965	BB	0.0878	27.76184	4.92184	0.3742
4	7.967	BB	0.0791	21.01212	4.14901	0.2832

Totals : 7418.89107 1351.51042

4. ESI-MS data of the final compounds

ESI-MS data of the final compounds showing calculated and observed m/z values for the molecular ions

ID	Calc. mass	Found mass	Data
**2a**	372.23	$[M+H]^+$ =373.1	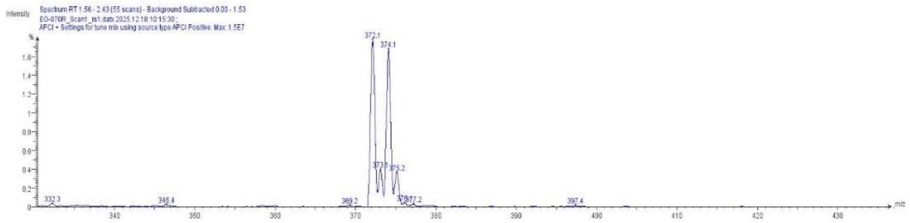
**2b**	310.78	$[M+Na]^+$ =333.0	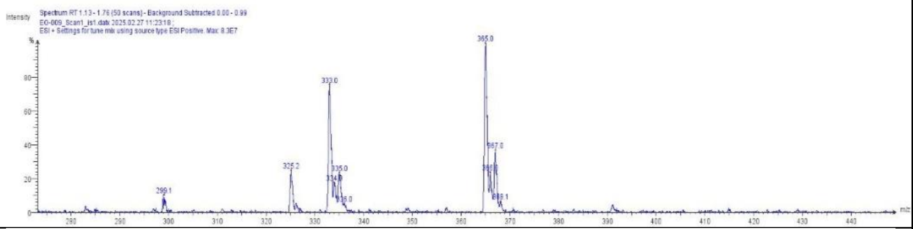
**2c**	368.45	$[M+Na]^+$ =391.2	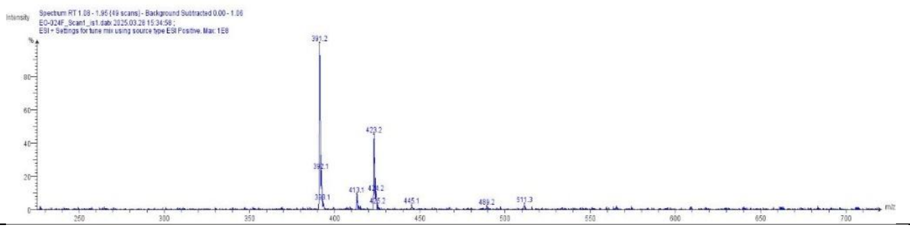
**2d**	290.36	$[M+Na]^+$ =313.1	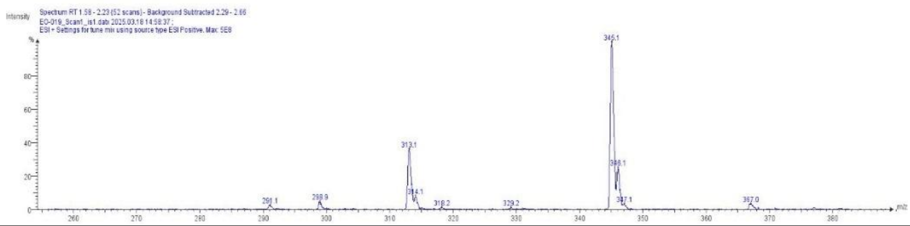
**2e**	315.37	$[M+Na]^+$ =338.3	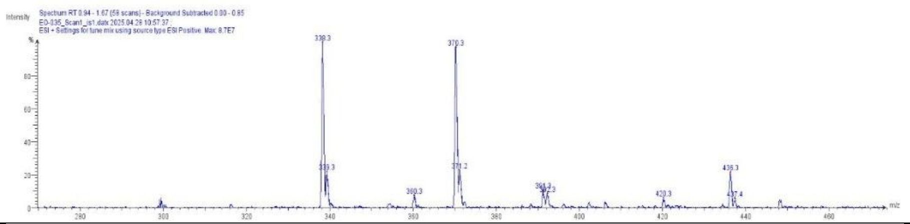
**2f**	314.14	$[M+Na]^+$ =337.2	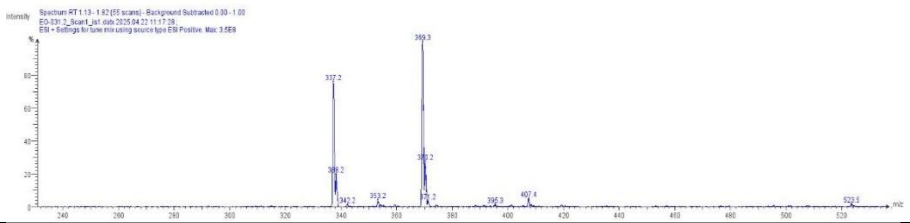
**2g**	326.39	$[M+Na]^+$ = 349.2	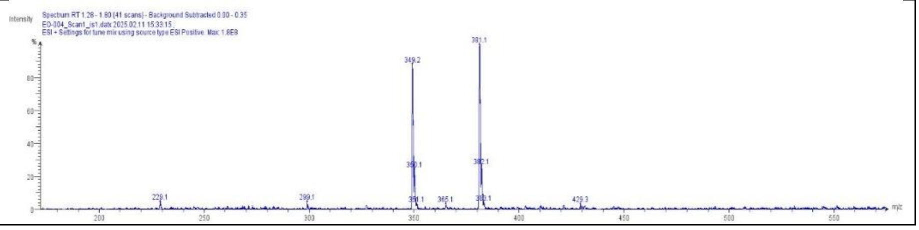

**2h**	326.39	$[M+H]^+$ = 327.2	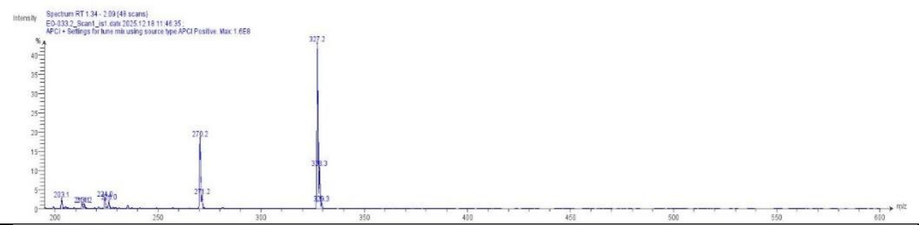
**2i**	326.14	$[M+Na]^+$ = 349.4	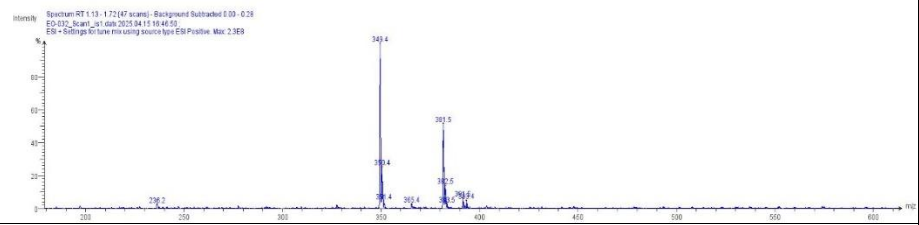
**2j**	326.39	$[M+Na]^+$ = 349.2	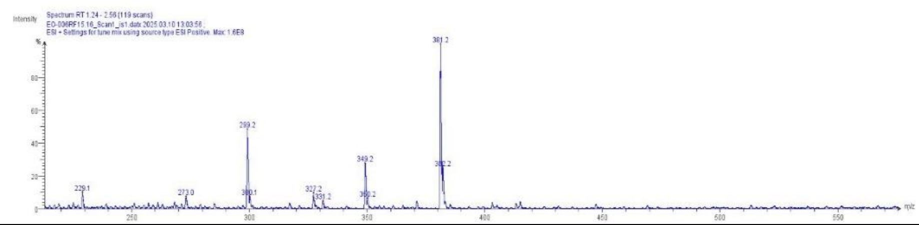
**2k**	363.45	$[M+Na]^+$ = 386.2	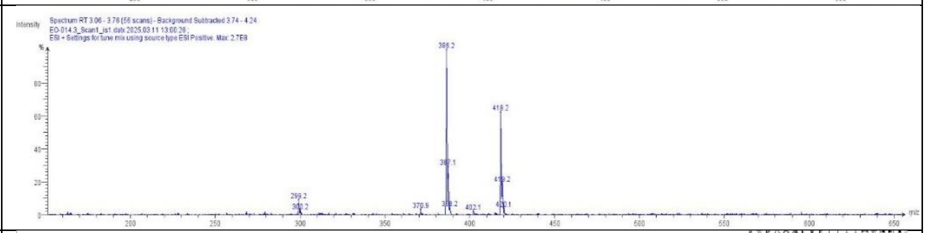
**2l**	416.3	$[M+H]^+$ = 416.3	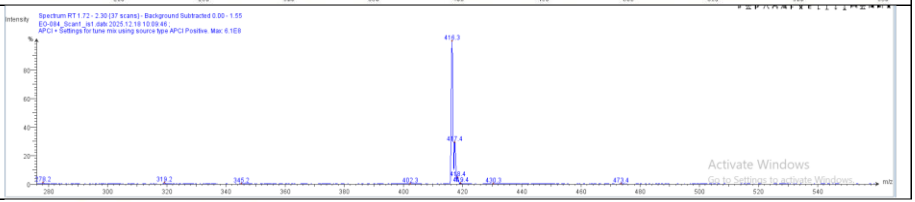
**2m**	418.53	$[M+Na]^+$ = 441.2	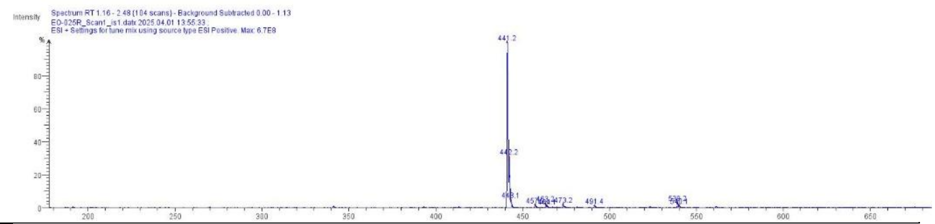
**2n**	318.41	$[M+Na]^+$ = 341.3	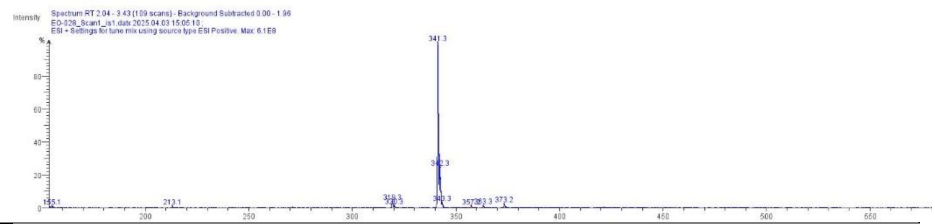
**2o**	434.22	$[M+Na]^+$ = 457.2	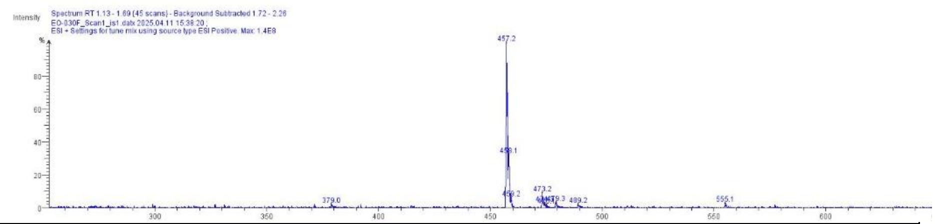

**2p**	334.41	$[M+Na]^+$ = 357.4	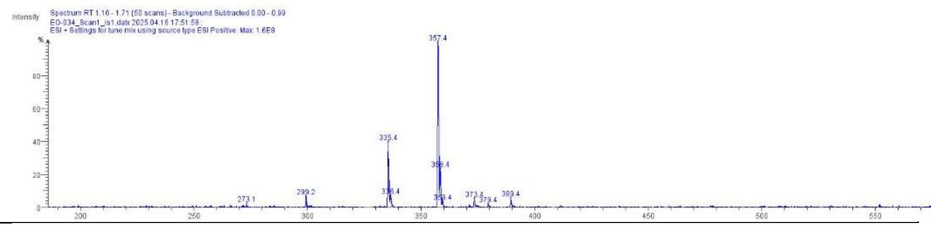
**2q**	477.57	$[M+Na]^+$ = 500.5	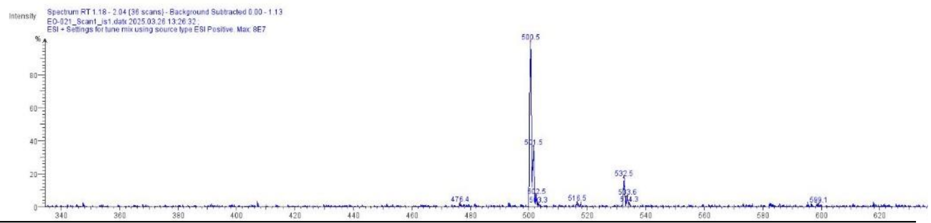
**2r**	377.45	$[M+Na]^+$ = 400.2	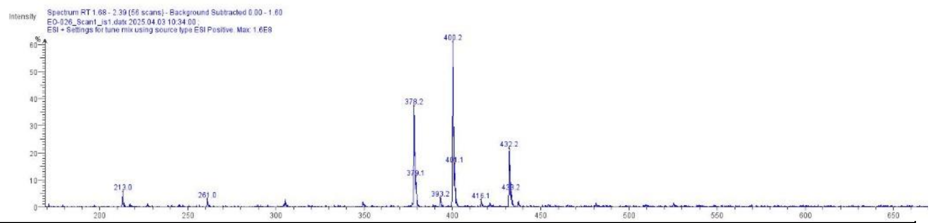
**2s**	459.58	$[M+Na]^+$ = 482.4	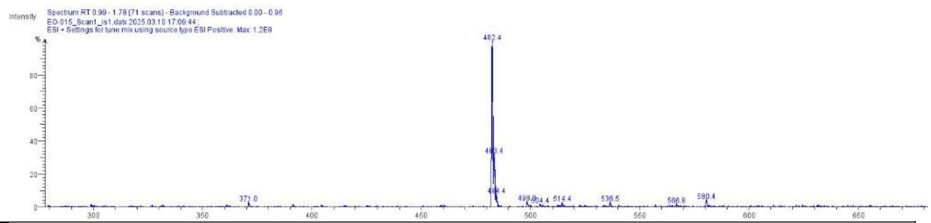
**2t**	359.46	$[M+H]^+$ = 360.1	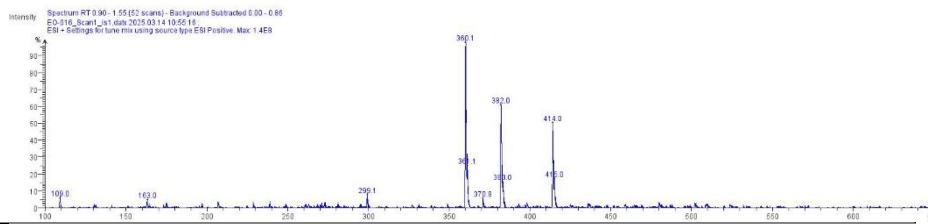
**5a**	359.46	$[M+Na]^+$ = 382.1	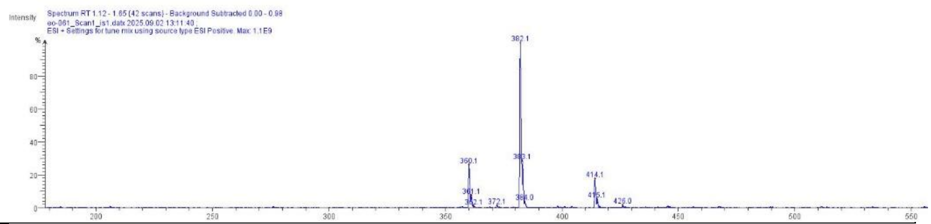
**5b**	414.34	$[M+Na]^+$ = 436.0	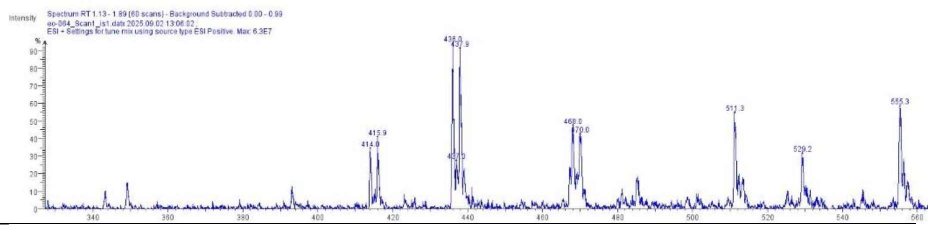
**5c**	471.49	$[M+Na]^+$ =	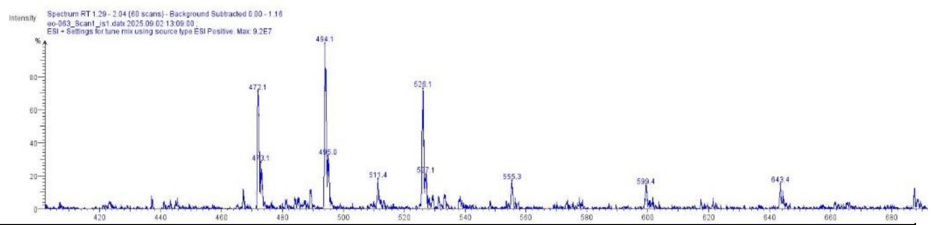

**5d**	331.41	$[M+Na]^+$ = 354.0	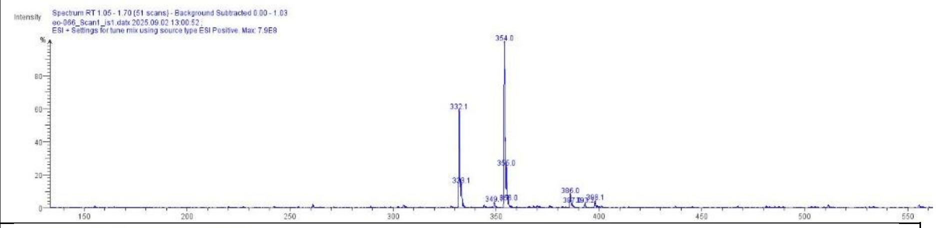
**10**	378.14	$[M+Na]^+$ = 401.1	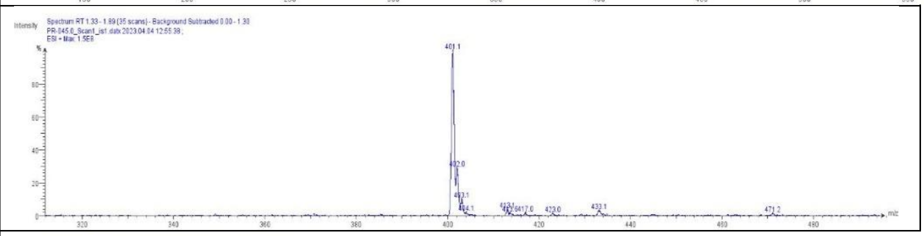
**13a**	195.65	$[M+Na]^+$ = 218.0	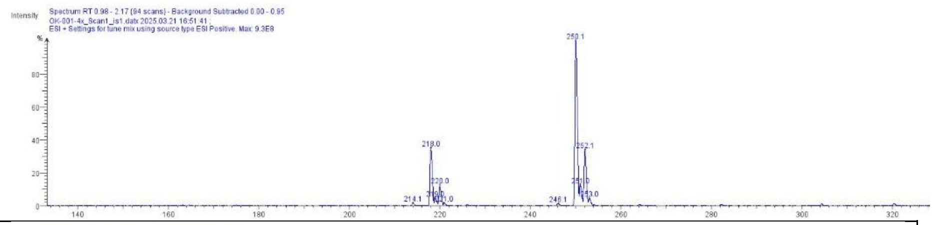
**13b**	274.54	$[M+Na]^+$ = 297.9	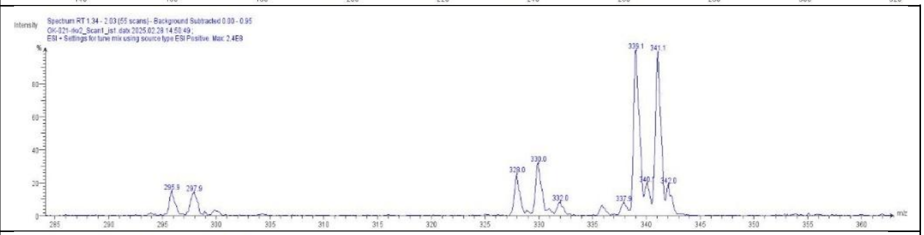
**13c**	271.74	$[M+Na]^+$ = 294.1	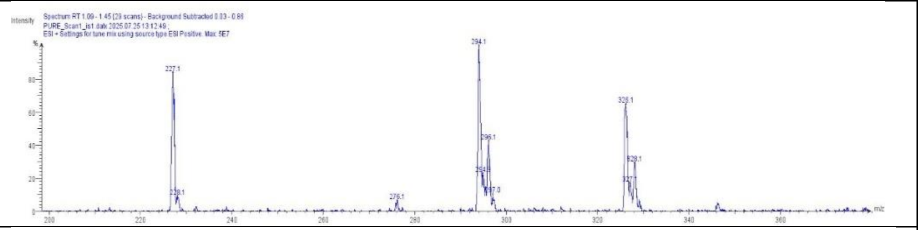
**13d**	358.72	$[M+Na]^+$ = 381.2	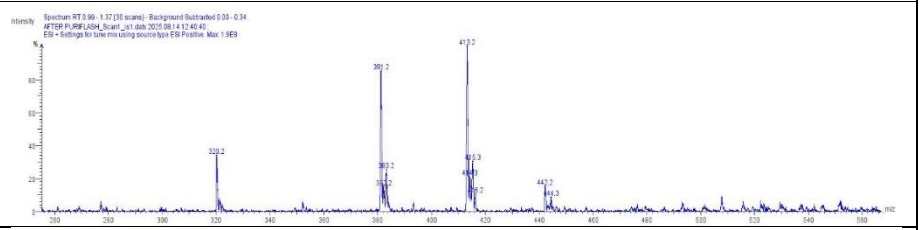
**13e**	267.75	$[M+H]^+$= 268.3	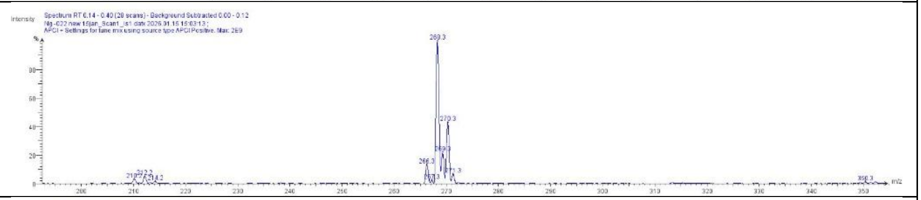
**13f**	307.82	$[M+H]^+$ =308.4	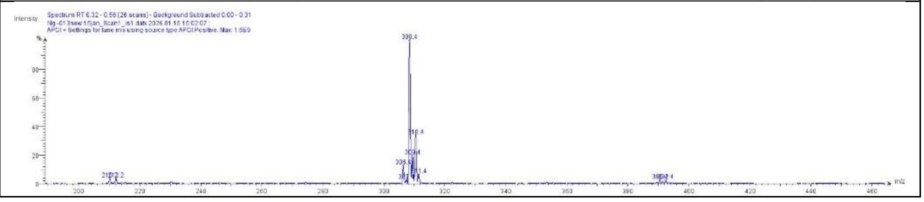

**15**	193.63	[M+Na+H]$^+$ = 217.8	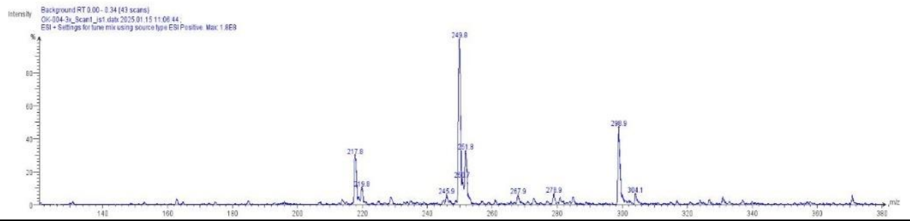
